# Interobserver agreement in Reception and Risk Stratification in Obstetrics implementation

**DOI:** 10.1590/0034-7167-2023-0361

**Published:** 2024-12-13

**Authors:** Manuela Beatriz Velho, Luciana Santos Pimentel, Fernanda Amâncio Soares da Silva, Alberto Trapani, Thayná Ventura, Adaiana Fátima Almeida, Roberta Costa, Roxana Knobel

**Affiliations:** I Universidade Federal de Santa Catarina. Florianópolis, Santa Catarina, Brazil

**Keywords:** Obstetrics, User Embracement, Triage, Risk Assessment, Emergencies, Obstetricia, Acogimiento, Triaje, Medición de Riesgo, Emergencias

## Abstract

**Objectives:**

to analyze interobserver agreement in the Reception and Risk Stratification in Obstetrics protocol implementation.

**Methods:**

a cross-sectional study carried out during Reception and Risk Stratification in Obstetrics implementation, conducted in a tertiary hospital in southern Brazil with 891 participants in January 2020. Descriptive and interobserver agreement analysis was carried out using the Kappa coefficient in the risk stratification assigned by the triage nurse and reviewed by the researcher.

**Results:**

around half of the calls (55.6%) were stratified as not very urgent (green), followed by urgent (yellow) (31.8%), very urgent (orange) (9.3%), not urgent (blue) (3.4%) and no emerging stratification (red). Agreement analysis of revised stratification found Kappa values of 0.20 (blue), 0.54 (green), 0.77 (yellow) and 0.80 (orange).

**Conclusions:**

most appointments were non-urgent. The agreement analysis between the revised and assigned risk stratification revealed greater interobserver agreement as the priority level increased.

## RESUMEN

**Objetivos:**

analizar la concordancia interobservador en la implementación del protocolo de Acogida y Clasificación de Riesgos en Obstetricia.

**Métodos:**

estudio transversal, realizado durante la implementación de la Clasificación de Acogida y Riesgo en Obstetricia, realizado en un hospital terciario del sur de Brasil con 891 participantes en enero de 2020. Se realizó un análisis descriptivo y de concordancia interobservador utilizando el coeficiente Kappa en la clasificación de riesgo asignado por el enfermero clasificador y revisado por el investigador.

**Resultados:**

alrededor de la mitad de las llamadas (55,6%) se clasificaron como no muy urgentes (verde), seguidas de urgentes (amarillo) (31,8%), muy urgentes (naranja) (9,3%), no urgentes (azul) (3,4%) y sin clasificación emergente (rojo). El análisis de concordancia de la clasificación revisada encontró valores Kappa de 0,20 (azul), 0,54 (verde), 0,77 amarillo y 0,80 (naranja).

**Conclusiones:**

la mayoría de las consultas no fueron muy urgentes. El análisis de concordancia entre la clasificación de riesgo revisada y asignada reveló una mayor concordancia interobservador a medida que aumentaba el nivel de prioridad.

## INTRODUCTION

Pregnancy and the postpartum period are natural phenomena of the female reproductive cycle, in which significant physiological changes occur and which in most cases progress without complications. However, some cases may present obstetric complications, resulting in maternal morbid conditions and, eventually, fetal repercussions, even in previously healthy people. In these cases, care in emergency units is necessary, and reception by healthcare professionals and obstetric risk stratification are essential^(1-3)^.

Care for women in the pregnancy-puerperal cycle must be qualified and agile, in order to promote effective care to physiological demands, such as childbirth, but mainly to promote actions that avoid or reduce damage to the dyad in pathological cases. It is evident that failures to identify the severity of the condition increase the risk of maternal morbidity and mortality, and therefore, as an alternative, obstetric triage appears to minimize this outcome^(1,3-5)^.

Triage is a technology developed in clinical emergencies that aims to prioritize care based on patients’ health conditions, their need for medical attention and healthcare service resources. The triage system must be fast, easy to apply and have strong power to predict severity, evolution and use of resources. Implementing tools for this purpose contributes to greater quality of care, organization of care flow and use of resources, in addition to providing timely care and reducing waiting times^(1,4-6)^.

Obstetric emergencies are the gateway for pregnant and postpartum women seeking care due to physiological changes typical of pregnancy or morbid processes. The American College of Obstetricians and Gynecologists recommends the application of risk stratification to this population. Prioritizing care as opposed to first-come, first-served care is necessary not only due to the overcrowding of healthcare services and the demand that exceeds professional capacity, but also to establish parameters and care flows that qualify the care provided, in addition to bringing satisfaction to users and customer service staff^(4-10)^.

Although general triage is strongly established in emergency services, there is no consensus in the literature on the criteria that should be applied in obstetric triage. It is evident that obstetric triage, when compared to general triage, is considered more complex, as it requires assessment of physiological changes associated with pregnancy as well as analysis and consideration of both maternal and fetal aspects^(4,8)^.

Due to obstetric specificities, several scales and flowcharts have been developed internationally. The best known are the Obstetric Triage Acuity Scale (OTAS), which stratifies patients into five levels of severity and which appears to reduce waiting time for care, the Swiss Emergency Triage Scale (SETS), which stratifies patients into four levels, and the Maternal Fetal Triage Index (MFTI), which stratifies pregnant women into five levels. It is highlighted that they are all valid for the population studied^(4,8)^.

In Brazil, the Ministry of Health (MoH) launched the Stork Network in 2011, with the aim of meeting the Millennium Development Goals proposed by the World Health Organization (WHO) to reduce maternal and child morbidity and mortality. Stork Network established, among other actions, the implementation and operation of Reception and Risk Stratification (R&RS) in Obstetrics^(1)^. The R&RS was developed based on the theoretical work of a multidisciplinary team from the MoH and established flowcharts that guide healthcare professionals in prioritizing care by severity, allowing for more equitable care to the population. In the Brazilian MoH protocol, five levels of severity are determined, with ideal times for caring for pregnant women according to a protocol structured based on different complaints, which is based on vital data and symptoms presented by patients^(1)^.

In 2020, a university hospital located in southern Brazil implemented the R&RS in Obstetrics protocol proposed by the Brazilian MoH to organize the obstetric emergency sector. It is understood that protocol implementation is an opportune moment for evaluative monitoring and operational adjustments.

## OBJECTIVES

To analyze interobserver agreement in the Reception and Risk Stratification in Obstetrics protocol implementation.

## METHODS

### Ethical aspects

The study complied with the Brazilian National Health Council Resolution 66/2012, and was approved under the Certificate of Presentation for Ethical Consideration (*Certificado de Apresentação para Apreciação Ética*), issued by a Research Ethics Committee registered on *Plataforma Brasil*. Consent to participate in the research was obtained prior to data collection in service records, through direct contact between the researcher and the women while they were awaiting medical care in the obstetric emergency waiting room or during hospitalization in the hospital ward, with signing of the Informed Consent Form. To guarantee participant confidentiality and anonymity, numeric codes were used in database extraction and preparation based on service records.

### Study design

This is an observational, cross-sectional study, carried out during R&RS in Obstetrics implementation, with retrospective data collection, through records contained in obstetric emergency care records in the maternity ward of a university hospital located in southern Brazil. The study followed the STrengthening the Reporting of OBservational studies in Epidemiology (STROBE) guidelines.

### Period

Data collection was retrospective and took place between May and August 2020, and was carried out based on the records of appointments in the obstetric emergency for January 2020. The researchers had access to records, after completing care, in the hospital’s medical records archiving sector. There was no interference during data collection on the treatment or care provided to users in the service.

### Local

The study location is characterized as a tertiary university hospital, which exclusively assists the Brazilian Health System users, a public and universal Brazilian system, located in southern Brazil. The institution is a reference in highly complex obstetric care in the State.

### Population and sample

The study population consisted of records of appointments in the obstetric emergency in January 2020, a period that corresponded to the month of implementation of R&RS in Obstetrics in the researched institution. For sample selection, delimited by time, the inclusion criteria were care records of pregnant women aged 18 years or over. The exclusion criterion was the absence of a risk stratification record in the service record. In January 2020, 1,038 service records were identified; of these, 79 were excluded due to care for non-pregnant women and another 68 were excluded due to the absence of a record of assessment or risk stratification by R&RS in Obstetrics nurses. The final sample consisted of 891 participants.

### Study protocol

A R&RS in Obstetrics service implementation, based on the MoH protocol, was preceded by training triage nurses and included the participation of professional nurses and doctors who worked in the maternity ward. At the end of the training, five nurses were assigned to perform R&RS in Obstetrics at the investigated hospital, of which two had experience in obstetrics, four were female and one was male.

The MoH protocol for the R&RS in Obstetrics service determines that patients are stratified according to clinical severity, based on the main complaint and brief physical examination, consisting of 12 flowcharts that establish the best stratification based on the clinical status of the condition. user^(1)^. The flowcharts for risk stratification are: fainting/general malaise; abdominal/low back pain/uterine contractions; headache/dizziness/vertigo; shortness of breath/respiratory symptoms; fever/signs of infection; nausea and vomiting; loss of vaginal fluid/secretions; vaginal blood loss; urinary complaints; arrest/reduction of fetal movements; report of seizure and other complaints/situations^(1)^.

According to risk stratification, patients are categorized into five levels/flow of care, identified using colors, which determine the time for assessment by care team, namely: red (emergent), risk of death, immediate care; orange (very urgent), critical or semi-critical condition not stabilized, care within 15 minutes; yellow (urgent), stabilized critical or semi-critical condition, service within 30 minutes; green (not very urgent), with no risk of harm, service in 120 minutes; blue (not urgent), not serious, service in 240 minutes^(1)^.

In the researched institution, the service record takes place on printed material, called service form, and is filled out manually. To implement R&RS, adjustments were made to the care record in the obstetric emergency of the maternity ward, with the insertion of a space for a brief description of the anamnesis carried out by triage nurses as well as the delimitation of a space for recording vital signs, the complaint main and brief investigation of physical examination.

To collect primary information from service records, data were collected in an online form developed in a virtual tool available on Google Forms^®^ with the variables of interest. The data collection form was tested before application and adjustments were made to the way it was filled out, in order to avoid data loss.

The variables analyzed were: sociodemographic and obstetric (age, origin, number of pregnancies, trimester and comorbidities [none, diabetes mellitus, hypertensive syndrome, thyroid disease, HIV, syphilis, smoking, asthma/pneumopathy, psychiatric disease, recurrent urinary infection, others]); application of R&RS protocol (measurement of vital signs, measurement of other signs and symptoms, assigned risk stratification (ARS) by a triage nurse [red, orange, yellow, green, blue], revised risk stratification (RRS) [red, orange, yellow, green, blue], time elapsed between arrival at the hospital and application of R&RS protocol and/or medical care); and final outcome (hospital discharge, hospitalization, evasion, did not wait for care, transfer to another unit, not reported). RRS was carried out after collecting data from the service record by one of the researchers - a final-year resident doctor in obstetrics who participated in the training course to implement the protocol - based on records of the main complaint, anamnesis and physical examination recorded by a triage nurse, according to the risk stratification protocol.

### Analysis of results, and statistics

The data collected in Google Forms^®^ were transferred to the Statistical Package for the Social Sciences (SPSS^®^) statistical program. A descriptive analysis of variables detailed above (absolute and relative number, confidence interval and measures of central tendency), as well as an analysis of agreement between the stratification defined by triage nurses and stratification reviewed by one of the researchers, was followed. The frequencies of underand overestimation of the stratification assigned by triage nurses were analyzed according to stratification revised by the researcher. The weighted Kappa index was also applied, adopting the following Kappa values: <0, without agreement; 0 - 0.20, weak; 0.21 - 0.40, reasonable; 0.41 - 0.60, moderate; 0.61 - 0.80, strong; 0.81 - 1.00, perfect^(11)^.

*A priori*, sample delimitation was based on time, i.e., all pregnant women who met the inclusion and exclusion criteria, treated in the maternity hospital’s obstetric emergency, in the first month of R&RS in Obstetrics implementation. To assess the power of the sample in identifying interobserver differences, a sample calculation was carried out after data analysis. The percentage of disagreement between interobserver assessments was considered through the Kappa index, the alpha error and beta error found, and the need for a minimum size of 314 R&RS assessments was verified to conduct this study^(12)^.

## RESULTS

Sociodemographic and obstetric characterization showed an average age of 27.4 years, with a higher prevalence of pregnant women coming from the city where the hospital was located (78.7%), without comorbidities (74.1%) and who were in the third trimester of pregnancy (61.2%) (Table 1).

**Table 1:** Sociodemographic and obstetric characteristics of pregnant women receiving care and risk stratification in obstetrics at a university hospital (N=891), Southern Brazil, 2020

Age in years	Mean (SD)	Mode	Minimum	Maximum
	27.4 (+ 6,0)	25	18	49
	n		% (IC 95%)	
Origin				
Hospital municipality	701		78.7 (75.9 - 81.2)	
Metropolitan Region	138		15.5 (13.3 - 18.0)	
Other regions	23		2.6 (1.7 - 3.9)	
Not reported	29		3.3 (2.3 - 4.6)	
Total number of pregnancies				
First pregnancy	337		37.8 (34.7 - 41.1)	
Second pregnancy	243		27.3 (24.4 - 30.3)	
More than three pregnancies	300		33.7 (30.6 - 36.9)	
Not reported	11		1.2 (0.7 - 2.2)	
Comorbidities				
None	660		74.1 (71.1 - 76.9)	
Diabetes mellitus	60		6.7 (5.3 - 8.6)	
Hypertensive syndromes	44		4.9 (3.7 - 6.6)	
Thyroid disease	11		1.2 (0.7 - 2.2)	
HIV	3		0.3 (0.1 - 1.0)	
Syphilis	5		0.6 (0.2 - 1.3)	
Smoking	10		1.1 (0.6 - 2.1)	
Asthma/pneumopathy	12		1.3 (0.8 - 2.4)	
Psychiatric illness	8		0.9 (0.5 - 1.8)	
Urinary tract infection/pyelonephritis	15		1.7 (1.0 - 2.8)	
Others	55		6.2 (4.8 - 8.0)	
Not reported	8		0.9 (0.5 - 1.8)	
Gestational trimester				
First	172		19.3 (16.8 - 22.0)	
Second	154		17.3 (14.9 - 19.9)	
Third	545		61.2 (57.9 - 64.3)	
Not reported	20		2.2 (1.5 - 3.5)	

The main complaint in the R&RS in Obstetrics flowchart protocol of the Brazilian MoH was abdominal/low back pain/uterine contractions, present in 444 appointments (49.8%) (Table 2).

**Table 2:** Main complaint of pregnant women treated according to the Reception and Risk Stratification in Obstetrics flowchart of the Brazilian Ministry of Health at a university hospital (N=891), Southern Brazil, 2020

Main complaint	n	% (95% CI)
Fainting/general malaise	3	0.3 (0.1-1.0)
Abdominal/low back pain/uterine contractions	444	49.8 (46.5-53.1)
Headache/dizziness/vertigo	27	3.0 (2.1-4.4)
Shortness of breath/respiratory symptoms	3	0.3 (0.1-1.0)
Fever/signs of infection	15	1.7 (1.0-2.8)
Nausea and vomiting	33	3.7 (2.6-5.2)
Vaginal fluid loss/secretions	77	8.6 (7.0-10.7)
Vaginal blood loss	93	10.4 (8.6-12.6)
Urinary complaints	23	2.6 (1.7-3.9)
Arrest/reduction of fetal movements	37	4.1 (3.0-5.7)
Seizure report	0	-
Other complaints/situations	136	15.3 (13.0-17.8)

Regarding care by the emergency medical team, 5.7% (51 calls) were recorded at the start time, of which five were stratified as blue, 34 as green, nine as yellow, and three as orange. The mean time between the arrival of pregnant women at the hospital reception and the medical care team receiving these services was 181 minutes, with a minimum of 24 and a maximum of 420 minutes of waiting.

In the analysis of measurement of vital signs and other signs and symptoms, according to the decision keys in the flowcharts, the following observations were obtained: in 867 pregnant women, systemic blood pressure was measured (97.3%); in 807, heart rate was measured (90.6%); in 696, axillary temperature was measured (78.1%); and in 718, peripheral oxygen saturation was verified (80.6%). Other signs and symptoms assessed were, in five cases, respiratory rate (0.6%); in 48 cases, the Visual Analogue Scale was applied (5.4%); and in 21 consultations, hemoglucotest was performed (2.4%).

Table 3 presents ASS by triage nurses and RSS by the researcher. The R&RS applied by a trained nurse in 891 cases resulted in stratification of 30 (3.4%) cases as blue, 495 (55.6%) as green, 283 (31.8%) as yellow, 83 (9.3%) in orange and there was no stratification in red. In RSS by the researcher, 164 pregnant women were stratified as blue (18.4%), 385 as green (43.2%), 249 as yellow (27.9%), 93 as orange (10.4%), and again, there was no red color stratification.

**Table 3:** Risk stratification assigned and revised for pregnant women treated in Reception and Risk Stratification in Obstetrics at a university hospital (N=891), Southern Brazil, 2020

		Assigned risk stratification					
		**Blue**	**Green**	**Yellow**	**Orange**	**Red**	**Total** **n (%)**
**Revised risk stratification**	Blue	28	129	5	2	0	164 (18.4%)
	Green	2	338	41	4	0	385 (43.2%)
	Yellow	0	21	223	5	0	249 (27.9%)
	Orange	0	7	14	72	0	93 (10.4%)
	Red	0	0	0	0	0	0 (-)
	Total n (%)	30 (3.4%)	495 (55.6%)	283 (31.8%)	83 (9.3%)	0 (-)	891 (100.0%)

Therefore, there was disagreement between ASS and RSS. It is observed, among the discordant cases, that the researcher’s review identified 164 cases allocated in blue (18.4%); of these, 28 cases were concordant with ASS by a triage nurse and 136 cases were discordant. Among the discordant cases, 129 services had been stratified as green, five services as yellow and two services as orange by a triage nurse (Table 3).

Based on ASS by a triage nurse, Table 4 presents the rates of underestimation and overestimation of stratification, compared with RSS by the researcher. It is noted, for instance, that there was an underestimation of 21 service records in yellow stratifications (2.4%), which were stratified in green. Likewise, an overestimation of 136 service records was found in blue stratification (15.3%) by a triage nurse when they were assigned a green stratification in the review.

**Table 4:** Calculation of underestimation and overestimation of assigned and revised risk stratification of pregnant women treated in Reception and Risk Stratification at a university hospital (N=891), Southern Brazil, 2020

	Underestimation of stratification by a triage nurse		Overestimation of stratification by a triage nurse	
	**n**	**%**	**n**	**%**
Blue	-	-	136	15.3
Green	2	0.2	45	5.0
Yellow	21	2.4	5	0.6
Orange	21	0.0	-	-
Red	-	-	-	-
Total	44	2.5	184	20.8

Table 5 shows the agreement between ASS and RSS, with the respective Kappa values weighted by priority level. The results show an increase in the degree of agreement as the degree of priority increases: weak, in blue; moderate, green in color; and strong, in yellow and orange.

**Table 5:** Agreement between assigned and revised risk stratification of pregnant women treated in Reception and Risk stratification at a university hospital (N=891), Southern Brazil, 2020

Priority degree	Color	Stratified files		Discordant stratification		Kappa	*p* value
		n	%	n	%		
1	Blue	30	3.4	2	0.2	0.20	<0.001
2	Green	495	55.6	157	17.6	0.54	<0.001
3	Yellow	283	31.8	60	6.7	0.77	<0.001
4	Orange	83	9.3	11	1.2	0.80	<0.001
5	Red	-	-	-	-	-	-
Total		891	100	230	25.7	0.68	< 0.001

Of all the services assessed by R&RS, it was observed that 574 (64.4%) resulted in medical discharge; 187 (21.0%) led to hospital admissions; 18 (2.0%) pregnant women evaded; 45 (5.1%) did not wait for medical care; three (0.3%) of cases were transferred to other health units; and 64 cases (7.2%) did not have information on the outcome. Among the 891 patients treated in the obstetric emergency, 156 underwent birth (vaginal or cesarean section), which accounts for 17.5% of all appointments analyzed in this study. Furthermore, 45 (5.1%) were admitted to a high-risk ward and 18 (2.0%) underwent uterine evacuation due to miscarriage.

## DISCUSSION

Pregnant women treated in the university hospital1s maternity ward had an average age of 27.4 years, with a higher prevalence of pregnant women coming from the city where the hospital is located, without comorbidities and who were in the third trimester of pregnancy. They sought care, mainly due to complaints of abdominal/low back pain/contractions, being mostly stratified as green, non-urgent care, and were discharged from hospital after care. A similar profile was observed in other locations in southern Brazil. In the state of Rio Grande do Sul, a study conducted in a usual-risk maternity hospital identified that 63.2% of patients sought the service with the main complaint of abdominal/low back pain/uterine contractions, and 64.4% were discharged from hospital^(7)^. In the state of Paraná, in a usual-risk maternity hospital, 31.9% of patients had the same complaint and the prevalence was higher among women between 20 and 29 years old^(13)^.

In this sample, of the 891 visits to the obstetric emergency, 21.0% resulted in hospitalization to treat complications or to resolve pregnancy. Of the total number of services provided at the service, 17.5% resulted in childbirth (vaginal deliveries or cesarean sections), the main reason for pregnant women seeking maternity services. In agreement with other studies, it is seen that the demand in obstetric emergencies in tertiary hospitals is from individuals with little urgency, who could have their requests met in less complex services^(7,14,15)^.

In care assessment in an obstetric emergency in the study conducted in Paraná, 23.2% of care returned to the healthcare service to which they were linked, whether in Primary Healthcare or in the reference service, and the lowest reason for referral of these for the maternity service resulted in hospitalization due to labor (0.02%)^(13)^. The American College of Obstetricians and Gynecologists points out that care in obstetric emergencies can exceed between two and five times the volume of childbirths in a maternity ward, a situation that can lead to overcrowding of services^(16)^.

In the Brazilian reality, several situations were highlighted that describe this phenomenon, including: low connection between pregnant women and Basic Health Units, suggesting a weakness in prenatal care; popular understanding that exams and assessments can be more easily carried out in tertiary services; low health education regarding the physiological changes of pregnancy and measures to remedy or alleviate these symptoms; and feeling of insecurity and fear^(1,7,14)^. This demand, which could be met by Basic Health Units, ends up creating an overload for emergencies. This can result in harm both to patients, since individuals with greater severity may have poor care, and to professionals, who feel overwhelmed and unable to provide adequate care^(7,8,14,15,17-19)^.

Institutions’ inability to absorb demand results in delays in service. The lack of professionals, equipment, inputs and management protocols compromise care^(15,20,21)^. Likewise, the mere implementation of a risk stratification protocol alone does not qualify the work. This soft-hard health technology demands acceptance of complaint brought by women, an expertise of triage nurses in user assessment manifested by an anamnesis and a precise and succinct physical examination, helping to determine the severity and stratification of risk based on the flowcharts established in the protocol^(1,21,22)^.

Based on this, the continuing education and qualification of professionals is essential for the R&RS protocol to be effective^(20,21,23)^. To this end, it is necessary to raise awareness of managers so that they can offer training, updates and training to professionals as well as adequate infrastructure and resources to carry out R&RS in obstetric emergencies^(2,15,21)^.

The results of this study demonstrated a variation in measurement of signs and symptoms, with a higher frequency of measurement for blood pressure and a lower frequency for the Visual Analogue Pain. These data suggest using different criteria by triage nurses, which demonstrates a potential weakness in the protocol application and the need for greater training of triage nurses to apply R&RS^(1)^.

Measurement of vital signs is mandatory component of the flowcharts, which provide the decision-making keys to define the most appropriate stratification for patients’ clinical status. It is important to highlight that triage nurses may have measured vital signs and applied the Visual Analogue Scale, but did not record this information, used in stratification review by the researcher. A study carried out in Belo Horizonte-Brazil, in a general emergency service for adults, found a similar situation regarding measurement of vital signs, with the absence of recording vital data in more than half of cases and with similar findings for measuring pain intensity^(24)^. Measurement and recording of vital signs and symptoms established in the flowcharts, as well as their intensity and duration, are important objectives that contribute to a more accurate stratification of patients and, therefore, to the determination of their severity and priority of care^(24)^.

In this study, a higher percentage of overestimation of severity (20.8%) and a lower percentage of underestimation (2.5%) were observed in stratifications that did not agree with the R&RS. In a study conducted in Sweden, the authors found the opposite, a lower percentage of overestimation in cases (9.3%) and a higher percentage of underestimation in more severe cases (21.1%), both in obstetric triage^(4)^. If, on the one hand, underestimation of severity can bring harm to individuals, as their risk is underestimated and their waiting time for care increases, potentially worsening their health condition, on the other hand, overestimation overloads the emergency service and the care team, as it diverts material, structural and human resource efforts to less severely ill patients^(24-26)^.

Continuing training of triage nurses regarding the institutional stratification protocol and the particular characteristics of the obstetric population, as well as routine intraand interobserver agreement analysis, allows the application of severity stratification to be the most accurate, with a reduction in underestimation and overestimation. In this way, care will be provided in a timely manner and with the necessary resources to meet the demands presented by patients^(2,5,11,25,26)^.

The development and implementation of an obstetric triage system in Sweden, in its initial assessment, demonstrated a good correlation between the need for hospitalization and the level of accuracy of the developed tool^(4)^. In comparison, the review of risk stratification in this study showed greater interobserver agreement for more serious cases, with a strong Kappa index, whereas, for non-urgent cases, the Kappa index was weak. Similar results were found in R&RS assessment both in adult emergency in São Paulo-SP and in pediatric emergency in a study conducted in Fortaleza-Ceará^(25,26)^.

A study conducted in the country, investigated agreement in the application of the MoH protocol in obstetric emergencies in a maternity school in Belo Horizonte-MG, assessed inter-rater agreement with the participation of 20 nurses, ten trained in R&RS and ten untrained. As a result, the Kappa coefficient varied between 0.47 and 0.77, with a tendency towards underestimation in the red and yellow groups, and overestimation in the yellow and green clinical priorities. However, the final level of agreement between trained and untrained nurses was high (0.87), a result that suggests reliability in the use and application of this protocol^(11)^.

In an international study, carried out in the United States, the investigation of the MFTI scale inter-rater reliability, with five priority levels, trained ten nurses to apply the scale and assessed 211 triaged pregnant women. When comparing the stratifications given by participating nurses and the researcher, a greater discrepancy was identified between levels three and four, of lesser severity, with 21 cases (10.0%) being overestimated by nurses and four (1.9%) being underestimated. From this, with the objective of measuring inter-rater agreement, the Kappa coefficient was used. In this study, the weighted Kappa score (0.65) was achieved and the authors concluded that there is reliability in the MFTI scale application^(27)^.

R&RS in Obstetrics implementation at the studied institution involved professionals with varying degrees of experience (from inexperienced to those with expertise in obstetrics), with simultaneous implementation and performance in R&RS in Pediatrics, characteristics that may have compromised knowledge regarding the nuances of the protocol. It is reiterated that professionals’ skills based on clinical experience help in assessing flowcharts and decision-making, whereas inexperience can lead to subjective interpretations. On the other hand, as it involves a population with unique specificities, studies indicate that it is essential for the proper functioning of the “obstetric triage” technology that professionals are experienced and continuously trained in order to enhance the accuracy of risk stratification in pregnant women who arrive at the obstetric emergency, in order to protect the dyad and speed up care for more serious cases^(3,5,7,8,23,28)^.

Training, monitoring and assessment regarding its implementation are essential for the quality of R&RS in services. Information and communication technologies are increasingly being tested and implemented in healthcare. A study that assessed the construction of a decision support system for nurses in carrying out R&RS in Obstetrics, based on the Brazilian MoH protocol, with structured questions and answers based on signs and symptoms presented by pregnant women, obtained a satisfactory adequacy index in assessment by nurses and IT professionals^(29)^.

In the researched institution, the absence of data recording regarding the time to carry out triage and poor recording of the time between risk stratification and assessment by the care team point to weaknesses in protocol implementation. The assessment of these indicators is suggested by the MoH as a quality criterion for implementing and monitoring this tool^(1)^.

The proposal for a specific triage system for pregnant women has the potential to reduce hospitalization time and maternal and neonatal morbidity and mortality^(1)^, but, to achieve this, it is necessary to carry out rigorous monitoring of this tool regarding its implementation and implantation, and, with this, achieve its purpose, which is to prioritize brief assistance for patients with greater severity^(1,16,19,22)^. Thus, clear records must be established, periodic assessment of indicators and continuous improvement of triage nurses’ skills must be established^(1,8,18,30)^.

As a public health policy, Stork Network recognizes the growing importance of obstetric nursing and its impact on women’s and newborns’ health, especially in care during labor and childbirth. Based on the results of this study and the national data presented, we can see the importance of applying R&RS in Obstetrics as a means of improving quality of care, by organizing the flow of care and the use of resources. However, the need for a solid assessment of the accuracy and precision of R&RS in Obstetrics proposed by the MoH is notable.

The results also indicate the need for further progress to be made, as the internationally agreed Millennium Development Goals regarding maternal mortality reduction and again agreed upon in the Sustainable Development Goals have not been achieved. At this moment, the persistent challenges in the Brazilian reality arise especially with regard to expanding access to essential healthcare services and reducing maternal mortality^(31)^. In this aspect, the results of this study play a fundamental role in encouraging dialogue and advancing network care, identifying the most serious cases and impacting maternal and neonatal morbidity and mortality reduction.

### Study limitations

The study faced limitations due to the operationalization of data collection, retrospectively, with information from medical records; therefore, information bias is assumed in the eventual lack of complete records in the service form. Another limitation is due to the absence in the recording of the time between the arrival of pregnant women at hospital reception and the appointment to perform R&RS as well as the time of service by the medical team, making it impossible to analyze these data.

However, it is an initial study carried out in a maternity hospital with exclusive care provided by the Brazilian Health System, which brought contributions to the area, by describing the characteristics of patients treated, pointing out weaknesses in the implementation and implantation of the R&RS in Obstetrics protocol proposed by the Brazilian MoH as well as interobserver disagreements regarding the stratification application.

### Contributions to nursing, health and public policy

It is believed that the appropriate use of R&RS in Obstetrics may reduce adverse obstetric and neonatal outcomes, with a view to prioritizing severe cases, but this statement cannot be concluded when conducting a non-interventional study and given the difficulties described in implementation of this tool in the institution. However, the study highlighted the need for continuous assessment of both the R&RS in Obstetrics protocol implementation and the need to train triage nurses in risk assessment and stratification of these patients.

This analysis also showed that the search for care for non-urgent or non-urgent patients in a tertiary and highly complex hospital requires greater agreement between the different levels of care in order to avoid overcrowding in the obstetric emergency and, as a result, poor care for serious and urgent patients. These observations were shared with the maternity service management to implement improvements in the service.

## CONCLUSIONS

This study identified a higher prevalence of pregnant women without comorbidities in the third trimester of pregnancy who sought emergency care complaining of abdominal/low back pain/contractions in the clientele treated at a tertiary level university hospital. In ASS, practically half of appointments were stratified as green (not very urgent), and the majority resulted in hospital discharge, which demonstrates that pregnant women could be treated in less complex services. Only a fifth of the services involved childbirth (vaginal deliveries or cesarean sections), with this being the main reason for pregnant women seeking the services of a maternity ward. Agreement analysis between RSS and ASS revealed greater interobserver agreement as severity increased from non-urgent to urgent cases.

## Supplementary Material

0034-7167-reben-77-05-e20230361-suppl01

## Data Availability

https://doi.org/10.48331/scielodata.6L3OWP
