# Supplementary material for: Interobserver agreement in Reception and Risk Stratification in Obstetrics implementation
Source: Rev Bras Enferm. 2024 Dec 13;77(5):e20230361. doi: 10.1590/0034-7167-2023-0361 (PMC11654229; doi:10.1590/0034-7167-2023-0361)
Supplement: 0034-7167-reben-77-05-e20230361-suppl01 [file 0034-7167-reben-77-05-e20230361-suppl01.pdf]

Banco de Dados do manuscrito submetido à REBEn-2023-0361: Implantação do Acolhimento e Classificação de Risco em Obstetrícia num hospital terciário

Informações de contato: Manuela Beatriz Velho, Universidade Federal de Santa Catarina, e-mail: manuela.nfr.ufsc@gmail.com (Pesquisador Correspondente)

Data da coleta de dados: coleta de dados realizada entre maio e agosto de 2020 de forma retrospectiva a partir das fichas de atendimento realizadas no Serviço de Emergência Obstétrica do Hospital Universitário Polydoro Ernani de São Thiago (HUPEST) da Universidade Federal de Santa Catarina (UFSC) sob

As variáveis investigadas foram: sociodemográficas e obstétricas (idade, procedência, número de gestações, trimestralidade e comorbidades [nenhuma, diabetes mellitus, síndrome hipertensiva, doença da tireoide, HIV, sífilis, tabagismo, asma/pneumopatia, doença psiquiátrica, infecção urinária recorrente, outras]); aplicação do protocolo A&CR (aferição dos sinais vitais, aferição de outros sinais e sintomas, classificação de risco atribuída pelo enfermeiro classificador [vermelha, laranja, amarela, verde, azul], classificação de risco revisada [vermelha, laranja, amarela, verde, azul], tempo decorrido entre a chegada ao hospital e a aplicação do protocolo A&CR e/ou o atendimento médico) e desfecho final (alta hospitalar, internação, evasão, não aguardou atendimento, transferência para outra unidade, não informado). A classificação de risco revisada foi realizada por um dos pesquisadores com base nos registros da queixa principal, anamnese e exame físico do enfermeiro classificador, conforme o protocolo de classificação de risco.

1- Código do paciente em sequência numérica	2- IDADE EM ANOS COMPLETOS	3 - PROCEDÊNCIA 1 - Florianópolis; 2 - Núcle	4 - NÚMERO DE GESTAÇÕES INCLUIND	5 - TRIMESTRE GESTACIONAL 1 - PRIM	6 - IDADE GESTACIONAL EM SEMANAS	7 - COMORBIDADES 1- NENHUMA; 2 -	8 - DATA DA ADMISSÃO NA EMERGêNCIA EM FORMATO XX/XX/XXXX	9 - HORA DA ADMISSÃO NA EMERGÊNCIA	10 - FOI AFERIDO PRESSÃO ARTERIAL NA	11 - VALOR DA PRESSÃO ARTERIAL SISTÓ	12 - VALOR DA PRESSÃO ARTERIAL DIAS	13 - AFERIDA FREQUÊNCIA CARDÍACA NA	14 - VALOR DA FREQUÊNCIA CARDÍAC	15 - AFERIDA A FREQUÊNCIA RESPIRATÓ	16 - VALOR DA FREQUÊNCIA RESPIRATÓ	17 - AFERIDA A TEMPERATURA NA ADMI	18 - VALOR DA TEMPERATURA AXILAR	19 - PRESENÇA DE QUEIXA DE DOR NA	20 - FOI UTILIZADA A ESCALA ANALÓGI	21 - VALOR NA ESCALA ANALÓGICA	22 - AFERIDA A SATURAÇÃO PERIFÉRIC	23 - VALOR DA SATURAÇÃO PERIFÉRIC	24 - AFERIDO A GLICEMIA CAPILAR	25 - VALOR DA GLICEMIA CAPILAR	26 - CLASSIFICAÇÃO DE RISCO REVISADA	27 - CLASSIFICAÇÃO DE RISCO ATRIBUÍD	28 - QUEIXA PRINCIPAL REGISTRADA	29 - O DESCRITOR ADOTADO FOI	30 - TEMPO EM MINUTOS DECORRIDOS	31 - TEMPO EM MINUTOS DECORRIDO ENTR	32 - HAVIA REGISTRO DO DIAGNÓSTICO	33 - HOUVE CONCORDÂNCIA ENTRE A	34 - QUAL O DESFECHO DO ATENDIME	35 - HORA DA CONCLUSÃO DO ATENDIME	36 - DATA DA CONCLUSÃO DO ATENDIMENTO NA EMERGÊNCIA NO FORMAT	37 - PODE-SE DETERMINAR O TEMPO EM MINU	38 - A AVALIAÇÃO MÉDICA OCORRE	39 - PARA AS PACIENTES COM INTERNA
1	36	2	2	3	40	1	1/5/2020	00:30	1	130	86	1	89	2	999	1	36.2	1	2	999	2	999	2	999	4	4	2	1	999	160	2	2	2	999	999	2	2	1
2	30	1	3	1	10	1	1/8/2020	18:00	1	106	59	2	999	2	999	2	999	1	2	999	2	999	2	999	2	2	2	1	999	999	2	2	1	999	999	1	3	
3	28	1	3	2	23	1	1/3/2020	15:05	1	85	51	1	105	2	999	2	999	1	1	8	1	100	2	999	4	4	2	1	999	55	2	1	5	09:00	05/01/2020	3	2	
4	24	1	2	3	34	1	1/13/2020	11:35	1	127	78	1	79	2	999	1	36.1	1	1	5	1	100	2	999	3	3	2	1	999	999	1	2	1	999	999	1	3	
5	20	1	3	3	39	1	1/15/2020	23:44	1	103	58	1	88	2	999	1	36.7	999	2		1	100	2	999	3	3	2	1	999	76	1	2	1	999	999	2	2	
6	20	1	3	3	40	1	1/27/2020	21:49	1	100	60	2	999	2	999	2	999	2	2		2	999	2	999	1	2	12	2	999	162	1	2	1	999	999	2	2	
7	36	1	2	3	30	1	1/6/2020	12:04	1	108	59	1	99	2	999	1	35.6	2	2		1	99	2	999	3	3	12	2	999	999	1	2	1	999	06/01/2020	1	3	
8	38	2	3	3	40	1	1/2/2020	08:42	2	999	999	2	999	2	999	2	999	1	1	8	2	999	2	999	4	4	2	1	999	999	2	1	2	999	02/01/2020	1	3	2
9	40	1	2	1	12	1	1/22/2020	09:04	1	116	63	1	77	2	999	1	36.1	1	2	999	1	99	2	999	3	3	2	1	999	999	1	2	2	999	999	1	3	3
10	40	1	2	1	11	1	1/21/2020	16:40	1	107	68	1	67	2	999	1	36.1	1	2	999	2	100	2	999	2	2	8	1	999	144	2	1	1	21/01/	999	2	2	
11	27	1	4	3	37	11	1/16/2020	19:07	1	120	60	2	999	2	999	1	35.4	1	2	999	2	999	2	999	2	2	2	1	999	999	1	2	1	999	999	1	3	
12	27	1	1	3	40	1	1/4/2020	18:00	1	118	67	1	95	2	999	1	37	2	2		1	99	2	999	1	2	12	2	999	999	2	2	1	999	04/01/2020	1	3	
13	27	1	1	3	40	1	1/8/2020	10:15	1	112	77	1	97	2	999	1	36.6	999	2		1	98	2	999	3	3	2	1	999	75	2	2	1	999	08/01/2020	2	2	
14	19	2	1	3	39	1	1/19/2020	14:32	1	116	74	1	107	2	999	1	36	999	2		1	100	2	999	3	3	2	1	999	999	2	2	2	999	19/01/2020	1	3	1
15	32	1	3	3	38	1	1/21/2020	16:00	1	102	60	1	90	2	999	1	35.9	1	2	999	1	99	2	999	3	3	2	1	999	105	2	1	999	999	999	2	2	
16	32	1	2	3	39	1	1/29/2020	18:23	1	116	59	1	95	2	999	1	36.1	2	2		1	99	2	999	3	3	7	1	999	97	1	2	1	999	999	2	2	
17	29	1	2	2	22	1	1/12/2020	18:07	1	117	70	1	125	2	999	1	38.8	1	2	999	1	99	2	999	3	3	5	1	999	82	1	1	1	22:00	12/01/2020	2	2	
18	22	1	1	3	38	1	1/19/2020	17:23	1	106	56	1	101	2	999	1	36.3	999	2		1	99	2	999	2	2	2	1	999	67	1	1	1	999	999	2	1	
19	22	1	1	3	38	1	1/21/2020	21:22	1	117	72	1	105	2	999	1	36.7	1	2	999	1	99	2	999	3	3	2	1	999	999	1	1	2	999	999	1	3	2
20	26	1	1	3	40	12	1/14/2020	11:29	1	119	69	1	93	2	999	1	35.4	2	2		1	99	2	999	1	2	12	2	999	216	2	999	1	999	999	2	2	
21	26	1	1	3	40	1	1/19/2020	23:27	1	115	69	1	103	2	999	2	999	999	2		1	98	2	999	3	3	10	1	999	73	2	2	1	999	999	2	2	
22	26	1	1	3	41	12	1/20/2020	07:22	1	115	70	1	96	2	999	1	36.1	2	2		1	99	2	999	2	2	12	1	999	999	2	1	2	999	999	1	3	2
23	30	3	1	3	38	1	1/2/2020	12:17	2	999	999	2	999	2	999	2	999	2	2		2	999	2	999	1	1	12	1	999	183	2	999	4	15:20	02/01/2020	3	1	
24	23	2	1	3	40	2	1/21/2020	06:46	1	126	73	1	92	2	999	1	36.8	2	2		1	98	2	999	1	2	12	2	999	999	1	2	2	999	999	1	3	2
25	40	1	5	1	12	3	1/21/2020	13:15	1	115	66	1	107	2	999	1	36.6	2	2		1	100	2	999	1	2	12	2	999	999	1	2	1	999	999	1	3	
26	22	1	1	3	40	1	1/13/2020	23:53	1	105	59	1	74	2	999	2	999	1	2	999	2	999	2	999	3	3	2	1	999	999	1	2	1	999	999	1	3	
27	22	1	1	3	39	1	1/13/2020	00:27	1	117	68	1	64	2	999	1	35.9	999	2		1	99	2	999	3	2	2	2	999	999	1	1	1	999	999	1	3	
28	22	1	1	3	40	1	1/14/2020	09:12	1	103	66	1	98	2	999	2	999	999	2		1	100	2	999	3	3	2	1	999	999	2	1	2	999	999	1	3	2
29	27	1	6	2	24	1	1/22/2020	12:05	1	111	69	1	94	2	999	1	35.2	2	2		1	99	2	999	2	2	8	1	999	999	1	1	1	999	999	1	3	
30	26	1	3	3	35	2	1/11/2020	22:07	1	123	70	1	110	2	999	1	37	1	1	3	1	99	2	999	2	2	7	1	999	999	2	2	1	999	999	1	3	
31	26	1	3	3	36	2	1/19/2020	05:41	1	130	70	1	89	2	999	2	999	999	2		2	999	2	999	2	3	2	2	999	119	1	1	1	11:00	19/01/2020	2	1	
32	26	1	3	3	34	2	1/23/2020	06:33	1	115	60	1	73	2	999	2	999	2	2		2	999	1	94	1	2	12	2	999	47	1	2	1	18:00	23/01/2020	2	1	
33	26	1	3	3	38	2	1/30/2020	14:47	1	120	56	1	125	2	999	1	36.9	2	2		1	99	2	999	2	2	7	1	999	143	1	2	1	999	999	2	2	
34	26	1	2	3	39	1	1/10/2020	22:26	2	999	999	2	999	2	999	2	999	999	2		2	999	2	999	4	4	7	1	999	999	1	1	2	999	999	1	3	1
35	30	1	1	2	25	1	1/30/2020	16:51	1	109	65	1	85	2	999	1	36.4	1	2	999	1	99	2	999	1	2	2	2	999	999	1	1	1	999	999	1	3	
36	20	2	1	2	27	4	1/28/2020	07:26	1	103	60	1	76	2	999	1	35.9	1	2	999	1	100	2	999	1	2	2	2	999	999	1	2	1	999	999	1	3	
37	24	3	3	3	39	1	1/16/2020	14:19	1	111	66	1	89	2	999	1	35.9	999	2		1	99	2	999	2	2	2	1	999	176	2	2	1	17:30	16/01/2020	2	2	
38	19	1	1	2	20	1	1/2/2020	21:29	1	106	54	1	76	2	999	1	35.8	2	2		1	99	2	999	2	2	2	1	999	126	2	999	3	01:45	03/01/2020	3	2	
39	19	1	1	3	33	1	1/11/2020	17:00	1	97	59	1	98	2	999	1	36.3	1	2	999	1	99	2	999	2	2	9	1	999	999	1	1	1	999	999	1	3	
40	19	1	1	3	35	1	1/28/2020	09:47	1	107	62	1	83	2	999	1	36.1	1	2	999	1	100	2	999	3	3	8	1	999	999	1	2	1	999	999	1	3	
41	25	1	4	2	13	3	1/21/2020	18:56	1	126	54	1	98	2	999	1	35.9	1	2	999	1	99	2	999	2	2	2	1	999	999	2	2	1	999	999	1	3	
42	19	2	1	3	39	3	1/20/2020	19:28	1	139	75	1	100	2	999	1	37.1	999	2		1	99	2	999	2	2	10	1	999	999	1	2	2	999	999	1	3	2
43	23	2	4	3	36	11	1/10/2020	07:57	1	98	53	1	84	2	999	1	36.2	1	2	999	1	100	2	999	2	2	9	1	999	999	2	1	1	15:45	10/01/2020	1	3	
44	23	2	4	3	37	12	1/20/2020	22:38	1	116	69	1	94	2	999	1	36.8	2	2		1	99	2	999	2	2	10	1	999	107	2	2	1	999	999	2	1	
45	23	2	2	3	39	1	1/31/2020	04:18	1	136	91	1	74	2	999	1	36.6	2	2		2	999	2	999	999	0	0	3	N√O S	999	1	3	2	07:00	31/01/2020	1	4	1
46	24	1	3	3	31	1	1/18/2020	08:28	1	111	73	1	86	2	999	1	36.7	999	2		1	100	2	999	2	2	8	1	999	999	2	1	1	999	999	1	3	
47	24	1	3	3	33	12	1/28/2020	15:13	1	109	63	1	93	2	999	1	37	1	2	999	1	99	2	999	1	2	2	2	999	237	1	1	1	999	999	2	2	
48	24	1	3	3	33	1	1/31/2020	05:21	1	128	74	1	102	2	999	1	36.4	2	2		1	99	2	999	2	3	2	2	999	120	1	1	2	999	999	2	2	1
49	25	1	2	3	32	8	1/29/2020	17:06	1	115	74	1	89	2	999	1	35.9	1	2	999	1	100	2	999	1	2	2	2	999	999	2	2	1	999	999	1	3	
50	22	2	1	3	38	12	1/9/2020	15:30	1	110	61	1	77	2	999	1	36.8	999	2		1	99	2	999	2	2	12	1	999	999	1	1	2	999	999	1	3	2
51	21	1	3	1	11	1	1/19/2020	13:45	1	135	83	1	94	2	999	1	35.6	2	2		1	99	2	999	1	1	12	1	999	999	1	2	1	999	19/01/2020	1	3	
52	21	1	3	1	11	1	1/18/2020	20:51	1	134	89	1	104	2	999	1	36.7	1	2	999	2	999	2	999	2	2	9	1	999	184	1	1	3	01:25	19/01/2020	2	2	

53	19	1	1	2	14	1	1/3/2020	08:08	1	114	58	1	62	2	999	1	36.4	1	1	9	1	99	2	999	4	4	2	1	999	72	2	1	999	999	999	2	2	
54	19	1	1	2	15	1	1/7/2020	20:09	1	100	60	1	70	2	999	2	999	1	2	999	2	999	2	999	2	2	2	1	999	999	2	2	1	999	999	1	3	
55	24	2	2	1	12	1	1/13/2020	20:06	1	90	60	1	70	2	999	2	999	999	2		2	999	2	999	2	2	8	1	999	999	1	1	1	999	999	1	3	
56	39	1	4	1	8	1	1/26/2020	11:38	1	115	74	1	72	2	999	1	36.7	2	2		1	99	2	999	2	2	8	1	999	127	2	2	1	999	999	3	1	
57	30	1	2	3	40	1	1/2/2020	20:28	1	126	77	1	112	2	999	2	999	2	2		1	99	2	999	3	3	2	1	999	999	1	999	2	999	999	1	3	1
58	33	2	1	3	33	1	1/8/2020	06:22	1	90	60	1	71	2	999	2	999	1	2	999	2	999	2	999	2	2	2	1	999	999	2	2	1	999	08/01/2020	1	3	
59	23	1	3	3	32	4	1/5/2020	19:47	1	118	72	1	93	2	999	1	36.1	2	2		1	99	2	999	3	4	2	2	999	999	1	2	1	999	999	1	3	
60	25	1	2	999	999	1	1/12/2020	14:43	1	136	73	1	95	2	999	1	36.5	999	2		1	99	2	999	4	3	8	2	999	999	1	2	999	20:00	12/01/2020	1	3	
61	29	1	4	2	16	1	1/14/2020	09:10	1	102	62	1	96	2	999	1	36.3	1	2	999	1	99	2	999	3	3	2	1	999	999	2	2	1	999	999	1	3	
62	22	1	2	3	39	1	1/9/2020	22:36	1	112	66	2	999	2	999	2	999	1	2	999	2	999	2	999	2	2	2	1	999	999	2	2	2	999	999	1	3	2
63	23	1	2	1	10	1	1/30/2020	10:06	1	102	58	2	999	2	999	1	36.3	2	2		2	999	2	999	2	2	7	1	999	999	2	2	1	999	999	1	3	
64	23	1	999	999	999	1	1/18/2020	02:08	1	143	68	1	92	2	999	1	36.6	1	2	999	1	99	2	999	1	2	2	2	999	999	1	2	1	999	999	1	3	
65	32	1	1	3	39	1	1/22/2020	21:17	1	113	68	1	83	2	999	1	36.4	1	2	999	2	999	2	999	2	2	6	1	999	323	2	1	1	999	999	2	2	
66	26	1	3	3	35	1	1/10/2020	18:43	1	118	66	1	100	2	999	1	36.1	1	2	999	1	99	2	999	2	2	2	1	999	999	1	2	1	999	999	1	3	
67	42	1	7	3	39	2	1/2/2020	21:07	1	113	71	1	82	2	999	1	35.2	2	2		1	100	2	999	2	2	2	1	999	133	1	999	1	999	999	2	2	
68	42	1	7	3	40	2	1/7/2020	12:58	1	117	68	1	73	2	999	1	35.7	999	2		1	99	1	82	3	3	2	1	999	32	2	1	1	5:00:0	43836.00	2	2	
69	24	1	2	3	33	3	1/8/2020	11:25	1	140	78	1	71	2	999	1	36.6	1	2	999	1	99	2	999	4	4	3	1	999	50	2	1	2	999	08/01/2020	2	2	1
70	30	1	2	3	35	2	1/30/2020	06:57	1	89	57	1	88	2	999	2	999	2	2		1	99	1	95	1	2	12	2	999	37	1	2	1	999	999	2	1	
71	40	1	3	1	10	4	1/20/2020	21:21	1	139	86	1	82	2	999	1	36.3	1	2	999	1	99	2	999	2	2	8	1	999	999	1	1	1	999	999	1	3	
72	38	1	3	3	40	1	1/15/2020	15:37	1	126	75	1	92	2	999	2	999	999	2	999	2	999	2	999	2	2	2	1	999	351	2	999	4	21:28	15/01/2020	3	2	
73	18	1	3	3	38	1	1/11/2020	21:47	1	108	70	1	78	2	999	1	36.3	999	2		1	99	2	999	2	2	2	1	999	999	2	999	2	999	999	1	3	2
74	27	1	1	3	39	1	1/9/2020	09:12	1	100	59	1	81	2	999	1	35.2	2	2		1	99	2	999	3	3	2	1	999	999	1	2	2	999	999	1	3	1
75	31	1	3	3	36	1	1/29/2020	15:04	1	112	72	1	84	2	999	1	36.1	1	2	999	1	99	2	999	2	2	7	1	999	999	1	2	1	999	999	1	3	
76	36	2	1	3	34	1	1/5/2020	15:57	1	117	73	1	87	2	999	1	35.8	2	2		1	100	2	999	3	3	7	1	999	145	1	2	1	999	05/01/2020	2	2	
77	21	1	2	3	36	1	1/11/2020	09:35	1	102	57	1	82	2	999	1	36	999	2		1	99	2	999	2	2	7	1	999	145	1	1	1	999	999	2	2	
78	27	1	2	2	14	1	1/5/2020	13:21	1	102	62	1	75	2	999	1	36.9	1	2	999	1	100	2	999	2	2	2	1	999	999	2	2	1	999	05/91/2020	1	3	
79	33	1	4	3	39	1	1/3/2020	22:42	1	120	80	2	999	2	999	1	36.3	2	2		2	999	2	999	3	3	2	1	999	999	1	1	2	999	999	1	3	1
80	27	1	999	2	27	1	1/7/2020	06:01	1	103	63	2	999	2	999	2	999	1	2	999	2	999	2	999	4	4	2	1	999	39	2	1	1	13:10	07/01/2020	2	2	
81	27	1	2	3	30	12	1/30/2020	14:14	1	99	55	1	72	2	999	1	35.6	2	2		1	99	2	999	2	2	7	1	999	999	2	2	1	999	999	1	3	
82	33	1	5	3	34	1	1/5/2020	03:53	1	103	77	1	102	1	20	1	35.9	1	2	999	2	999	2	999	999	0	0	3	N√O S	999	2	3	1	999	999	1	4	
83	33	1	5	3	38	11	1/26/2020	14:02	1	117	62	1	100	2	999	1	37.1	1	2	999	1	99	2	999	1	2	2	2	999	999	2	1	999	999	999	1	3	
84	33	1	5	3	38	4	1/28/2020	17:04	1	111	81	1	113	2	999	1	34.9	1	2	999	1	99	2	999	3	3	7	1	999	999	1	2	2	21:05	28/01/2020	1	3	2
85	39	2	3	1	10	9	1/24/2020	16:30	1	117	71	1	67	2	999	1	36.9	2	2		1	100	2	999	2	2	8	1	999	999	2	1	1	999	999	1	3	
86	39	2	3	1	10	12	1/27/2020	00:47	1	106	61	1	70	2	999	1	36.3	1	2	999	1	99	2	999	1	2	8	2	999	999	1	1	1	999	999	1	3	
87	39	2	3	1	10	1	1/28/2020	01:46	1	90	60	1	70	2	999	2	999	1	2	999	2	999	2	999	3	3	8	1	999	999	1	1	2	999	999	1	3	3
88	29	1	2	3	38	1	1/14/2020	15:45	1	112	72	1	99	2	999	1	36.4	1	2	999	1	99	2	999	3	3	2	1	999	90	2	1	1	999	999	2	2	
89	29	1	2	3	39	1	1/19/2020	22:27	1	116	77	1	88	2	999	2	999	999	2		1	99	2	999	3	3	7	1	999	999	2	1	2	999	999	1	3	2
90	25	1	5	3	38	12	1/29/2020	08:32	1	97	56	1	95	2	999	1	35.4	2	2		1	99	2	999	3	2	2	1	999	999	1	2	2	999	999	1	3	2
91	25	1	5	3	37	12	1/15/2020	17:31	1	107	59	1	75	2	999	2	999	2	2		2	999	2	999	2	3	10	2	999	149	2	2	1	21:30	15/01/2020	2	2	
92	22	1	1	2	23	1	1/3/2020	01:19	1	111	63	1	92	2	999	1	36.2	1	2	999	1	99	2	999	2	2	2	1	999	41	1	1	2	999	03/01/2020	3	1	2
93	29	999	3	3	33	6	1/14/2020	17:49	2	999	999	2	999	2	999	2	999	999	2		2	999	2	999	999	0	0	3	N√O S	999	1	3	999	999	999	1	4	2
94	30	1	2	1	9	1	1/17/2020	22:25	1	103	58	1	67	2	999	1	35.9	2	2		1	98	2	999	2	2	7	1	999	100	1	1	1	999	18/01/2020	3	2	
95	22	1	1	1	9	1	1/27/2020	21:30	1	100	70	1	88	2	999	1	36.4	1	2	999	2	999	2	999	3	3	2	1	999	70	2	1	1	00:40	28/01/2020	2	2	
96	24	1	1	1	9	1	1/17/2020	20:54	1	108	56	1	82	2	999	1	35.9	1	2	999	1	99	2	999	4	3	2	2	999	999	1	1	2	999	999	1	3	3
97	34	1	1	3	36	1	1/18/2020	09:08	1	122	80	1	87	2	999	1	35.6	999	2		1	100	2	999	2	2	7	1	999	999	2	1	2	999	999	1	3	1
98	32	1	4	3	36	1	1/10/2020	10:37	1	131	90	1	90	2	999	1	36.8	1	2	999	1	99	2	999	4	4	3	1	999	128	1	1	2	20:05	10/01/2020	2	2	2
99	25	1	2	3	39	8	1/2/2020	15:38	1	109	69	1	87	2	999	2	999	1	2	999	1	99	2	999	3	3	2	1	999	157	1	999	1	999	02/01/2020	2	2	
100	21	1	2	1	5	1	1/29/2020	19:32	1	117	70	1	121	2	999	1	37	1	2	999	1	99	2	999	2	2	6	1	999	148	2	1	1	999	999	2	2	
101	21	1	2	1	6	1	1/31/2020	13:56	1	118	74	1	95	2	999	1	35.6	1	2	999	1	100	2	999	2	2	3	1	999	64	1	1	1	999	999	2	1	
102	27	1	4	2	15	1	1/7/2020	23:52	1	100	70	1	79	2	999	1	37.1	1	2	999	2	999	2	999	3	3	2	1	999	100	2	2	3	999	999	2	2	
103	29	1	3	3	38	1	1/29/2020	22:07	1	105	70	1	120	2	999	1	35.9	2	2		1	99	2	999	2	2	2	1	999	88	2	2	1	999	999	2	1	
104	27	1	4	3	39	1	1/2/2020	14:40	1	134	89	1	102	2	999	2	999	2	2		1	99	2	999	2	1	2	2	999	100	2	999	1	999	02/01/2020	2	1	
105	27	1	4	3	39	1	1/6/2020	18:41	1	132	81	1	105	2	999	1	36.3	2	2		1	99	2	999	2	1	7	2	999	239	2	999	4	23:15	06/01/2020	3	1	
106	21	1	1	1	5	1	1/18/2020	17:08	1	112	60	1	67	2	999	1	36.7	1	2	999	1	99	2	999	1	2	2	2	999	999	2	999	1	999	999	1	3	
107	21	1	1	1	8	1	1/28/2020	06:35	1	131	78	1	115	2	999	1	36.7	2	2		1	100	2	999	3	3	8	1	999	55	2	999	4	07:40	28/01/2020	3	2	
108	21	1	1	1	8	1	1/29/2020	01:05	1	122	73	1	83	2	999	1	37.1	2	2		1	100	2	999	2	3	8	2	999	999	1	2	1	999	999	1	3	
109	21	1	1	1	8	1	1/29/2020	18:08	1	99	54	1	72	2	999	1	36.3	1	2	999	1	100	2	999	2	2	8	1	999	999	1	1	999	999	999	1	3	
110	32	1	3	3	32	1	1/9/2020	17:14	1	104	54	1	83	2	999	1	34.9	1	2	999	1	100	2	999	3	3	2	1	999	106	2	2	999	999	999	2	1	
111	27	1	1	3	36	2	1/3/2020	08:08	1	107	67	1	71	2	999	1	35.8	1	1	5	1	99	2	999	3	3	2	1	999	999	1	1	2	999	03/01/2020	1	3	1
112	29	1	3	3	40	1	1/28/2020	13:37	1	121	86	1	103	2	999	1	36.8	1	2	999	1	99	2	999	2	2	2	1	999	163	2	2	1	999	999	2	2	
113	29	1	3	3	40	1	1/29/2020	13:08	1	123	87	1	108	2	999	1	35.8	2	2		2	999	2	999	999	0	0	3	N√O S	999	2	3	1	999	999	1	4	
114	29	999	3	3	40	1	1/30/2020	9999	1	131	76	1	115	2	999	2	999	999	2		1	99	2	999	4	4	2	1	999	999	2	1	2	999	999	1	3	2
115	21	1	1	2	27	2	1/17/2020	13:14	1	112	72	1	103	2	999	1	36.3	1	2	999	2	999	2	999	1	2	2	2	999	116	2	2	1	18:40	17/01/2020	2	1	
116	30	1	4	999	999	1	1/16/2020	12:40	1	88	49	1	80	2	999	1	35.8	1	2	999	1	100	2	999	2	2	8	1	999	195	2	999	4	17:30	16/01/2020	3	2	
117	30	1	4	999	999	1	1/26/2020	22:53	1	95	56	1	86	2	999	1	36.1	1	2	999	1	99	2	999	1	2	2	2	999	113	2	999	4	00:50	27/01/2020	3	1	
118	25	1	2	1	7	1	1/26/2020	14:12	1	106	75	1	89	2	999	1	36.3	2	2		1	99	2	999	2	2	8	1	999	93	2	2	4	15:45	26/01/2020	3	1	
119	25	1	2	1	7	1	1/27/2020	06:22	1	103	70	1	80	2	999	1	36.1	1	2	999	1	99	2	999	2	2	8	1	999	148	1	2	1	999	999	2	2	
120	28	1	3	3	32	1	1/2/2020	14:03	1	100	61	1	87	2	999	1	34.9	1	2	999	1	99	2	999	2	2	5	1	999	999	1	999	1	999	02/01/2020	1	3	

121	28	1	3	3	34	3	1/16/2020	17:31	1	110	65	1	83	2	999	1	35.9	1	2	999	1	99	2	999	2	2	3	2	999	109	2	2	1	21:00	16/01/2020	2	1	
122	28	1	3	3	35	3	1/24/2020	14:09	1	120	80	1	95	2	999	2	999	1	2	999	1	99	2	999	3	3	2	1	999	111	2	2	999	999	999	2	2	
123	25	2	2	3	38	1	1/11/2020	23:55	1	110	65	1	90	2	999	1	35.7	999	2		1	99	2	999	2	2	2	1	999	245	2	1	1	999	999	2	2	
124	29	1	1	3	39	1	1/5/2020	16:15	1	133	91	1	107	2	999	1	35	1	1	9	1	98	2	999	4	4	2	1	999	999	1	2	2	999	999	1	3	2
125	29	1	1	3	38	1	1/2/2020	22:54	1	104	72	1	100	2	999	1	36.4	2	2		1	99	2	999	2	2	6	1	999	999	2	1	1	05:50	03/01/2020	1	3	
126	29	1	1	3	39	1	1/5/2020	06:38	1	100	70	2	999	2	999	2	999	1	2	999	2	999	2	999	3	3	2	1	999	112	1	2	1	05/01/	06:38	2	2	
127	33	1	2	3	39	1	1/28/2020	14:59	1	103	68	1	90	2	999	1	36.1	1	2	999	1	99	2	999	2	2	2	1	999	196	2	2	1	999	999	2	2	
128	29	1	3	2	17	3	1/7/2020	08:37	1	110	77	1	75	2	999	1	34.6	2	2		1	100	2	999	3	3	1	1	999	999	2	2	1	999	07/01/2020	1	3	
129	26	1	1	3	41	1	1/17/2020	08:25	1	121	71	1	95	2	999	2	999	2	2		1	99	2	999	2	2	12	1	999	999	1	1	2	999	999	1	3	1
130	18	1	1	3	40	1	1/11/2020	21:25	1	120	72	1	87	2	999	1	36.8	999	2		1	99	2	999	2	2	7	1	999	999	2	999	2	999	999	1	3	2
131	19	3	1	1	9	1	1/21/2020	06:10	1	124	75	1	99	2	999	1	36.4	2	2		1	100	2	999	3	3	12	1	999	999	2	1	999	999	999	1	3	
132	21	1	2	1	11	1	1/24/2020	07:44	1	144	64	1	88	2	999	2	999	1	2	999	1	99	2	999	3	3	2	1	999	46	1	2	1	999	24/01/2020	3	2	
133	25	1	1	3	40	1	1/7/2020	10:20	1	99	67	1	98	2	999	1	35.9	2	2		1	100	2	999	1	2	12	2	999	999	2	1	1	999	07/01/2020	1	3	
134	25	1	1	3	41	1	1/14/2020	01:17	1	106	71	1	83	2	999	2	999	1	2	999	2	999	2	999	3	3	7	1	999	999	1	1	2	999	999	1	3	1
135	29	1	3	2	22	1	1/21/2020	23:38	1	111	62	1	100	2	999	1	36.7	1	2	999	1	99	2	999	1	2	2	2	999	52	2	1	3	06:00	21/01/2020	2	1	
136	24	1	1	3	37	1	1/26/2020	21:15	1	119	79	1	108	2	999	1	37.1	1	2	999	1	99	2	999	2	2	2	1	999	999	1	2	1	999	999	1	3	
137	19	2	1	1	10	1	1/10/2020	10:21	1	129	70	1	88	2	999	1	37	1	2	999	1	99	2	999	2	2	9	1	999	999	1	1	1	999	10/01/2020	1	3	
138	18	999	2	3	35	11	1/29/2020	16:48	1	108	75	1	95	2	999	1	36.6	1	2	999	1	100	2	999	1	2	2	2	999	128	2	2	3	21:00	28/01/2020	2	2	
139	20	2	1	2	13	1	1/8/2020	11:42	1	100	69	1	96	2	999	1	36.54	1	2	999	1	100	2	999	3	3	2	1	999	999	2	2	1	999	08/01/2020	1	3	
140	25	1	3	3	34	1	1/2/2020	12:25	1	102	53	2	999	2	999	2	999	1	2	999	2	999	2	999	2	2	2	1	999	125	2	999	1	999	02/01/2020	2	2	
141	25	1	3	3	35	1	1/9/2020	09:16	1	104	54	1	80	2	999	1	36.4	1	2	999	1	99	2	999	2	3	10	2	999	89	2	2	3	19:30	09/01/2020	2	2	
142	25	1	3	3	39	1	1/13/2020	13:21	1	106	48	1	87	2	999	1	36.4	1	1	5	1	99	2	999	3	3	2	1	999	159	2	2	1	999	999	2	2	
143	25	1	3	3	36	1	1/15/2020	11:01	1	103	57	1	87	2	999	1	36.3	1	2	999	1	99	2	999	1	2	2	2	999	999	1	2	1	999	999	1	3	
144	25	1	3	3	37	1	1/24/2020	15:40	1	107	58	1	78	2	999	1	36.3	1	2	999	1	99	2	999	3	3	2	1	999	65	2	2	1	999	999	2	2	
145	22	1	1	3	37	1	1/5/2020	09:19	1	117	76	1	96	2	999	1	36.4	2	2		1	100	2	999	1	1	12	1	999	111	2	2	1	999	05/01/2020	2	1	
146	22	1	1	3	37	1	1/8/2020	13:01	1	121	92	1	112	2	999	1	35.4	999	2		2	999	2	999	999	0	0	3	N√O S	999	1	3	2	999	08/01/2020	1	4	1
147	22	1	1	3	36	1	1/2/2020	10:36	1	153	102	2	999	2	999	2	999	1	2	999	2	999	2	999	2	3	3	2	999	56	2	999	1	999	02/01/2020	2	2	
148	20	2	1	3	38	1	1/2/2020	21:23	1	113	67	1	74	2	999	1	36.4	2	2		1	99	2	999	2	2	7	1	999	999	1	999	1	999	999	1	3	
149	20	2	1	3	40	1	1/17/2020	18:57	1	110	76	1	78	2	999	1	36.4	2	2		1	99	2	999	2	2	2	1	999	999	2	2	1	999	18/01/2029	1	3	
150	29	1	4	2	24	8	1/20/2020	22:35	1	104	57	1	91	2	999	1	36.1	2	2		1	99	2	999	2	2	6	1	999	999	1	2	1	999	999	1	3	
151	29	1	2	2	20	11	1/26/2020	12:39	1	107	71	1	89	2	999	1	36.2	2	2		2	999	2	999	999	0	0	3	N√O S	999	2	3	1	999	999	1	4	
152	27	1	1	3	40	1	1/5/2020	08:05	1	119	80	1	90	2	999	1	36.4	1	2	999	1	98	2	999	2	2	2	1	999	115	2	2	1	999	05/01/2020	2	1	
153	27	1	1	3	41	8	1/13/2020	999	1	132	87	1	92	2	990	1	34.5	1	2	999	1	99	2	999	2	2	12	1	999	999	1	1	2	999	999	1	3	2
154	27	1	1	3	40	3	1/10/2020	10:54	1	123	86	1	95	2	999	1	36.8	1	2	999	1	99	2	999	2	2	10	1	999	999	2	999	4	999	999	1	3	
155	26	1	3	3	39	1	1/20/2020	10:18	1	127	68	1	75	2	999	1	36.5	1	2	999	1	99	2	999	3	3	2	1	999	144	1	2	1	999	999	2	2	
156	26	1	3	3	39	1	1/21/2020	06:25	1	111	61	1	84	2	999	1	36.1	2	2		1	99	2	999	3	3	7	1	999	15	2	1	2	09:20	21/01/2020	2	1	1
157	21	3	2	1	8	1	1/8/2020	10:33	2	999	999	2	999	2	999	2	999	1	2	999	2	999	2	999	2	2	2	1	999	999	2	2	1	999	08/01/2020	1	3	
158	27	1	2	2	20	1	1/27/2020	09:43	1	99	50	1	72	2	999	1	35.9	1	2	999	1	99	2	999	1	2	2	2	999	197	1	1	1	15:45	27/01/2020	2	2	
159	32	1	2	3	34	2	1/17/2020	09:46	1	112	68	1	98	2	999	1	36	1	2	999	1	99	2	999	2	2	9	1	999	74	2	1	1	14:20	17/01/2020	2	1	
160	19	999	1	3	36	2	1/19/2020	999	1	100	63	1	93	2	999	1	36.1	999	2		1	99	2	999	1	1	12	1	999	999	2	2	2	999	19/01/2020	1	3	1
161	23	2	1	3	34	1	1/3/2020	15:09	1	103	70	1	123	2	999	1	36.3	1	1	3	1	99	2	999	2	2	2	1	999	257	2	1	1	21:15	03/01/2020	2	2	
162	20	1	1	3	41	1	1/3/2020	05:43	1	105	61	1	87	2	999	1	35.8	2	2		1	99	2	999	2	2	7	1	999	999	1	2	2	999	03/01/2020	1	3	2
163	25	2	2	3	39	12	1/29/2020	16:20	1	103	57	1	76	2	999	1	36.2	2	2		1	99	2	999	2	2	7	2	999	145	2	2	1	999	999	2	2	
164	30	2	3	1	5	11	1/10/2020	09:21	1	109	66	1	90	2	999	1	36.8	1	2	999	1	90	2	999	2	2	9	1	999	99	1	1	3	15:40	10/01/2020	2	1	
165	30	2	3	1	5	1	1/15/2020	11:51	1	117	65	1	68	2	999	1	36.7	1	2	999	1	100	2	999	2	2	2	1	999	309	2	999	4	19:00	15/01/2020	3	2	
166	30	2	3	1	5	1	1/18/2020	16:59	1	109	64	1	79	2	999	1	36.8	1	2	999	1	100	2	999	2	2	8	1	999	999	2	999	1	999	999	1	3	
167	34	1	2	3	39	11	1/17/2020	16:24	1	123	72	1	93	2	999	1	35.3	2	2		1	99	2	999	2	3	7	2	999	80	2	2	1	999	999	2	2	
168	34	1	2	3	40	1	1/24/2020	09:22	1	105	65	1	84	2	999	1	36.2	2	2		1	99	2	999	1	2	12	2	999	308	1	2	1	999	999	2	2	
169	34	1	2	3	40	1	1/28/2020	10:31	1	118	76	1	81	2	999	1	34	999	2		1	99	2	999	2	2	7	1	999	999	2	1	2	999	999	1	3	2
170	30	2	1	2	13	1	1/29/2020	16:54	1	98	49	1	70	2	999	1	35.2	1	2	999	1	100	2	999	3	2	3	2	999	111	1	2	1	999	999	2	1	
171	30	1	2	3	40	1	1/16/2020	08:03	1	105	72	1	93	2	999	1	36.7	999	2		1	100	2	999	2	2	2	1	999	327	2	2	2	999	15/01/2020	2	2	1
172	22	1	1	1	5	1	1/2/2020	12:45	2	999	999	2	999	2	999	2	999	2	2		2	999	2	999	1	1	2	1	999	999	2	999	1	999	02/01/2020	1	3	
173	29	1	1	1	6	1	1/24/2020	01:28	1	124	56	1	98	2	999	1	37.3	1	2	999	1	99	2	999	2	2	2	1	999	999	2	2	1	999	999	1	3	
174	29	1	1	1	6	1	1/27/2020	08:31	1	111	59	1	94	2	999	1	36.8	1	2	999	1	100	2	999	1	2	2	2	999	999	2	2	1	999	999	1	3	
175	22	1	3	1	8	1	1/11/2020	23:32	1	122	78	1	106	2	999	1	36	1	2	999	1	98	2	999	2	2	8	1	999	999	2	1	999	999	999	1	3	
176	28	2	4	2	13	1	1/20/2020	12:25	1	117	75	1	98	2	999	1	36.6	1	2	999	1	99	2	999	4	3	2	2	999	999	1	2	1	999	999	1	3	
177	22	1	3	3	36	1	1/3/2020	16:06	1	138	76	1	84	2	999	1	37	2	2		1	99	2	999	1	2	10	2	999	166	2	999	4	20:45	03/01/2020	3	2	
178	26	1	1	2	26	1	1/2/2020	09:35	2	999	999	2	999	2	999	2	999	2	2		2	999	2	999	3	2	10	2	999	999	2	999	1	999	02/01/2020	1	3	
179	26	1	1	3	40	1	1/26/2020	19:54	1	117	77	1	89	2	999	1	35.9	2	2		1	99	2	999	3	3	7	1	999	106	2	2	1	01:00	27/01/2020	2	2	
180	26	1	1	3	39	1	1/26/2020	10:42	1	129	79	1	90	2	999	1	36.3	1	2	999	1	99	2	999	3	3	7	1	999	78	2	2	1	999	999	2	2	
181	25	3	2	3	36	2	1/15/2020	06:01	1	106	69	1	78	2	999	1	35.9	1	2	999	1	99	2	999	2	2	2	1	999	259	2	2	3	17:15	15/01/2020	2	2	
182	27	1	1	3	33	1	1/13/2020	19:14	1	110	70	1	73	2	999	2	999	1	2	999	2	999	2	999	2	2	7	1	999	999	1	2	1	999	999	1	3	
183	30	1	1	2	19	12	1/3/2020	14:33	1	112	13	1	121	2	999	2	999	1	1	10	1	100	2	999	4	4	2	1	999	127	2	1	2	20:30	03/01/2020	2	2	4
184	30	1	1	2	18	12	1/11/2020	01:13	1	100	70	1	83	2	999	2	999	1	2	999	2	999	2	999	4	4	2	1	999	97	1	1	1	08:15	11/01/2020	2	2	
185	18	2	1	3	41	1	1/15/2020	13:47	1	107	72	1	84	2	999	2	999	999	2		2	999	2	999	3	2	2	2	999	353	2	999	4	19:40	15/01/2020	3	2	
186	30	1	2	3	41	1	1/21/2020	22:55	1	115	55	1	66	2	999	1	36	1	2	999	1	99	2	999	2	2	2	1	999	999	1	1	1	999	999	1	3	
187	30	1	2	3	41	1	1/22/2020	12:19	1	104	59	1	67	2	999	1	36.3	2	2		1	100	2	999	2	2	12	1	999	999	1	1	2	999	999	1	3	2
188	19	1	1	3	35	1	1/18/2020	20:31	1	112	62	1	78	2	999	2	999	1	2	999	2	999	2	999	2	2	2	1	999	199	2	1	999	999	999	2	2	

189	19	1	1	3	35	12	1/23/2020	09:50	1	98	64	1	106	2	999	1	35.5	1	2	999	1	100	2	999	2	2	2	1	999	999	1	1	1	999	999	1	3	
190	22	2	2	3	39	1	1/26/2020	01:17	1	140	100	2	999	2	999	2	999	1	2		2	999	2	999	3	3	2	1	999	3	1	1	1	999	999	2	1	
191	40	1	2	3	29	2	1/3/2020	05:59	1	98	52	1	72	2	999	1	35.6	2	2		1	99	1	105	1	1	12	1	999	151	2	1	2	10:00	03/01/2020	2	1	4
192	21	1	1	3	40	1	1/4/2020	15:09	1	128	76	1	100	2	999	1	35.4	2	2		1	99	2	999	2	2	7	1	999	102	1	1	1	999	04/01/2020	2	1	
193	29	999	3	3	38	7	1/15/2020	03:19	1	90	60	1	95	2	999	1	36.2	999	2		2	999	2	999	999	0	0	3	N√O S	999	1	3	2	999	999	1	4	2
194	31	1	1	2	25	1	1/29/2020	14:54	1	105	62	1	97	2	999	1	36.3	1	2	999	1	100	2	999	2	2	2	1	999	999	1	2	1	999	999	1	3	
195	23	1	4	2	22	1	1/13/2020	12:56	1	91	55	1	91	2	999	1	35.9	999	2		1	99	2	999	3	3	2	1	999	999	2	1	1	18:30	13/01/2020	1	3	
196	27	3	1	3	33	1	1/21/2020	12:59	1	112	62	1	103	2	999	1	35.9	2	2		1	99	2	999	2	2	8	1	999	46	1	1	1	17:16	21/01/2020	2	1	
197	27	3	1	3	33	1	1/23/2020	16:20	1	110	59	1	89	2	999	1	35.9	2	2		1	99	2	999	2	2	7	1	999	999	2	2	1	999	999	1	3	
198	20	1	1	2	22	1	1/12/2020	08:29	1	101	72	1	72	2	999	1	37.3	1	2	999	2	999	2	999	4	4	2	1	999	76	2	1	2	999	999	2	2	999
199	35	1	2	3	30	2	1/13/2020	07:23	1	116	69	1	80	2	999	1	35.6	2	2		1	99	1	84	1	1	12	1	999	7	1	2	1	18:00	13/01/2020	2	1	
200	35	1	2	3	31	12	1/15/2020	08:54	1	136	71	1	84	2	999	1	36.1	999	2		1	99	2	999	2	2	8	1	999	999	2	2	1	999	15/01/2020	1	3	
201	35	1	2	3	31	12	1/16/2020	03:44	1	124	73	1	78	2	999	1	36.4	999	2		2	999	2	999	999	0	0	3	N√O S	36	2	3	999	999	999	2	4	1
202	34	1	1	3	28	1	1/5/2020	15:28	1	134	83	1	88	2	999	1	36.7	1	1	8	1	100	2	999	4	4	2	1	999	62	2	1	1	20:15	05/01/2020	2	2	
203	32	1	4	2	16	1	1/3/2020	11:38	1	107	60	1	85	2	999	1	36.7	2	2		2	999	2	999	2	2	6	1	999	999	2	2	1	999	03/01/2020	1	3	
204	28	2	3	999	999	1	1/22/2020	14:44	1	138	81	1	79	2	999	1	37	1	2	999	1	100	2	999	1	2	2	2	999	354	1	2	1	999	999	2	2	
205	35	1	1	2	22	1	1/16/2020	20:50	1	90	60	1	75	2	999	2	999	1	2	999	2	999	2	999	1	2	2	2	999	999	2	2	1	999	999	1	3	
206	24	1	4	1	7	1	1/30/2020	07:31	1	115	64	1	91	2	999	2	999	2	2		1	99	2	999	1	3	12	2	999	999	2	2	1	999	999	1	3	
207	24	1	4	1	6	1	1/27/2020	07:03	1	97	61	1	86	2	999	1	36.4	1	2	999	1	99	2	999	1	2	2	2	999	999	2	2	1	999	999	1	3	
208	24	1	4	1	6	1	1/26/2020	00:49	1	120	80	2	999	2	999	2	999	1	2	999	2	999	2	999	3	3	2	1	999	999	2	2	1	999	999	1	3	
209	28	1	2	3	37	1	1/12/2020	02:00	1	98	68	1	84	2	999	1	35.4	999	2		1	99	2	999	3	3	2	1	999	115	2	1	1	999	999	2	2	
210	21	2	1	3	40	1	1/24/2020	14:59	1	114	68	1	77	2	999	2	999	2	2		1	99	2	999	1	2	12	2	999	141	2	2	1	999	999	2	2	
211	21	2	1	3	40	1	1/26/2020	23:09	1	120	66	1	81	2	999	1	36.7	999	2		1	99	2	999	2	2	2	1	999	131	2	1	1	999	999	2	2	
212	31	1	2	1	7	1	1/13/2020	20:04	1	124	64	1	84	2	999	1	36	1	2	999	2	999	2	999	2	2	6	1	999	86	1	1	1	999	999	2	1	
213	23	1	5	1	9	3	1/24/2020	00:12	1	121	57	1	106	2	999	1	37.4	1	2	999	1	99	2	999	1	2	2	2	999	131	2	1	1	05:30	24/01/2020	2	2	
214	23	1	5	1	10	1	1/24/2020	17:24	1	144	100	1	89	2	999	1	36.6	1	2	999	1	99	2	999	4	4	2	1	999	46	2	1	1	999	999	2	2	
215	23	1	5	1	9	1	1/27/2020	19:05	1	138	79	1	99	2	999	1	37.3	1	2	999	2	999	2	999	1	2	2	2	999	90	1	1	1	00:15	28/01/2020	2	1	
216	23	1	5	1	10	1	1/29/2020	07:09	1	133	67	1	91	2	999	1	35.6	1	2	999	1	100	2	999	1	2	2	2	999	171	2	2	1	11:35	29/01/2020	2	2	
217	23	1	4	1	10	3	1/30/2020	12:00	1	129	70	1	99	2	999	1	36.6	1	2	999	1	99	2	999	2	2	8	1	999	999	1	1	1	999	999	1	3	
218	40	2	2	3	40	11	1/15/2020	20:52	1	129	79	1	83	2	999	1	36.7	1	2	999	1	99	2	999	2	2	9	1	999	128	2	1	1	999	999	2	2	
219	24	1	2	3	40	1	1/5/2020	14:54	1	103	67	1	84	2	999	1	35.6	2	2		1	100	2	999	2	2	12	1	999	999	1	2	2	999	05/01/2020	1	3	1
220	23	1	1	3	38	1	1/27/2020	09:47	1	122	68	1	104	2	999	1	35.3	2	2		1	99	2	999	2	2	7	1	999	999	1	1	1	999	999	1	3	
221	33	1	2	2	20	1	1/29/2020	21:06	1	95	61	1	73	2	999	1	36.2	1	2	999	1	99	2	999	1	2	4	2	999	999	1	2	1	00:30	30/01/2020	1	3	
222	29	1	2	3	38	2	1/6/2020	14:35	1	118	73	1	94	2	999	1	37	2	2		1	99	2	999	3	3	2	1	999	175	2	2	1	999	06/01/2020	2	2	
223	43	1	3	2	14	2	1/16/2020	12:33	1	115	74	1	106	2	999	1	36.1	1	2	999	1	99	2	999	1	2	2	2	999	999	2	2	1	999	16/01/2020	1	3	
224	26	1	1	1	7	1	1/25/2020	21:12	1	124	75	1	67	2	999	1	35.9	2	2		2	999	2	999	2	2	9	1	999	109	1	1	1	999	999	2	1	
225	29	1	1	3	40	3	1/7/2020	18:29	1	147	89	1	85	2	999	1	36.7	1	1	6	1	99	2	999	3	3	12	1	999	209	2	2	3	23:30	07/01/2020	2	2	
226	22	999	2	3	39	999	1/15/2020	04:13	1	105	72	1	86	2	999	1	35.8	999	2		2	999	2	999	999	0	0	3	N√O S	999	1	3	2	999	999	1	4	2
227	29	1	2	3	38	1	1/28/2020	19:01	1	119	79	1	88	2	999	1	36.9	2	2		1	100	2	999	3	3	8	1	999	999	2	1	2	999	999	1	3	1
228	21	1	1	3	40	1	1/6/2020	03:35	1	100	60	1	103	2	999	1	37	1	2	999	1	99	2	999	999	0	0	3	N√O S	999	1	3	2	999	06/01/2020	1	4	2
229	32	1	1	3	30	1	1/6/2020	23:33	1	107	66	1	100	2	999	2	999	1	2	999	1	99	2	999	4	4	2	1	999	53	2	2	1	999	06/01/2020	2	2	
230	21	1	1	3	42	1	1/27/2020	18:46	1	129	89	1	108	2	999	1	35.4	2	2		1	99	2	999	2	2	12	1	999	999	1	1	2	999	999	1	3	1
231	28	1	1	2	20	1	1/28/2020	20:56	1	115	54	1	89	2	999	1	35.2	1	2	999	1	99	2	999	4	3	2	1	999	79	2	1	1	999	999	2	2	
232	31	1	999	3	29	1	1/8/2020	19:04	1	100	80	1	100	2	999	1	36.7	999	2		2	999	2	999	999	0	0	3	N√O S	999	2	3	2	999	08/01/2020	1	4	2
233	25	2	3	3	40	1	1/15/2020	21:25	1	115	71	1	90	2	999	1	36.3	2	2		1	99	2	999	3	3	10	1	999	75	2	2	1	999	999	2	2	
234	25	2	3	3	40	1	1/17/2020	06:32	2	999	999	2	999	2	999	2	999	1	2	999	2	999	2	999	3	3	2	1	999	999	2	1	2	999	999	1	3	2
235	21	1	2	2	16	1	1/11/2020	17:01	1	115	72	1	97	2	999	1	35.9	1	2	999	1	100	2	999	2	2	6	1	999	269	2	1	3	999	999	2	2	
236	21	1	2	2	18	1	1/21/2020	09:37	1	130	80	1	107	2	999	1	36.4	1	2	999	1	99	2	999	3	3	1	1	999	83	2	1	1	999	999	2	2	
237	21	1	2	2	18	1	1/26/2020	09:59	1	129	87	1	102	2	999	1	35.4	1	2	999	1	99	2	999	3	3	6	1	999	61	1	1	1	999	999	2	2	
238	21	1	2	2	19	12	1/29/2020	12:22	1	92	48	1	102	2	999	1	36.7	1	2	999	1	99	2	999	2	2	6	1	999	999	1	2	1	999	999	1	3	
239	21	1	2	2	19	12	1/31/2020	19:15	1	143	83	1	103	2	999	2	999	1	2	999	2	999	2	999	3	3	2	1	999	15	1	1	2	999	999	2	1	4
240	37	1	4	2	27	12	1/27/2020	16:21	1	111	63	1	107	2	999	1	35.9	1	2	999	1	99	2	999	3	3	7	1	999	69	1	2	1	18:40	27/01/2020	2	2	
241	19	1	1	3	40	1	1/26/2020	22:46	1	121	67	1	114	2	999	1	37.1	999	2		1	99	2	999	3	3	7	1	999	54	2	1	999	999	999	2	2	
242	19	999	1	3	40	1	1/26/2020	16:50	1	121	64	1	110	2	999	1	37.2	999	2		1	99	2	999	3	3	2	1	999	75	2	2	1	999	999	2	2	
243	20	1	1	3	39	1	1/29/2020	16:59	1	136	76	1	111	2	999	1	36.4	1	2	999	1	100	2	999	1	2	2	2	999	131	1	2	1	999	999	2	2	
244	33	1	3	3	40	1	1/4/2020	18:16	1	112	57	1	91	2	999	1	36.6	2	2		1	98	2	999	2	3	7	2	999	145	2	2	999	999	04/01/2020	2	2	
245	33	1	3	3	39	1	1/6/2020	07:04	1	115	71	1	95	2	999	1	35	1	2	999	1	99	2	999	3	3	2	1	999	999	1	1	2	999	06/01/2020	1	3	1
246	21	1	1	1	12	1	1/2/2020	15:47	1	100	63	1	94	2	999	2	999	2	2		1	99	2	999	2	2	6	1	999	93	1	999	1	20:45	02/01/2020	2	1	
247	21	2	2	3	35	1	1/29/2020	15:42	1	104	60	1	81	2	999	1	35.2	2	2		1	99	2	999	3	3	7	1	999	999	1	2	1	999	999	1	3	
248	40	1	4	3	36	1	1/6/2020	17:00	1	127	70	1	107	2	999	1	36.3	1	2	999	1	99	2	999	3	3	2	1	999	165	2	2	1	999	06/01/2020	2	2	
249	40	1	4	3	38	1	1/19/2020	04:07	1	139	87	1	108	2	999	1	36.8	2	2		2	999	2	999	999	0	0	3	N√O S	999	1	3	2	08:10	19/01/2020	1	4	2
250	23	1	3	2	26	7	1/14/2020	19:34	1	113	63	1	100	2	999	1	36.1	1	2	999	1	99	2	999	1	2	2	2	999	116	1	2	1	999	999	2	1	
251	34	3	1	3	38	12	1/8/2020	07:111	1	113	53	1	72	2	999	1	35.8	2	2	999	1	98	2	999	2	2	12	1	999	999	1	1	2	999	08/01/2020	1	3	1
252	27	1	3	3	40	1	1/22/2020	15:28	1	134	81	1	70	2	999	1	35.9	999	2		1	99	2	999	2	2	8	1	999	999	2	2	2	999	999	1	3	2
253	23	2	3	1	8	1	1/7/2020	20:54	1	120	70	1	85	2	999	1	35.9	1	2	999	2	999	2	999	3	3	2	1	999	999	1	1	1	999	999	1	3	
254	27	1	2	3	37	1	1/20/2020	06:04	1	117	57	1	108	2	999	1	36.3	999	2		1	99	2	999	3	3	7	1	999	999	1	1	2	999	999	1	3	1
255	20	1	2	3	37	1	1/2/2020	15:43	1	116	57	1	100	2	999	2	999	1	1	8	1	99	2	999	4	4	2	1	999	52	2	2	3	20:47	02/01/2020	2	2	
256	20	1	2	3	38	1	1/7/2020	16:47	1	110	73	1	112	2	999	1	36.7	999	2		1	99	2	999	2	2	7	1	999	999	1	2	1	00:00	08/01/2020	1	3	

257	20	1	2	3	39	1	1/12/2020	21:01	1	106	52	1	92	2	999	1	36.8	1	2	999	1	99	2	999	3	2	3	2	999	109	2	2	999	999	999	2	1	
258	20	1	2	3	40	1	1/19/2020	12:53	1	110	72	1	110	2	999	1	36.2	1	2	999	1	99	2	999	2	2	7	1	999	157	2	2	2	999	999	2	1	2
259	27	2	1	2	20	1	1/20/2020	21:50	1	113	58	1	92	2	999	1	36.7	1	2	999	1	99	2	999	1	2	2	2	999	122	2	2	3	01:00	21/01/2020	2	2	
260	22	2	2	3	38	1	1/22/2020	13:41	1	112	62	1	90	2	999	1	36.6	2	2		1	99	2	999	2	2	7	1	999	129	2	2	1	999	999	2	2	
261	20	3	1	3	40	8	1/25/2020	12:46	1	124	71	1	81	2	999	1	36.8	1	2	999	1	99	2	999	1	2	2	2	999	143	1	1	1	999	999	2	2	
262	20	2	1	3	40	8	1/26/2020	07:38	1	127	61	1	79	2	999	1	36.2	2	2		1	99	2	999	3	3	7	1	999	118	2	2	1	11:00	26/01/2020	2	2	
263	33	1	2	1	6	1	1/16/2020	12:04	1	92	64	1	81	2	999	1	36.3	1	2	999	1	100	2	999	1	2	2	2	999	206	2	999	1	999	999	2	2	
264	30	2	3	2	26	12	1/30/2020	06:22	1	120	79	1	96	2	999	1	35	1	2	999	1	99	2	999	4	3	2	2	999	999	1	2	1	110:9	30/01/2020	1	3	
265	23	1	1	2	16	1	1/5/2020	10:05	1	93	50	1	77	2	999	1	36.4	1	2	999	1	99	2	999	2	2	2	1	999	85	2	1	1	999	05/01/2020	2	1	
266	23	1	1	1	8	1	1/6/2020	03:57	1	60	40	1	75	2	999	1	36	1	2	999	1	99	2	999	999	0	0	3	N√O S	37	1	3	999	999	06/01/2020	2	4	
267	32	2	2	1	12	1	1/28/2020	15:38	1	107	51	1	60	2	999	1	36.7	1	2	999	1	99	2	999	2	2	8	1	999	82	2	2	1	999	999	2	2	
268	31	1	3	3	40	1	1/23/2020	11:22	1	110	70	1	91	2	999	1	36.4	1	2	999	1	100	2	999	2	2	2	1	999	178	2	2	1	999	999	2	2	
269	31	1	3	3	40	1	1/27/2020	12:46	1	131	91	1	93	2	999	1	36.3	2	2		2	999	2	999	999	0	0	3	N√O S	999	1	3	2	999	999	1	4	1
270	44	1	4	3	31	3	1/8/2020	15:41	1	131	51	1	91	2	999	2	999	1	2	999	1	98	2	999	3	4	3	2	999	139	1	2	1	20:00	08/01/2020	2	2	
271	44	1	4	3	34	3	1/30/2020	15:33	1	144	69	1	101	2	999	1	36.3	1	2		1	99	2	999	4	4	3	1	999	30	2	1	1	999	999	3	2	
272	33	1	1	3	39	1	1/6/2020	19:27	1	143	79	2	999	2	999	2	999	1	1	8	2	999	2	999	4	4	2	1	999	999	1	2	1	999	999	1	3	
273	34	999	1	3	40	1	1/13/2020	999	1	122	8	1	77	2	999	1	35.6	999	2		1	99	2	999	1	1	12	1	999	999	2	2	2	999	999	1	3	1
274	20	1	2	3	38	1	1/30/2020	15:47	1	108	70	1	80	2	999	1	36.6	2	2		1	100	2	999	3	3	2	1	999	113	1	1	2	19:45	30/01/2020	2	2	2
275	26	1	3	1	10	1	1/12/2020	22:11	1	94	53	1	71	2	999	1	36.7	1	2	999	1	99	2	999	2	2	8	1	999	46	1	1	1	00:50	13/01/2020	2	3	
276	21	2	2	2	26	1	1/27/2020	16:06	1	105	69	1	91	2	999	1	34.5	1	2	999	1	99	2	999	3	3	7	1	999	999	1	2	1	999	999	1	3	
277	34	1	1	3	36	3	1/6/2020	11:47	1	140	90	1	96	2	999	1	36.7	2	2		1	99	2	999	3	3	10	1	999	163	2	2	1	999	05/01/2020	2	2	
278	34	1	1	3	39	1	1/23/2020	06:13	1	110	80	2	999	1	20	1	36	1	2	999	2	999	2	999	3	3	2	1	999	47	2	2	2	10:30	23/01/2020	2	2	1
279	25	1	3	2	24	1	1/12/2020	18:30	1	88	62	1	112	2	999	1	37	999	2		1	99	2	999	1	2	6	2	999	999	1	2	2	999	999	1	3	4
280	29	2	2	3	39	3	1/6/2020	09:11	1	132	83	1	95	2	999	2	999	1	2	999	1	99	2	999	4	4	12	1	999	19	1	1	2	999	06/01/2020	2	2	1
281	37	1	3	999	999	1	1/20/2020	19:51	1	131	82	1	94	2	999	1	36.3	1	2	999	1	99	2	999	1	2	2	2	999	999	1	2	1	999	999	1	3	
282	33	3	2	1	7	1	1/30/2020	00:23	1	100	56	1	83	2	999	1	36.7	2	2		1	100	2	999	3	3	8	1	999	57	1	1	2	999	999	2	2	3
283	22	1	1	2	16	1	1/3/2020	22:06	1	93	54	2	999	2	999	1	37	1	2	999	2	999	2	999	4	4	2	1	999	999	2	2	1	999	999	1	3	
284	30	1	1	3	40	11	1/15/2020	12:57	1	136	84	1	102	2	999	2	999	1	2	999	2	999	2	999	3	3	2	1	999	999	2	2	2	999	999	1	3	1
285	23	2	1	3	39	1	1/22/2020	15:54	1	127	79	1	92	2	999	1	36.8	2	2		1	99	2	999	3	3	7	1	999	999	1	1	2	999	999	1	3	2
286	26	1	4	2	15	1	1/25/2020	15:02	1	103	61	1	99	2	999	1	35.9	2	2		1	99	2	999	2	2	8	1	999	999	1	1	1	999	999	1	3	
287	36	1	1	3	38	1	1/9/2020	11:52	2	999	999	2	999	2	999	2	999	999	2		2	999	2	999	3	4	2	2	999	58	2	2	1	999	999	2	2	
288	36	1	1	3	39	1	1/11/2020	00:26	1	120	80	1	90	2	999	2	999	999	2		2	999	2	999	3	3	2	1	999	999	1	1	2	999	999	1	3	1
289	41	1	2	1	9	1	1/5/2020	09:37	1	106	66	1	84	2	999	1	36.4	2	2		1	100	2	999	2	2	8	1	999	999	2	1	1	999	05/01/2020	1	3	
290	41	1	2	1	9	1	1/6/2020	05:52	1	118	65	1	79	2	999	1	36.2	2	2		1	100	2	999	3	3	8	1	999	288	2	1	1	999	06/01/2020	2	2	
291	30	1	999	999	999	1	1/8/2020	00:17	2	999	999	2	999	2	999	1	36.7	1	2	999	2	999	2	999	3	3	2	1	999	999	2	2	1	999	08/01/2020	1	3	
292	20	1	1	2	19	1	1/15/2020	11:38	1	118	66	1	66	2	999	1	35.8	2	2		1	99	2	999	1	2	12	2	999	999	2	2	999	999	999	1	3	
293	25	1	4	3	31	1	1/6/2020	22:27	1	109	71	1	106	2	999	2	999	2	2		1	99	2	999	2	2	8	1	999	999	1	1	1	999	999	1	3	
294	26	2	3	999	999	1	1/6/2020	14:22	1	104	69	1	84	2	999	1	36.1	1	2	999	1	100	2	999	3	3	8	1	999	144	2	2	1	999	07/01/2020	2	2	
295	31	1	4	3	35	7	1/9/2020	13:11	1	101	57	1	84	2	999	1	35.9	999	2		1	100	2	999	2	2	7	1	999	999	2	1	2	999	999	1	3	1
296	38	1	3	3	32	2	1/24/2020	07:20	1	120	90	2	999	2	999	1	36.4	2	2		1	99	2	999	1	2	12	2	999	10	2	2	2	18:00	24/01/2020	2	1	4
297	38	1	3	3	32	3	1/31/2020	21:02	1	122	56	1	78	2	999	2	999	1	2	999	2	999	2	999	1	2	2	2	999	118	2	1	1	01:00	01/02/2020	2	1	
298	40	1	4	1	9	12	1/29/2020	02:22	1	131	88	1	77	2	999	1	36.1	1	2	999	2	999	2	999	999	0	0	3	N√O S	358	1	3	1	09:37	29/01/2020	2	4	
299	40	1	4	1	8	1	1/22/2020	15:48	1	126	57	1	90	2	999	1	35.9	1	2	999	1	100	2	999	1	2	3	2	999	999	2	2	1	23:30	22/01/2020	1	3	
300	29	1	1	2	18	4	1/19/2020	22:19	1	97	65	1	96	2	999	1	36.7	1	2	999	1	99	2	999	3	3	6	1	999	176	2	1	999	999	999	2	2	
301	29	1	1	2	19	12	1/29/2020	04:35	1	110	64	1	94	2	999	1	36.1	2	2		2	999	2	999	999	0	0	3	N√O S	999	1	3	1	999	999	1	4	
302	33	3	2	2	21	1	1/21/2020	00:47	1	109	81	1	110	2	999	1	35	1	2	999	1	99	1	112	3	3	6	1	999	999	2	1	1	999	21/01/2020	1	3	
303	25	1	2	3	38	1	1/11/2020	08:13	1	77	66	2	999	2	999	2	999	999	2		2	999	2	999	3	3	7	1	999	999	2	1	2	999	11/01/2020	1	3	2
304	29	1	1	3	37	1	1/26/2020	06:35	1	110	80	2	999	2	999	2	999	2	2		2	999	2	999	3	3	7	1	999	999	2	1	2	999	999	1	3	1
305	20	1	1	2	16	1	1/15/2020	15:06	1	80	50	1	60	2	999	2	999	1	2	999	2	999	2	999	3	2	2	2	999	999	2	999	4	999	999	1	3	
306	38	2	2	1	11	3	1/27/2020	10:55	1	131	71	1	82	2	999	1	35.2	2	2		1	100	2	999	2	2	8	1	999	999	2	1	1	999	999	1	3	
307	22	1	999	999	999	1	1/22/2020	13:53	1	116	72	1	85	2	999	1	36.7	2	2		1	98	2	999	3	3	12	1	999	127	2	1	4	16:40	22/01/2020	3	2	
308	30	1	3	3	31	1	1/18/2020	19:04	1	115	62	1	80	2	999	1	36.6	1	2	999	2	999	2	999	2	2	2	1	999	999	2	2	999	999	999	1	3	
309	30	1	3	3	32	1	1/21/2020	16:48	1	102	63	1	97	2	999	1	36	2	2		1	99	2	999	2	2	7	1	999	999	2	2	1	999	999	1	3	
310	26	1	3	1	6	1	1/3/2020	22:29	1	110	70	1	80	2	999	2	999	1	2	999	2	999	2	999	4	4	2	1	999	40	1	1	2	00:40	04/01/2020	3	2	3
311	30	1	6	3	36	6	1/22/2020	11:37	2	999	999	2	999	2	999	2	999	999	2	999	2	999	2	999	999	0	0	3	N√O S	23	1	3	1	999	22/01/2020	2	4	
312	18	999	1	3	37	2	1/14/2020	14:13	1	99	60	1	101	2	999	1	36.8	2	2		1	99	2	999	1	2	12	2	999	999	2	2	999	999	999	1	3	
313	18	999	1	3	38	2	1/20/2020	07:29	1	100	64	1	85	2	999	1	36.1	2	2		1	99	1	71	1	1	12	1	999	999	1	2	1	999	20/01/2020	1	3	
314	18	1	1	3	36	2	1/6/2020	06:18	1	101	64	1	103	2	999	1	35.8	2	2		1	99	2	999	1	1	12	1	999	72	1	1	1	19:08	06/01/2020	2	1	
315	18	1	1	3	40	1	1/27/2020	22:59	1	104	60	1	70	2	999	2	999	999	2		2	999	2	999	2	2	2	1	999	100	1	2	1	999	999	2	1	
316	18	999	1	3	40	2	1/30/2020	11:40	1	115	50	1	100	2	999	1	36.5	2	2		1	99	2	999	1	2	12	2	999	999	1	2	2	999	999	1	3	1
317	35	1	4	3	36	1	1/7/2020	17:08	1	98	60	1	75	2	999	1	35.9	2	2		1	99	2	999	1	1	12	1	999	999	2	999	4	999	999	1	3	
318	27	1	3	3	39	1	1/7/2020	01:39	1	120	65	1	90	2	999	2	999	1	2	999	1	98	2	999	4	4	2	1	999	51	1	2	1	999	07/01/2020	2	2	
319	27	1	3	3	39	1	1/6/2020	14:23	1	107	60	1	91	2	999	1	36.7	1	1	4	1	99	2	999	3	3	2	1	999	60	2	2	1	999	06/01/2020	2	2	
320	27	1	3	3	39	2	1/8/2020	09:09	1	100	60	1	102	2	999	1	36.3	1	2	999	1	99	2	999	3	3	2	1	999	101	1	2	1	999	08/01/2020	2	2	
321	27	1	3	3	40	2	1/9/2020	08:36	1	113	60	1	84	2	999	1	35.9	999	2		1	99	2	999	3	3	2	1	999	999	2	2	2	999	09/01/2020	1	3	2
322	19	1	2	2	16	1	1/11/2020	20:11	1	119	71	1	75	2	999	1	36.1	1	2	999	1	99	2	999	2	2	5	1	999	999	1	2	1	999	999	1	3	
323	19	1	2	1	6	12	1/31/2020	16:47	1	96	57	1	90	2	999	1	36.4	2	2		1	100	2	999	2	2	6	1	999	123	2	1	1	23:00	31/01/2020	2	2	
324	19	1	1	2	17	1	1/5/2020	17:31	1	102	58	1	85	2	999	1	36.8	1	2	999	1	99	2	999	2	2	2	1	999	119	2	2	1	22:30	05/01/2020	2	1	

325	20	2	3	3	41	1	1/14/2020	22:12	1	113	75	1	100	2	999	1	35.6	2	2		1	99	2	999	2	2	12	1	999	999	1	1	2	999	999	1	3	2
326	22	1	2	3	40	1	1/2/2020	06:19	1	90	60	1	88	1	20	1	36.1	2	2		2	999	2	999	999	0	0	3	N√O S	136	2	3	1	999	02/01/2020	2	4	
327	21	1	2	3	32	1	1/13/2020	10:29	1	127	44	1	106	2	999	1	35	1	2	999	1	100	2	999	3	3	2	1	999	441	1	1	1	999	999	2	2	
328	21	1	2	3	36	2	1/15/2020	07:46	1	116	63	1	97	2	999	1	36.5	1	2	999	1	100	2	999	2	2	2	1	999	999	1	2	1	999	15/01/2020	1	3	
329	21	1	1	3	37	1	1/17/2020	01:22	1	138	72	1	94	2	999	2	999	1	2	999	2	999	2	999	4	3	3	2	999	108	2	2	1	05:30	17/01/2020	2	2	
330	25	1	2	2	13	5	1/6/2020	12:06	1	90	60	1	105	2	999	2	999	1	2	999	1	99	2	999	3	3	2	1	999	109	2	999	4	14:25	06/01/2020	3	2	
331	25	1	2	2	14	5	1/16/2020	22:48	1	85	50	1	61	2	999	2	999	999	2		2	999	2	999	3	3	8	1	999	999	1	2	1	999	999	1	3	
332	25	1	2	2	16	5	1/27/2020	15:58	1	92	55	1	130	2	999	1	37.6	1	2	999	1	100	2	999	4	4	2	1	999	62	1	1	2	999	999	2	2	4
333	43	1	4	3	37	1	1/14/2020	15:46	1	130	84	1	117	2	999	2	999	999	2		1	99	2	999	1	2	12	2	999	999	2	2	1	999	999	1	3	
334	43	1	4	3	38	1	1/20/2020	00:43	1	128	79	2	999	2	999	2	999	2	2		2	999	2	999	3	3	7	1	999	999	2	1	2	999	999	1	3	2
335	27	1	3	3	40	3	1/29/2020	20:33	1	130	83	1	83	2	999	1	36.8	2	2		1	99	2	999	2	2	12	1	999	57	2	2	1	999	999	2	1	
336	27	1	3	3	41	3	1/30/2020	09:43	1	132	79	1	92	2	999	1	36.7	2	2		1	99	2	999	2	2	12	1	999	999	1	1	2	999	999	1	3	2
337	39	1	3	3	40	1	1/22/2020	19:58	1	107	62	1	77	2	999	2	999	1	2	999	1	999	2	999	2	2	2	1	999	117	2	2	1	999	999	2	2	
338	30	3	2	3	40	1	1/7/2020	02:24	1	153	94	2	999	2	999	2	999	1	2	999	1	96	2	999	4	4	2	1	999	999	1	2	2	999	07/01/2020	1	3	1
339	22	1	2	3	31	1	1/2/2020	20:40	1	126	82	1	92	2	999	1	36.5	1	2	999	1	100	2	999	3	2	2	2	999	999	2	999	1	999	999	1	3	
340	25	3	1	1	10	1	1/17/2020	20:59	1	103	65	1	73	2	999	1	36.8	1	2	999	1	99	2	999	3	3	8	1	999	999	1	2	1	999	999	1	3	
341	25	1	4	1	12	1	1/7/2020	08:38	1	114	66	1	85	2	999	1	37	1	1	5	1	99	2	999	3	3	2	1	999	999	2	2	999	999	999	1	3	
342	40	1	1	3	39	1	1/17/2020	16:51	1	73	55	1	81	2	999	1	35.9	2	2		1	99	2	999	2	2	2	1	999	126	2	1	1	03:30	18/01/2020	2	2	
343	34	2	2	2	26	1	1/15/2020	12:31	1	107	68	2	999	2	999	1	36.3	1	2	999	2	999	2	999	3	2	5	2	999	414	2	999	4	19:25	15/01/2020	3	2	
344	31	1	1	999	999	1	1/20/2020	17:44	1	166	99	1	98	2	999	1	36.9	2	2		1	99	2	999	3	3	8	1	999	999	2	2	1	999	999	1	3	
345	20	1	1	2	15	1	1/29/2020	15:20	1	106	65	1	103	2	999	1	37	2	2		1	99	2	999	2	2	7	1	999	999	1	1	1	999	999	1	3	
346	34	1	3	999	999	1	1/12/2020	09:23	1	101	45	1	79	2	999	2	999	1	2	999	1	99	2	999	4	2	8	2	999	182	2	2	1	999	999	2	2	
347	34	1	5	2	22	12	1/8/2020	12:52	1	100	70	1	88	2	999	1	36.2	999	2		2	999	2	999	999	0	0	3	N√O S	999	2	3	1	999	08/01/2020	1	4	
348	31	1	5	2	18	1	1/30/2020	17:47	1	113	66	1	90	2	999	1	35.2	1	2	999	1	99	2	999	3	3	7	1	999	999	2	2	1	999	999	1	3	
349	22	1	1	3	36	1	1/28/2020	15:01	1	100	55	1	84	2	999	1	36.4	1	2	999	1	98	2	999	2	2	8	1	999	300	1	1	1	999	999	2	2	
350	33	3	3	2	15	1	1/4/2020	21:19	1	110	70	1	73	1	22	1	36.7	2	2		1	99	2	999	2	2	8	1	999	180	2	1	999	999	43834	2	2	
351	27	2	1	1	12	6	1/7/2020	16:21	1	99	71	1	83	2	999	1	36.7	1	1	4	1	99	2	999	3	3	2	1	999	999	2	2	999	999	999	1	3	
352	27	2	1	2	13	12	1/13/2020	15:07	1	93	65	1	95	2	999	1	36.3	999	2		1	100	2	999	1	2	5	2	999	999	1	2	1	00:30	14/01/2020	1	3	
353	24	1	1	1	7	1	1/28/2020	00:49	1	100	60	1	70	2	999	1	35.6	1	2	999	2	999	2	999	2	2	6	1	999	166	1	1	1	05:37	28/01/2020	2	2	
354	33	2	4	3	38	1	1/13/2020	00:38	1	119	69	1	86	2	999	1	35.6	999	2		1	99	2	999	3	3	7	1	999	999	1	1	2	999	999	1	3	2
355	22	1	2	3	29	1	1/23/2020	19:11	1	116	56	1	78	2	999	1	37	1	2	999	1	99	2	999	2	2	9	1	999	999	1	1	1	999	999	1	3	
356	22	1	2	3	29	7	1/17/2020	12:24	1	108	56	1	85	2	999	1	36.4	1	2	999	1	99	2	999	2	3	2	2	999	999	2	2	1	999	999	1	3	
357	35	1	2	3	37	1	1/11/2020	16:47	1	135	87	1	101	2	999	1	36.9	999	2		1	99	2	999	2	2	10	1	999	999	2	2	999	999	999	1	3	
358	38	1	2	3	28	1	1/17/2020	19:26	1	112	71	1	98	2	999	1	36.1	2	2		1	99	2	999	2	2	8	1	999	999	1	1	1	999	999	1	3	
359	30	1	2	3	38	1	1/27/2020	13:42	1	106	65	1	81	2	999	1	35.9	999	2		1	99	2	999	3	3	2	1	999	78	1	2	1	999	999	2	2	
360	28	1	4	1	10	3	1/2/2020	08:47	1	120	80	1	75	2	999	1	36.2	1	2	999	2	999	2	999	2	3	2	2	999	999	1	999	2	999	02/01/2020	1	3	3
361	36	1	3	3	38	1	1/15/2020	01:02	1	112	64	1	64	2	999	1	35.9	999	2		1	99	2	999	2	3	7	2	999	999	1	1	2	999	999	1	3	2
362	27	1	2	3	27	1	1/3/2020	09:41	1	119	64	1	74	2	999	1	35.6	1	1	5	1	99	2	999	3	3	2	1	999	101	2	1	1	999	03/01/2020	2	2	
363	27	1	2	3	36	1	1/3/2020	22:37	1	122	62	1	81	2	999	2	999	2	2		1	99	2	999	2	3	8	2	999	83	2	2	1	999	04/01/2020	2	2	
364	24	1	1	2	25	1	1/6/2020	17:06	1	116	57	1	100	2	999	1	36.3	1	2	999	1	99	2	999	1	2	12	2	999	999	2	2	1	999	06/01/2020	1	3	
365	30	1	3	1	8	1	1/2/2020	07:24	2	999	999	2	999	2	999	2	999	1	2	999	2	999	2	999	999	0	0	3	N√O S	999	2	3	1	999	02/01/2020	1	4	
366	30	1	3	1	10	1	1/19/2020	21:26	1	105	61	1	99	2	999	1	36.8	1	2	999	1	94	2	999	2	2	9	1	999	69	2	1	1	999	999	2	1	
367	30	1	3	1	12	1	1/30/2020	10:21	1	99	67	1	89	2	999	1	36.3	1	2	999	1	99	2	999	1	2	2	2	999	24	1	1	1	13:50	30/01/2020	3	1	
368	31	2	2	3	39	1	1/15/2020	22:26	1	119	70	1	102	2	999	1	36.7	1	2	999	1	99	2	999	1	2	2	2	999	999	1	2	1	999	999	1	3	
369	30	1	2	3	37	1	1/6/2020	20:22	1	129	69	2	999	2	999	2	999	1	2	999	2	999	2	999	2	4	2	2	999	999	1	2	1	999	999	1	3	
370	38	1	1	3	38	4	1/2/2020	18:51	1	127	86	1	74	2	999	2	999	1	2	999	1	99	2	999	2	2	8	2	999	99	2	999	1	999	999	2	1	
371	38	1	1	3	40	1	1/14/2020	04:33	1	123	69	1	69	1	20	1	36	999	2		2	999	2	999	999	0	0	3	N√O S	999	2	3	1	999	999	1	4	
372	38	1	1	3	40	4	1/14/2020	17:35	1	109	71	1	71	2	999	2	999	999	2		1	100	2	999	3	3	2	1	999	115	2	1	1	23:30	14/01/2020	2	2	
373	38	1	1	3	40	4	1/15/2020	22:28	1	100	70	2	999	2	999	2	999	999	2		2	999	2	999	999	0	0	3	N√O S	72	2	3	1	999	16/01/2020	2	4	
374	33	1	6	2	14	1	1/6/2020	15:45	1	119	80	1	116	2	999	1	37	1	2	999	1	98	2	999	3	3	2	1	999	999	2	2	1	999	999	1	3	
375	31	1	1	3	40	1	1/2/2020	19:43	1	124	76	1	81	2	999	2	999	2	2		1	99	2	999	2	2	2	1	999	77	2	999	1	999	999	2	1	
376	31	1	1	3	40	1	1/3/2020	15:31	1	120	72	1	93	2	999	2	999	1	2	999	1	99	2	999	1	1	2	1	999	139	2	1	1	999	03/01/2020	2	1	
377	31	1	1	3	41	1	1/6/2020	10:38	1	132	75	1	95	2	999	1	36.1	2	2		1	99	2	999	2	2	12	1	999	999	1	1	1	999	06/01/2020	1	3	
378	31	1	1	3	41	1	1/7/2020	07:07	1	116	77	1	104	2	999	2	999	2	2		1	99	2	999	2	2	12	1	999	999	2	1	2	999	999	1	3	1
379	20	1	1	3	37	1	1/16/2020	07:30	1	137	79	1	84	2	999	2	999	1	2	999	1	100	2	999	2	2	7	1	999	999	1	1	2	9999	999	1	3	2
380	31	1	2	3	40	12	1/5/2020	19:31	1	117	70	1	70	2	999	1	36.2	2	2		1	99	2	999	1	2	12	2	999	194	1	2	1	999	05/01/2020	2	2	
381	31	1	2	3	41	12	1/8/2020	07:48	1	114	72	2	999	2	999	1	37.2	999	2		1	99	1	77	2	2	12	1	999	82	2	1	3	12:00	08/01/2020	2	1	
382	22	1	1	3	41	1	1/12/2020	18:04	1	129	86	1	85	2	999	1	37	1	2	999	1	98	2	999	3	3	2	1	999	999	1	2	2	999	999	1	3	2
383	31	1	4	3	39	1	1/31/2020	19:33	1	107	69	1	90	2	999	1	37	2	2		1	99	2	999	2	2	2	1	999	207	2	1	1	999	999	2	2	
384	31	1	3	3	37	1	1/10/2020	13:07	1	94	60	1	84	2	999	1	36.1	999	2		1	99	2	999	2	2	2	1	999	333	2	999	4	16:40	10/01/2020	3	2	
385	27	1	4	2	17	1	1/2/2020	10:15	2	999	999	2	999	2	999	2	999	1	2	999	2	999	2	999	4	4	2	1	999	45	2	2	1	999	02/01/2020	2	2	
386	28	1	3	3	36	3	1/12/2020	09:05	1	128	72	2	999	2	999	2	999	1	2	999	2	999	2	999	4	3	10	2	999	95	2	2	1	999	999	2	2	
387	28	1	3	3	36	3	1/14/2020	09:53	1	138	96	1	122	2	999	1	36.4	1	2	999	1	99	1	140	3	3	10	1	999	7	2	2	2	999	999	2	1	1
388	24	1	1	2	14	1	1/25/2020	06:16	1	129	61	1	104	2	999	2	999	1	2	999	1	96	2	999	1	3	2	2	999	44	2	2	999	999	999	2	2	
389	19	1	1	3	31	1	1/30/2020	16:11	1	101	66	1	90	2	999	1	37	1	2	999	1	100	2	999	1	2	2	2	999	999	1	2	1	999	999	1	3	
390	27	2	1	3	38	1	1/11/2020	21:01	1	101	72	1	77	2	999	1	36.6	999	2		1	99	2	999	2	2	2	1	999	109	2	1	1	999	999	2	1	
391	27	2	1	3	39	1	1/20/2020	08:06	1	98	55	1	90	2	999	1	36.2	999	2		1	99	2	999	2	3	7	2	999	999	1	2	1	999	999	1	3	
392	30	1	2	3	39	1	1/12/2020	09:54	1	118	62	2	999	2	999	2	999	1	2	999	2	999	2	999	3	3	6	1	999	106	2	1	1	999	999	2	2	

393	30	1	2	3	38	1	1/13/2020	05:32	1	108	59	1	86	2	999	1	36.3	999	2		1	99	2	999	2	2	8	1	999	158	1	2	1	999	999	2	2	
394	32	999	4	2	26	1	1/13/2020	09:15	1	98	64	1	100	2	999	1	36.3	1	2	999	1	99	2	999	2	3	8	2	999	999	2	2	3	17:20	13/01/2020	1	3	
395	20	1	2	1	6	1	1/4/2020	14:04	1	138	77	1	102	2	999	1	36.3	2	2		1	100	2	999	2	2	8	1	999	999	1	2	1	999	04/01/2020	1	3	
396	24	2	1	1	7	1	1/18/2020	14:01	1	119	77	1	76	2	999	1	36.1	1	2	999	1	99	2	999	2	2	8	1	999	999	2	1	1	999	999	1	3	
397	21	1	999	3	29	2	1/20/2020	06:46	1	139	92	1	108	1	20	1	37	999	2		2	999	1	108	4	4	2	2	999	54	2	2	2	999	999	2	2	4
398	24	999	1	3	38	1	1/28/2020	12:01	1	104	68	1	102	2	999	1	36.3	999	2		1	99	2	999	3	3	2	1	999	126	1	2	1	999	999	2	2	
399	24	1	1	3	38	1	1/30/2020	02:49	1	123	79	1	97	2	999	1	36.1	999	2		2	999	2	999	999	0	0	3	N√O S	109	2	3	4	04:30	30/01/2020	3	4	
400	28	1	3	3	37	3	1/6/2020	08:10	1	118	59	1	78	2	999	1	36.3	1	2	999	1	99	2	999	3	3	2	1	999	999	1	1	2	999	06/01/2020	1	3	1
401	33	2	5	1	10	1	1/18/2020	11:37	1	111	73	1	88	2	999	1	36.8	2	2		2	999	2	999	2	2	8	1	999	999	2	2	1	999	999	1	3	
402	25	2	2	1	7	1	1/4/2020	15:03	1	107	62	1	88	2	999	1	36.3	1	2	999	1	99	2	999	3	3	2	1	999	999	2	2	1	999	04/01/2020	1	3	
403	27	1	1	3	37	1	1/24/2020	02:25	1	138	88	1	89	2	999	1	36.7	999	2		2	999	2	999	999	0	0	3	N√O S	999	1	3	2	999	999	1	4	1
404	31	999	1	3	38	1	1/23/2020	10:22	1	114	74	1	80	2	999	1	36.3	2	2		1	100	2	999	2	2	12	1	999	999	2	1	2	999	999	1	3	1
405	36	1	1	3	36	1	1/26/2020	18:48	1	110	66	1	91	2	999	1	36.1	1	2	999	1	100	2	999	1	2	3	2	999	87	2	1	1	999	26/01/2020	2	1	
406	30	1	1	3	30	1	1/7/2020	20:44	1	120	60	1	80	2	999	1	37	1	2	999	2	999	2	999	3	3	2	1	999	999	2	2	1	999	999	1	3	
407	30	1	1	3	32	1	1/21/2020	19:21	1	128	49	1	104	2	999	1	36.3	999	2		1	99	2	999	2	2	6	1	999	999	2	1	1	999	999	1	3	
408	21	1	1	2	15	1	1/2/2020	21:56	1	103	56	1	82	2	999	1	35.6	1	2	999	1	100	2	999	2	2	2	1	999	999	1	999	1	999	999	1	3	
409	22	1	1	3	39	1	1/26/2020	06:47	1	115	69	1	84	2	999	2	999	1	2	999	2	999	2	999	3	3	2	1	999	93	2	999	4	08:20	26/01/2020	3	2	
410	30	1	1	3	39	1	1/30/2020	01:55	1	102	72	1	75	2	999	1	35.6	2	2		1	98	2	999	3	3	2	1	999	80	1	2	1	999	999	2	2	
411	29	1	1	2	15	1	1/14/2020	08:50	1	144	100	1	107	2	999	1	35.9	1	2	999	1	99	2	999	4	4	3	1	999	50	2	1	1	999	14/01/2020	2	2	
412	37	1	3	3	32	12	1/27/2020	09: 11	1	132	78	1	126	2	999	1	35.9	1	2	999	1	100	2	999	3	3	2	1	999	79	2	1	1	17:25	27/01/2020	2	2	
413	34	2	3	3	35	1	1/28/2020	14:57	1	111	69	1	97	2	999	1	36.4	1	2	999	1	99	2	999	2	2	2	1	999	999	1	1	1	999	999	1	3	
414	34	2	3	3	35	1	1/29/2020	17:08	1	110	63	1	88	2	999	1	36.6	2	2		1	99	2	999	1	2	12	2	999	112	2	2	1	999	999	3	1	
415	19	1	1	2	25	1	1/9/2020	22:07	1	102	56	2	999	2	999	2	999	999	2		2	999	2	999	2	2	12	1	999	128	2	999	4	01:15	10/01/2020	3	2	
416	19	1	1	2	25	1	1/10/2020	20:43	1	100	60	1	74	2	999	2	999	1	2	999	2	999	2	999	2	2	8	1	999	999	1	1	1	999	999	1	3	
417	19	1	1	2	26	1	1/14/2020	13:21	1	105	62	1	101	2	999	1	35.9	1	2	999	1	99	2	999	2	2	2	1	999	999	2	1	1	999	999	1	3	
418	29	1	3	3	39	1	1/24/2020	15:06	1	102	59	1	85	2	999	2	999	2	2		1	99	2	999	1	2	12	2	999	193	2	2	1	999	999	2	2	
419	37	1	3	2	25	1	1/29/2020	09:49	1	103	49	1	74	2	999	1	35.9	1	2	999	1	99	2	999	2	2	2	1	999	999	1	2	1	999	999	1	3	
420	31	1	2	2	21	3	1/16/2020	16:51	1	142	98	1	90	2	999	1	36.6	2	2		1	99	2	999	3	3	12	1	999	999	2	1	1	999	999	1	3	
421	34	1	2	2	18	1	1/31/2020	19:55	1	103	58	1	71	2	999	2	999	1	2	999	2	999	2	999	1	2	2	2	999	185	2	2	1	01:00	01/02/2020	2	2	
422	36	2	3	3	40	1	1/25/2020	08:43	1	119	76	1	94	2	999	1	35.7	1	2	999	1	99	2	999	1	2	2	2	999	999	1	2	2	999	999	1	3	1
423	32	1	3	3	35	999	1/27/2020	13:39	1	119	62	1	62	2	999	1	36.3	1	2	999	1	100	2	999	2	2	5	1	999	149	2	999	4	16:10	27/01/2020	3	2	
424	35	1	1	3	32	1	1/3/2020	09:53	1	119	60	1	118	2	999	1	35.1	1	1	7	1	98	2	999	4	4	2	1	999	107	2	2	1	20:05	03/01/2020	2	2	
425	27	1	2	3	39	1	1/25/2020	15:21	1	121	76	1	81	2	999	2	999	999	2		2	999	2	999	2	4	2	2	999	84	2	1	2	999	999	2	2	1
426	23	1	2	1	7	1	1/7/2020	14:58	1	114	65	1	87	2	999	1	35	1	1	3	1	99	2	999	3	3	8	1	999	52	1	2	1	999	07/01/2020	2	2	
427	24	2	2	3	39	1	1/27/2020	03:55	1	100	60	1	84	2	999	1	36	999	2		2	999	2	999	999	0	0	3	N√O S	999	1	3	2	999	999	1	4	2
428	20	1	2	2	15	1	1/2/2020	20:12	1	132	75	1	73	2	999	1	36.8	2	2		1	99	2	999	2	2	12	1	999	999	2	999	1	999	999	1	3	
429	20	1	2	2	16	3	1/4/2020	16:11	1	134	78	1	74	2	999	1	36.9	1	2	999	1	99	2	999	3	3	2	1	999	49	1	1	1	999	04/01/2020	2	2	
430	21	1	1	3	35	1	1/3/2020	999	2	999	999	2	999	2	999	2	999	999	2		2	999	2	999	2	4	12	2	999	999	2	999	1	999	03/01/2020	1	3	
431	21	1	1	3	37	1	1/8/2020	00:42	1	118	74	1	89	2	999	1	35.9	1	2	999	2	999	2	999	2	2	2	1	999	999	2	1	1	999	999	1	3	
432	21	1	1	3	36	1	1/12/2020	07:13	1	129	77	1	77	2	999	2	999	1	2	999	1	99	2	999	4	4	2	1	999	57	2	2	1	16:20	12/01/2020	2	2	
433	25	1	2	1	8	1	1/3/2020	16:53	1	116	71	1	60	2	999	1	35.6	2	2		1	100	2	999	2	3	8	2	999	999	1	2	1	999	03/01/2020	1	3	
434	35	1	2	1	6	1	1/4/2020	08:35	1	106	62	1	61	2	999	1	35.9	1	2	999	1	99	2	999	2	2	2	1	999	999	1	1	2	999	04/01/2020	1	3	3
435	21	2	1	1	10	1	1/17/2020	09:54	1	109	63	1	84	2	999	1	37.1	1	2	999	1	100	2	999	3	3	2	1	999	999	1	2	2	999	999	1	3	3
436	27	1	999	999	999	999	1/16/2020	14:09	1	102	49	1	77	2	999	1	35.9	1	2	999	1	99	2	999	4	2	2	2	999	999	2	2	1	999	999	1	3	
437	28	1	1	3	40	1	1/10/2020	18:46	1	110	70	1	83	2	999	2	999	1	2	999	2	999	2	999	2	2	2	1	999	999	1	1	2	999	999	1	3	1
438	25	1	2	3	39	1	1/25/2020	21:55	1	114	77	1	110	2	999	1	36.5	1	2	999	2	999	2	999	4	4	2	1	999	999	1	2	2	999	999	1	3	2
439	25	2	2	3	40	1	1/13/2020	10:33	1	121	75	1	103	2	999	1	36.4	1	1	7	1	99	2	999	4	4	2	1	999	87	1	2	1	999	999	2	2	
440	25	2	2	3	39	11	1/17/2020	04:39	1	127	86	1	96	2	999	1	35.6	999	2		2	999	2	999	999	0	0	3	N√O S	119	1	2	3	16/01/:	10:30	2	4	
441	21	1	2	3	29	1	1/27/2020	12:31	1	124	85	1	85	2	999	1	36.7	2	2		2	999	2	999	999	0	0	3	N√O S	89	1	3	1	18:30	27/01/2020	2	4	
442	20	1	2	3	29	1	1/28/2020	22:49	1	143	77	1	82	2	999	1	36.3	1	2	999	1	100	2	999	3	3	12	1	999	36	2	2	1	999	999	2	2	
443	25	2	3	3	37	1	1/4/2020	18:11	1	102	55	1	76	2	999	1	35.2	1	2	999	1	99	2	999	3	3	2	1	999	116	1	2	1	999	04/01/2020	2	2	
444	25	999	3	3	40	1	1/19/2020	11:34	1	115	79	1	68	2	999	1	36.3	1	2	999	2	999	2	999	2	2	2	2	999	123	2	999	4	13:51	19/01/2020	3	2	
445	25	2	1	3	37	4	1/4/2020	05:38	1	130	90	1	118	2	999	1	36.3	999	2		2	999	2	999	999	0	0	3	N√O S	262	2	3	5	999	04/01/2020	2	4	
446	18	1	2	1	8	1	1/17/2020	23:00	1	108	48	1	113	2	999	1	37.4	1	2	999	1	98	2	999	4	4	2	1	999	50	1	1	1	01:40	18/01/2020	2	2	
447	40	2	5	2	26	1	1/24/2020	20:05	1	110	56	1	78	2	999	1	36.3	1	2	999	1	99	2	999	2	2	2	1	999	999	1	2	1	999	999	1	3	
448	25	1	3	2	23	1	1/3/2020	21:02	1	103	51	2	999	2	999	2	999	1	2	999	2	999	2	999	2	4	2	2	999	999	1	2	1	999	999	1	3	
449	25	1	3	2	26	1	1/27/2020	18:28	1	112	66	1	86	2	999	1	36.4	1	2	999	1	100	2	999	1	2	2	2	999	999	2	1	1	00:50	28/01/2020	1	3	
450	26	1	3	3	40	6	1/15/2020	09:15	1	122	68	1	97	2	999	1	35.2	999	2		1	99	2	999	2	2	7	1	999	125	2	2	1	999	15/01/2020	2	2	
451	26	999	3	3	41	1	1/23/2020	09:31	1	118	78	1	90	2	999	1	37	2	2		1	100	2	999	2	2	12	1	999	999	2	1	2	999	999	1	3	2
452	36	2	3	3	40	1	1/14/2020	00:12	1	108	56	1	73	2	999	2	999	1	2	999	2	999	2	999	3	3	2	1	999	999	1	2	1	999	999	1	3	
453	32	1	2	2	20	10	1/22/2020	10:18	1	97	58	1	94	2	999	1	36.4	2	2		1	100	2	999	2	2	12	1	999	999	2	1	1	999	999	1	3	
454	28	1	1	3	41	1	1/30/2020	21:47	1	119	64	1	102	2	999	1	38	1	2	999	1	100	2	999	3	3	5	1	999	43	2	1	2	999	999	2	2	1
455	28	1	3	3	39	1	1/17/2020	13:30	1	128	90	1	91	2	999	1	36.1	1	2	999	1	100	2	999	3	3	2	1	999	999	1	2	5	17:00	18/01/2020	1	3	
456	21	1	3	3	38	1	1/19/2020	22:17	1	119	64	1	100	2	999	1	36.7	999	2		1	99	2	999	2	3	10	2	999	63	2	2	1	999	999	2	2	
457	21	1	3	3	38	1	1/21/2020	20:51	1	131	59	1	89	2	999	1	36.7	999	2		1	99	2	999	2	3	8	2	999	79	1	2	1	999	21/01/2020	2	2	
458	21	1	3	3	38	1	1/24/2020	12:47	1	120	70	1	67	2	999	2	999	2	2		1	99	2	999	3	3	7	1	999	133	1	2	1	999	999	2	2	
459	27	1	3	1	8	1	1/17/2020	12:22	1	113	68	1	85	2	999	1	36.1	1	2	999	1	99	2	999	1	2	2	2	999	145	2	2	1	999	17/01/2020	2	2	
460	24	2	3	3	40	1	1/17/2020	14:21	1	115	75	1	95	2	999	1	36.6	1	2	999	1	99	2	999	1	3	2	2	999	59	2	2	1	999	999	2	2	

461	24	2	3	3	40	1	1/20/2020	15:26	1	114	69	1	97	2	999	1	36.7	1	2	999	1	99	2	999	1	2	2	2	999	999	1	2	1	999	999	1	3	
462	24	2	3	3	40	1	1/21/2020	15:12	1	109	75	1	79	2	999	1	36.4	2	2		1	98	2	999	3	3	2	1	999	48	2	1	1	999	21/01/2020	2	2	
463	24	2	3	3	41	1	1/24/2020	08:19	1	113	75	1	87	2	999	1	36.2	1	2	999	1	99	2	999	3	2	12	1	999	241	1	1	2	999	999	2	2	3
464	25	1	3	3	37	1	1/11/2020	23:33	1	116	57	1	78	2	999	1	37	1	2	999	1	99	2	999	1	2	2	2	999	237	2	999	4	03:30	12/01/2020	3	2	
465	20	1	1	3	37	1	1/16/2020	15:45	1	122	82	1	92	2	999	1	35.6	2	2		1	99	2	999	1	2	12	2	999	105	2	2	1	20:30	16/01/2020	2	1	
466	20	1	1	3	39	1	1/30/2020	07:25	1	130	90	2	999	2	999	2	999	2	2		2	999	2	999	4	4	2	1	999	75	2	1	2	999	999	2	2	1
467	27	2	2	3	38	1	1/9/2020	23:08	1	134	63	2	999	2	999	2	999	1	2	999	2	999	2	999	3	3	2	1	999	999	1	1	1	999	999	1	3	
468	27	2	2	3	39	3	1/15/2020	16:58	2	999	999	2	999	2	999	2	999	1	2	999	2	999	2	999	2	2	7	1	999	999	1	1	2	999	15/01/2020	1	3	2
469	23	999	999	3	39	1	1/19/2020	12:04	1	129	82	1	102	2	999	1	35.2	999	2		2	999	2	999	999	0	0	3	N√O S	999	2	3	1	999	19/01/2020	1	4	
470	23	1	3	3	41	1	1/24/2020	03:44	1	110	80	1	94	2	999	1	36	999	2		1	99	2	999	999	0	0	3	N√O S	999	1	3	2	05:05	24/01/2020	1	4	2
471	22	2	2	1	9	1	1/12/2020	20:42	1	116	74	1	96	2	999	1	36.1	1	2	999	1	99	2	999	2	2	8	1	999	999	1	1	1	999	999	1	3	
472	19	1	1	1	6	1	1/2/2020	08:51	2	999	999	2	999	2	999	2	999	1	2	999	2	999	2	999	2	2	2	1	999	999	2	999	999	999	999	1	3	
473	22	2	1	3	38	5	1/2/2020	00:27	1	156	89	1	111	1	20	1	36.1	2	2		2	999	2	999	999	0	0	3	N√O S	999	2	3	999	999	02/01/2020	1	4	
474	25	1	2	1	6	1	1/9/2020	15:44	1	124	40	1	84	2	999	1	36.7	1	2	999	1	100	2	999	2	2	2	1	999	999	2	2	1	999	999	1	3	
475	22	1	1	3	37	11	1/4/2020	06:02	1	120	71	1	95	2	999	1	36.1	1	2	999	2	999	2	999	3	3	2	1	999	88	2	1	1	999	04/01/2020	2	2	
476	22	1	1	3	40	1	1/26/2020	08:46	1	114	62	1	84	2	999	1	35.2	1	2	999	1	99	2	999	2	2	2	1	999	74	2	1	1	999	999	2	1	
477	22	1	1	3	41	1	1/28/2020	09:17	1	114	62	1	93	2	999	1	35.4	2	2		1	99	2	999	2	2	12	1	999	999	2	1	2	999	999	1	3	2
478	32	1	1	2	24	1	1/8/2020	22:08	1	110	64	1	74	2	999	1	36.3	1	2	999	1	99	2	999	1	2	2	2	999	217	2	2	1	999	09/01/2020	2	2	
479	20	1	2	2	13	1	1/9/2020	14:15	1	100	64	1	101	1	36	2	999	999	2		1	99	2	999	2	2	8	1	999	999	2	2	999	999	999	1	3	
480	24	3	1	1	11	1	1/9/2020	17:25	1	101	46	1	81	2	999	1	36.7	1	2	999	1	100	2	999	2	2	2	1	999	999	2	1	1	999	999	1	3	
481	20	1	1	3	33	1	1/27/2020	19:35	1	90	60	2	999	2	999	2	999	1	2	999	2	999	2	999	2	2	2	1	999	100	1	2	1	999	999	2	1	
482	26	1	1	3	39	1	1/6/2020	19:47	1	111	69	2	999	2	999	2	999	2	2		2	999	2	999	2	2	12	1	999	103	1	2	1	999	06/01/2020	2	1	
483	26	1	1	3	40	1	1/10/2020	16:42	1	105	67	1	92	2	999	1	35.2	999	2		1	99	2	999	3	3	7	1	999	999	1	1	2	999	999	1	3	2
484	26	1	2	3	33	1	1/10/2020	11:06	1	106	51	1	70	2	999	2	999	999	2		1	99	2	999	2	2	7	1	999	378	1	2	1	999	10/01/2020	2	2	
485	25	1	2	3	35	1	1/17/2020	17:24	1	118	77	1	118	2	999	1	36.3	2	2		1	99	2	999	1	2	9	2	999	999	2	1	999	999	999	1	3	
486	21	1	2	2	27	1	1/5/2020	11:15	1	138	49	1	110	2	999	1	35.9	1	1	9	1	99	2	999	4	4	2	1	999	25	1	1	1	999	05/01/2020	2	2	
487	28	1	1	2	22	1	1/17/2020	17:25	1	103	66	1	79	2	999	1	36.4	2	2		1	100	2	999	2	2	10	1	999	999	1	1	1	999	999	1	3	
488	28	1	1	2	23	4	1/18/2020	08:18	1	103	58	1	81	2	999	1	36.1	1	2	999	1	100	2	999	2	2	2	1	999	999	1	1	2	999	999	1	3	999
489	29	1	5	2	25	6	1/20/2020	13:22	1	110	66	1	87	2	999	1	36.4	999	2		1	99	2	999	3	3	7	1	999	999	2	2	1	999	999	1	3	
490	23	2	3	3	36	1	1/26/2020	12:18	1	120	80	1	106	2	999	2	999	1	2	999	2	999	2	999	999	0	0	3	N√O S	132	2	3	1	999	999	2	4	
491	29	1	1	3	39	3	1/23/2020	08:49	1	131	78	1	88	2	999	1	36.7	1	2	999	1	99	2	999	2	2	2	1	999	999	2	1	1	999	999	1	3	
492	29	1	1	3	41	3	1/30/2020	09:27	1	120	90	1	108	2	999	1	36.3	1	2	999	1	99	2	999	2	2	12	1	999	999	1	1	2	999	999	1	3	1
493	28	1	1	3	38	1	1/10/2020	09:32	1	124	80	1	99	2	999	1	36.7	1	2	999	2	999	2	999	1	2	2	2	999	148	2	2	1	999	999	2	2	
494	28	1	1	3	39	1	1/21/2020	6:00:0	1	125	80	1	103	2	999	1	36.6	999	2		1	99	2	999	3	3	2	1	999	60	2	1	1	999	21/01/2020	2	2	
495	28	1	1	3	40	1	1/27/2020	17:43	1	113	78	1	96	2	999	1	36.4	999	2		1	99	2	999	3	3	2	1	999	67	1	2	1	999	999	2	2	
496	28	1	1	3	40	1	1/28/2020	17:24	1	122	88	1	102	2	999	1	36.1	999	2		1	100	2	999	4	4	2	1	999	81	2	2	1	999	999	2	2	
497	20	999	2	3	39	1	1/16/2020	07:46	1	125	68	1	103	2	999	1	35	999	2		2	999	2	999	4	4	2	1	999	999	2	1	2	999	999	1	3	2
498	20	1	3	3	36	1	1/17/2020	12:10	1	104	60	1	93	2	999	1	35.7	1	2	999	1	99	2	999	3	3	2	1	999	999	1	1	2	999	999	1	3	2
499	24	1	3	999	999	1	1/23/2020	20:14	1	130	60	2	999	2	999	2	999	1	2	999	2	999	2	999	3	3	8	1	999	106	1	2	2	07:50	23/01/2020	2	2	3
500	28	1	4	2	25	7	1/7/2020	15:54	1	121	60	1	101	2	999	1	35.4	1	1	7	1	100	2	999	4	4	2	1	999	999	2	999	999	999	999	1	3	
501	28	1	4	2	24	1	1/22/2020	13:59	1	107	52	1	78	2	999	1	35	1	2	999	1	100	2	999	1	2	2	2	999	71	2	2	1	999	999	2	1	
502	22	1	1	2	19	1	1/15/2020	12:25	1	103	74	1	88	2	999	1	35.6	999	2		1	100	2	999	2	2	6	1	999	420	2	999	4	19:25	15/01/2020	3	2	
503	36	1	2	3	40	3	1/12/2020	08:54	1	153	86	2	999	2	999	2	999	1	2	999	2	999	2	999	3	3	12	1	999	36	2	2	2	999	999	2	2	1
504	35	1	1	3	41	1	1/17/2020	16:25	1	114	70	1	84	2	999	1	35.4	2	2		1	98	2	999	2	2	2	1	999	999	1	1	2	999	999	1	3	2
505	24	1	3	3	33	1	1/5/2020	23:33	1	131	73	1	122	2	999	1	36.4	2	2		1	99	2	999	4	3	2	2	999	127	2	2	1	999	06/01/2020	2	2	
506	32	2	1	3	38	1	1/8/2020	14:02	1	139	88	2	999	2	999	1	36.1	1	2	999	2	999	2	999	2	2	3	1	999	78	1	2	1	21:30	08/01/2020	2	1	
507	32	2	1	3	39	1	1/13/2020	06:27	1	130	83	1	80	2	999	1	35.9	999	2		1	99	2	999	2	2	2	1	999	999	2	1	2	999	999	1	3	2
508	32	1	1	3	33	1	1/10/2020	10:33	1	104	63	1	83	2	999	1	36.1	999	2		1	99	2	999	1	2	12	2	999	144	2	2	1	999	999	2	2	
509	32	1	1	3	33	1	1/9/2020	19:16	1	128	59	2	999	2	999	2	999	999	2		2	999	2	999	999	0	0	3	N√O S	44	2	3	1	999	09/01/2020	2	4	
510	24	1	2	3	33	1	1/25/2020	22:48	1	120	80	2	999	2	999	2	999	1	2	999	2	999	2	999	2	2	8	1	999	999	1	1	1	999	999	1	3	
511	21	3	1	3	40	12	1/14/2020	10:45	1	133	75	1	95	2	999	2	999	2	2		1	99	2	999	1	2	12	2	999	135	1	2	1	999	999	2	2	
512	21	2	1	3	40	12	1/20/2020	05:08	1	147	91	1	83	2	999	2	999	1	2	999	1	100	2	999	3	3	2	1	999	142	2	2	1	999	999	2	2	
513	21	2	1	3	40	12	1/20/2020	17:56	1	154	86	1	90	2	999	1	37.1	1	2	999	1	99	2	999	3	3	12	1	999	999	1	2	2	999	999	1	3	2
514	21	1	2	3	39	1	1/14/2020	20:25	1	120	74	1	95	2	999	1	36.3	2	2		1	99	2	999	2	2	7	1	999	115	1	1	1	999	999	2	1	
515	21	1	2	3	40	1	1/21/2020	05:33	1	114	80	1	99	2	999	1	35.9	2	2		1	99	2	999	3	3	2	1	999	107	1	1	2	13:10	21/01/2020	2	2	2
516	20	2	1	3	40	1	1/20/2020	10:12	1	109	67	1	98	2	999	1	36.6	2	2		1	99	2	999	1	2	12	2	999	268	2	2	1	999	999	2	2	
517	20	2	1	3	41	1	1/26/2020	08:50	1	114	74	1	104	2	999	1	37	2	2		1	99	2	999	2	2	12	1	999	160	2	1	1	999	999	2	2	
518	20	2	1	3	41	1	1/26/2020	14:59	1	115	63	1	100	2	999	1	36.8	2	2		1	99	2	999	2	2	8	1	999	999	2	2	1	999	999	1	3	
519	20	2	999	3	41	999	1/28/2020	10:15	1	110	70	1	76	2	999	1	36.2	999	2		2	999	2	999	1	3	12	2	999	999	2	2	2	999	999	1	3	2
520	29	1	1	3	36	1	1/26/2020	10:07	1	115	77	1	105	2	999	1	35.4	2	2		1	99	2	999	2	2	9	1	999	188	1	1	1	999	999	2	2	
521	25	1	1	3	40	1	1/7/2020	00:31	1	120	65	2	999	2	999	2	999	1	2	999	2	999	2	999	4	4	2	1	999	89	1	2	1	06:00	07/01/2020	2	2	
522	25	1	1	3	40	1	1/8/2020	00:22	1	120	64	1	84	2	999	2	999	2	2		2	999	2	999	3	3	10	1	999	999	2	1	2	999	08/01/2020	1	3	1
523	20	1	1	3	28	1	1/13/2020	15:43	1	111	67	1	102	2	999	1	35	999	2		1	99	2	999	3	3	10	1	999	97	2	999	4	18:55	13/01/2020	3	2	
524	35	1	4	3	37	1	1/17/2020	09:57	1	106	61	1	95	2	999	1	35.6	2	2		1	99	2	999	2	2	12	1	999	63	1	2	1	999	999	2	1	
525	20	1	2	3	39	1	1/10/2020	06:16	1	128	75	2	999	2	999	2	999	999	2		2	999	2	999	3	3	2	1	999	999	1	1	1	999	999	1	3	
526	20	1	2	3	39	1	1/10/2020	09:58	1	133	82	2	999	2	999	2	999	999	2		2	999	2	999	4	4	12	1	999	999	1	1	2	999	999	1	3	2
527	34	1	3	1	4	1	1/21/2020	13:51	1	102	66	1	87	2	999	1	36.1	2	2		1	100	2	999	1	1	12	1	999	999	1	2	999	999	999	1	3	
528	30	1	4	3	32	1	1/8/2020	10:52	1	108	58	1	78	2	999	1	35.5	1	2	999	1	99	2	999	3	3	2	1	999	999	999	1	1	999	08/01/2020	1	3	

529	34	1	4	3	34	1	1/3/2020	12:00	1	125	82	1	120	2	999	1	36.5	1	1	3	1	99	2	999	2	3	8	2	999	40	1	2	1	20:40	03/01/2020	2	2	
530	37	1	2	3	36	1	1/30/2020	16:53	1	106	60	1	91	2	999	1	36.1	1	2	999	1	100	2	999	1	2	12	2	999	77	1	2	1	999	999	2	1	
531	36	1	2	3	39	1	1/29/2020	17:36	1	105	60	1	83	2	999	1	35.6	2	2		1	99	2	999	1	2	12	2	999	124	1	2	1	999	999	2	2	
532	22	1	1	2	17	1	1/2/2020	12:52	1	94	60	1	75	2	999	2	999	1	2	999	1	99	2	999	2	2	8	1	999	999	1	999	1	999	999	1	3	
533	31	1	3	2	17	1	1/4/2020	14:39	1	129	78	1	109	2	999	1	35.9	1	2	999	1	99	2	999	2	2	2	1	999	999	2	1	1	999	04/01/2020	1	3	
534	28	1	2	3	38	1	1/7/2020	16:00	1	124	78	1	77	2	999	1	36.2	1	2	999	1	99	2	999	3	3	2	1	999	999	2	1	2	999	999	1	3	2
535	31	1	1	3	34	2	1/7/2020	07:17	1	101	57	1	97	2	999	1	36.6	999	2		1	100	1	87	1	1	12	1	999	13	1	1	1	18:00	07/01/2020	2	1	
536	31	1	1	3	35	2	1/14/2020	13:34	1	111	65	1	99	2	999	1	35.4	1	1	8	1	100	1	123	4	4	2	1	999	81	2	2	1	999	999	2	2	
537	26	1	2	1	11	12	1/12/2020	23:45	1	116	61	1	96	2	999	1	37	1	2	999	1	100	2	999	2	2	8	1	999	90	1	1	1	999	13/01/2020	2	1	
538	26	1	2	1	11	1	1/14/2020	02:44	1	135	78	1	110	2	999	1	36.5	1	2	999	2	999	2	999	999	0	0	3	N√O S	196	1	3	2	999	999	2	4	3
539	31	1	2	999	999	1	1/4/2020	13:19	1	100	64	1	74	2	999	1	36	1	2	999	1	99	2	999	2	2	2	1	999	91	1	1	1	09:30	05/01/2020	2	1	
540	25	1	1	2	16	2	1/30/2020	13:36	1	102	61	1	91	2	999	1	36.3	1	2	999	1	99	2	999	2	2	9	1	999	999	1	1	1	16:45	30/01/2020	1	3	
541	26	1	2	3	30	11	1/4/2020	09:15	1	114	67	1	79	2	999	1	35.9	1	1	9	1	99	2	999	4	4	2	1	999	115	2	1	1	999	04/01/2020	2	2	
542	26	1	2	3	33	1	1/27/2020	10:46	1	103	55	1	75	2	999	1	33.6	1	2	999	1	100	2	999	3	3	2	1	999	999	1	2	1	999	999	1	3	
543	23	1	3	3	31	9	1/21/2020	16:33	1	127	74	1	135	2	999	1	36.7	1	2	999	1	99	2	999	2	2	6	1	999	72	1	2	1	999	999	2	1	
544	26	1	3	3	37	8	1/29/2020	15:18	1	116	62	1	111	2	999	1	35.9	1	2	999	1	99	2	999	2	2	2	1	999	182	1	1	1	21:00	29/01/2020	2	2	
545	35	1	3	1	9	7	1/11/2020	10:17	1	149	89	1	89	2	999	1	36.7	1	2	999	1	99	2	999	4	4	2	1	999	73	2	1	999	999	999	2	2	
546	35	1	3	1	10	12	1/13/2020	04:17	1	134	88	1	88	2	999	1	36.3	1	2	999	2	999	2	999	999	0	0	3	N√O S	37	2	3	1	14:15	13/01/2020	2	4	
547	35	1	3	1	9	12	1/5/2020	20:54	1	144	82	1	85	2	999	1	35.5	1	2	999	1	99	2	999	2	3	3	2	999	126	2	2	999	999	999	2	2	
548	35	1	3	1	8	1	1/4/2020	14:12	1	129	83	1	90	2	999	1	36.6	1	2	999	1	100	2	999	2	2	2	1	999	999	2	1	1	999	04/01/2020	1	3	
549	19	1	2	2	19	1	1/20/2020	19:53	1	97	49	1	86	2	999	1	36.3	1	2	999	1	99	2	999	2	2	12	1	999	999	1	1	1	999	999	1	3	
550	25	1	3	3	33	1	1/2/2020	15:21	1	89	57	1	93	2	999	2	999	1	1	9	1	99	2	999	4	4	2	1	999	999	1	2	1	999	02/01/2020	1	3	
551	35	3	3	1	6	7	1/11/2020	14:15	1	93	53	1	78	2	999	2	999	1	2	999	1	98	2	999	3	3	2	1	999	115	1	1	1	999	999	2	2	
552	35	3	3	1	7	1	1/14/2020	09:38	1	95	69	1	85	2	999	1	36.6	999	2		1	97	2	999	2	2	8	1	999	404	2	1	1	999	999	2	2	
553	21	1	1	3	35	3	1/10/2020	18:18	1	138	77	1	110	2	999	1	37	1	2	999	1	99	2	999	2	2	2	1	999	142	1	2	1	999	999	2	1	
554	21	1	1	3	35	12	1/13/2020	22:30	1	130	90	1	105	2	999	2	999	1	2		2	999	2	999	3	3	2	1	999	30	1	1	1	01:30	14/01/2020	2	1	
555	21	1	1	3	36	12	1/19/2020	20:52	2	999	999	2	999	2	999	2	999	999	2		2	999	2	999	4	4	2	1	999	999	2	1	2	999	999	1	3	1
556	25	1	1	3	40	1	1/2/2020	09:18	2	999	999	2	999	2	999	2	999	1	1	7	2	999	2	999	4	4	2	1	999	999	2	2	1	999	02/01/2020	1	3	
557	26	2	6	2	18	2	1/15/2020	06:13	1	114	76	1	96	2	999	1	37	2	2		1	99	1	81	1	1	12	1	999	87	2	2	1	999	15/01/2020	2	1	
558	18	1	1	1	10	1	1/21/2020	21:00	1	109	61	1	90	2	999	1	36.7	1	2	999	1	99	2	999	4	2	2	2	999	999	2	2	999	999	999	1	3	
559	19	999	1	1	9	1	1/14/2020	21:48	1	114	68	1	90	2	999	1	36.7	1	2	999	1	99	2	999	2	2	8	1	999	999	1	1	1	999	999	1	3	
560	33	1	3	3	39	1	1/27/2020	15:15	1	135	76	1	98	2	999	1	36.7	1	2	999	1	99	2	999	3	3	3	1	999	105	2	2	1	20:30	27/01/2020	2	2	
561	20	1	1	2	20	1	1/7/2020	21:53	1	100	70	1	81	2	999	2	999	1	2	999	2	999	2	999	2	2	2	1	999	999	2	2	1	999	999	1	3	
562	33	1	8	3	40	12	1/30/2020	14:37	1	112	70	1	77	2	999	1	36.2	2	2		1	99	2	999	2	2	10	1	999	98	1	2	1	999	999	2	1	
563	25	1	2	3	31	1	1/20/2020	13:06	1	117	72	1	98	2	999	1	36.1	1	2	999	1	99	2	999	3	3	12	1	999	34	1	1	1	999	999	2	2	
564	18	999	2	3	40	3	1/5/2020	07:30	1	141	84	1	79	2	999	1	36	1	2	999	1	99	2	999	3	3	2	1	999	120	1	2	2	12:35	05/01/2020	2	2	2
565	24	3	1	1	12	1	1/9/2020	20:54	1	122	85	2	999	2	999	1	37	1	2	999	2	999	2	999	3	2	5	2	999	156	1	2	1	999	999	2	2	
566	35	1	5	1	5	9	1/28/2020	11:04	1	104	65	1	88	2	999	1	35.8	1	2	999	1	100	2	999	1	2	8	2	999	999	2	2	999	999	999	1	3	
567	35	1	5	1	5	1	1/27/2020	20:22	1	103	68	1	80	2	999	1	36.4	1	2	999	2	999	2	999	2	3	2	2	999	999	1	2	1	999	999	1	3	
568	25	2	4	3	39	1	1/12/2020	16:00	1	145	83	1	100	2	999	1	35.9	999	2		1	99	2	999	3	2	12	2	999	60	1	2	1	999	999	2	1	
569	25	2	4	3	39	8	1/15/2020	06:00	1	123	74	1	90	2	999	1	36.1	999	2		1	99	2	999	2	2	2	1	999	999	2	1	2	999	999	1	3	2
570	20	1	1	3	39	1	1/20/2020	23:37	1	119	60	1	84	2	999	1	35.6	999	2		1	99	2	999	2	2	12	1	999	999	1	2	2	02:20	20/01/2020	1	3	1
571	36	1	4	3	29	1	1/7/2020	09:07	1	116	57	1	81	2	999	1	35.4	1	1	6	1	99	2	999	3	3	2	1	999	93	1	1	1	999	07/01/2020	2	2	
572	36	1	4	3	33	1	1/28/2020	07:18	1	110	59	1	75	2	999	1	35.2	1	2	999	1	99	2	999	1	2	2	2	999	62	2	1	1	999	999	2	1	
573	20	1	1	1	11	1	1/8/2020	22:46	1	114	68	1	94	2	999	1	37.1	1	2	999	1	99	2	999	1	2	2	2	999	999	1	2	1	999	999	1	3	
574	28	1	3	3	39	1	1/25/2020	00:53	1	114	72	1	90	2	999	1	36.4	999	2		1	99	2	999	3	3	2	1	999	999	2	2	2	05:00	25/01/2020	1	3	2
575	28	1	3	3	37	2	1/9/2020	19:46	1	137	69	2	999	2	999	2	999	1	2	999	2	999	2	999	4	4	2	1	999	134	1	2	1	19:46	09/01/2020	2	2	
576	24	1	1	2	17	12	1/23/2020	18:09	1	106	66	1	109	2	999	1	35.5	2	2		1	99	2	999	2	2	8	1	999	999	2	2	1	999	999	1	3	
577	32	1	1	3	32	1	1/4/2020	05:37	2	999	999	2	999	2	999	2	999	2	2		2	999	2	999	3	3	8	1	999	999	1	2	1	999	04/01/2020	1	3	
578	27	1	1	3	38	1	1/25/2020	13:25	1	124	64	1	98	2	999	1	35.9	2	2		1	99	2	999	2	2	10	1	999	999	1	2	1	999	999	1	3	
579	21	1	1	3	38	1	1/22/2020	15:05	1	133	77	1	84	2	999	1	36.8	1	2	999	1	99	2	999	2	2	10	1	999	255	2	1	1	999	999	2	2	
580	21	999	1	3	38	1	1/23/2020	08:57	1	122	75	1	102	2	999	1	36.3	1	2	999	1	99	2	999	2	2	2	1	999	999	2	2	1	999	999	1	3	
581	21	1	1	3	39	1	1/28/2020	01:17	1	150	95	1	82	2	999	1	36.6	1	2	999	2	999	2	999	3	3	2	1	999	999	1	1	2	999	999	1	3	2
582	28	1	1	3	36	1	1/26/2020	02:39	1	140	90	2	999	2	999	1	36.4	2	2		2	999	2	999	999	0	0	3	N√O S	999	1	3	2	999	999	1	4	1
583	25	1	1	3	33	1	1/3/2020	10:06	1	111	73	1	71	2	999	1	36.1	1	2	999	1	99	2	999	2	2	2	1	999	144	2	1	1	999	03/01/2020	2	2	
584	25	1	1	3	35	1	1/15/2020	09:56	1	103	72	1	82	2	999	1	36.4	1	2	999	1	99	2	999	2	3	2	2	999	999	1	2	1	999	999	1	3	
585	22	1	3	2	24	1	1/10/2020	12:05	1	120	80	1	85	2	999	1	35.5	999	2		2	999	2	999	3	4	12	2	999	999	1	1	2	999	10/01/2020	1	3	4
586	22	1	3	3	30	1	1/31/2020	18:29	1	103	50	1	83	2	999	1	36.3	1	2	999	1	99	2	999	3	3	2	1	999	59	2	2	1	22:21	31/01/2020	2	2	
587	24	1	3	3	31	1	1/23/2020	15:21	1	102	66	1	81	2	999	1	35.6	1	2	999	1	100	2	999	2	2	2	1	999	999	1	1	1	19:40	23/01/2020	1	3	
588	28	2	4	3	37	1	1/9/2020	12:39	1	110	66	1	84	2	999	1	36.4	1	2		1	99	2	999	2	2	2	2	999	59	2	2	1	999	09/01/2020	2	1	
589	28	2	1	3	40	1	1/18/2020	07:41	1	125	82	1	100	2	999	1	35.6	1	2	999	1	100	2	999	1	2	2	2	999	999	2	2	1	999	999	1	3	
590	26	1	2	3	32	3	1/21/2020	11:31	1	140	80	1	81	2	999	1	36.4	1	2	999	2	999	2	999	999	0	0	3	N√O S	144	1	3	1	999	21/01/2020	2	4	
591	26	1	2	3	33	1	1/26/2020	14:08	1	133	66	1	88	2	999	1	36.8	2	2		1	99	2	999	2	2	6	1	999	127	2	1	1	999	999	2	2	
592	19	1	2	1	12	1	1/13/2020	07:05	1	96	62	1	85	2	999	1	36	1	1	4	1	99	2	999	3	3	2	1	999	120	2	1	999	999	999	2	2	
593	24	1	1	2	19	2	1/15/2020	10:07	1	99	58	1	77	2	999	1	35.6	999	2		1	100	2	999	1	2	1	2	999	999	2	2	1	999	999	1	3	
594	31	1	1	3	39	1	1/31/2020	10:03	1	126	78	1	103	2	999	1	36.3	2	2		1	99	2	999	1	2	12	2	999	37	1	2	1	999	999	2	1	
595	34	1	6	3	39	3	1/26/2020	12:13	1	130	80	1	92	2	999	2	999	1	2	999	2	999	2	999	999	0	0	3	N√O S	92	2	3	1	999	999	2	4	
596	35	1	1	3	40	1	1/2/2020	10:50	2	999	999	2	999	2	999	2	999	1	2	999	2	999	2	999	2	2	2	1	999	160	2	999	1	999	02/01/2020	2	2	

597	35	1	1	3	40	1	1/4/2020	10:30	1	132	91	1	89	2	999	1	35.9	2	2		1	98	2	999	3	3	2	1	999	100	2	2	2	999	04/01/2020	2	2	2
598	34	1	3	3	40	1	1/24/2020	00:48	1	110	65	1	67	2	999	1	36.3	999	2		1	99	2	999	3	3	2	1	999	999	2	1	2	999	999	1	3	1
599	33	1	3	3	36	1	1/28/2020	15:30	1	162	95	1	83	2	999	1	35.6	1	2	999	1	100	2	999	4	4	12	1	999	75	2	1	2	999	999	2	2	1
600	49	1	4	2	22	2	1/17/2020	19:52	1	127	64	1	70	2	999	1	36.6	2	2		1	99	2	999	2	2	10	1	999	999	2	1	1	999	999	1	3	
601	38	1	4	3	41	1	1/31/2020	16:54	1	105	62	1	73	2	999	1	35.4	2	2		1	99	2	999	2	2	12	1	999	999	1	1	2	999	999	1	3	2
602	30	1	2	3	39	4	1/6/2020	12:14	1	96	58	1	82	2	999	1	36.1	2	2		1	99	2	999	3	3	10	1	999	91	2	1	1	999	06/01/2020	2	2	
603	30	1	2	3	40	2	1/12/2020	08:50	1	120	63	2	999	2	999	2	999	2	2		2	999	2	999	1	2	12	2	999	999	1	2	2	999	999	1	3	1
604	30	1	2	3	39	2	1/2/2020	14:59	1	106	64	1	90	2	999	2	999	2	2		1	99	2	999	3	2	10	2	999	999	2	999	1	999	999	1	3	
605	25	1	3	2	18	1	1/15/2020	12:44	1	134	70	1	123	2	999	1	36	1	2	999	1	100	2	999	4	3	3	2	999	106	1	1	1	999	15/01/2020	2	2	
606	24	1	3	3	39	2	1/7/2020	10:47	1	117	67	1	105	2	999	1	35.9	2	2		1	99	2	999	1	1	12	1	999	223	1	1	2	999	07/01/2020	2	1	1
607	24	2	1	3	37	3	1/27/2020	19:23	1	120	90	1	88	2	999	2	999	1	2	999	2	999	2	999	2	3	2	2	999	97	1	2	1	22:05	27/01/2020	2	2	
608	28	1	1	3	40	1	1/12/2020	19:06	1	101	64	1	87	2	999	1	36.1	1	2	999	1	99	2	999	2	2	2	1	999	999	2	2	1	999	999	1	3	
609	28	1	1	3	41	1	1/15/2020	11:46	1	103	66	1	93	2	999	1	36.1	999	2		1	99	2	999	2	2	2	1	999	999	1	1	2	999	15/01/2020	1	3	1
610	36	1	2	2	24	1	1/4/2020	00:38	1	104	65	2	999	2	999	1	37.3	1	2	999	2	999	2	999	3	3	6	1	999	67	999	1	999	999	04/01/2020	2	2	
611	27	1	1	3	35	1	1/7/2020	14:50	1	122	76	1	95	2	999	1	36.3	1	1	5	1	100	2	999	3	3	2	1	999	100	2	2	999	999	999	1	2	
612	20	1	1	3	32	1	1/21/2020	13:36	1	107	72	1	89	2	999	1	35.4	1	2	999	1	100	2	999	2	2	2	1	999	999	1	2	1	999	999	1	3	
613	32	1	5	3	41	1	1/10/2020	08:43	1	94	62	1	104	2	999	1	36.1	1	2	999	1	99	2	999	2	2	2	1	999	189	2	1	2	999	999	3	2	1
614	32	1	5	3	39	1	1/8/2020	21:02	1	113	74	1	107	2	999	1	36.5	999	2		1	99	2	999	2	2	10	1	999	228	2	2	1	999	09/01/2020	2	2	
615	31	1	4	3	37	2	1/3/2020	13:27	1	103	46	1	85	2	999	2	999	1	1	6	1	99	2	999	3	3	2	1	999	33	2	1	999	999	999	2	2	
616	39	2	1	3	36	3	1/19/2020	19:39	1	158	105	1	96	2	999	1	36.4	1	2	999	1	99	2	999	4	4	3	1	999	201	2	1	999	999	20/01/2020	2	2	
617	39	2	1	3	35	1	1/5/2020	12:03	1	150	90	1	99	2	999	1	35.7	1	2	999	2	999	2	999	999	0	0	3	N√O S	57	2	3	1	999	05/01/2020	2	4	
618	39	2	1	3	36	3	1/21/2020	17:08	1	146	91	1	78	2	999	1	36.6	999	2		1	100	2	999	4	4	3	1	999	42	2	1	2	999	999	2	2	1
619	33	1	2	2	23	1	1/27/2020	20:23	2	999	999	2	999	2	999	2	999	1	2	999	2	999	2	999	2	2	2	1	999	999	1	1	1	999	999	1	3	
620	30	2	1	2	13	1	1/11/2020	22:35	1	120	75	1	69	2	999	1	36.8	1	2	999	1	99	2	999	2	2	2	1	999	999	2	1	999	999	999	1	3	
621	24	1	1	1	8	1	1/10/2020	12:02	1	107	68	1	95	2	999	1	36.7	1	2	999	2	999	2	999	2	2	2	1	999	266	2	999	4	16:28	10/01/2020	3	2	
622	31	1	5	2	25	7	1/23/2020	15:03	1	105	58	1	94	2	999	1	35.9	1	2	999	1	99	2	999	2	2	2	1	999	999	2	1	3	22:00	23/01/2020	1	3	
623	28	1	2	1	12	1	1/10/2020	13:02	1	114	75	1	78	2	999	1	36.7	1	2	999	1	98	2	999	3	2	2	2	999	999	1	1	1	999	999	1	3	
624	32	1	1	1	9	1	1/23/2020	11:05	1	91	60	1	64	2	999	2	999	1	2	999	2	999	2	999	999	0	0	3	999	105	2	3	1	13:10	23/01/2020	2	4	
625	21	2	1	3	40	1	1/20/2020	02:17	1	117	76	1	82	2	999	2	999	2	2		2	999	2	999	999	0	0	3	N√O S	999	2	3	999	999	999	1	4	2
626	29	1	3	3	31	3	1/10/2020	21:15	1	106	71	1	83	2	999	1	36.5	1	2	999	2	999	2	999	2	2	2	1	999	150	1	2	1	02:25	11/01/2020	2	2	
627	33	1	1	1	9	1	1/17/2020	13:13	1	103	52	1	62	2	999	1	36.1	1	2	999	1	100	2	999	3	3	2	1	999	999	2	2	999	999	999	1	3	
628	21	1	1	3	40	1	1/20/2020	15:13	1	113	57	1	102	2	999	1	35.9	1	2	999	1	99	2	999	2	2	2	1	999	72	1	2	1	999	999	2	1	
629	21	1	1	3	41	1	1/26/2020	13:58	1	101	55	1	79	2	999	1	36.3	1	2	999	1	99	2	999	2	2	2	1	999	82	2	1	1	999	999	2	1	
630	30	1	1	3	39	1	1/21/2020	09:12	1	123	84	1	99	2	999	1	34.9	2	2		1	99	2	999	2	2	10	1	999	48	2	2	1	999	21/01/2020	2	1	
631	30	1	1	3	40	1	1/24/2020	08:54	1	124	80	1	98	2	999	1	35.8	1	2	999	1	100	2	999	3	2	3	2	999	306	1	2	1	999	999	2	2	
632	30	1	1	3	40	1	1/28/2020	08:18	1	143	92	1	103	2	999	1	36.2	1	2		2	999	2	999	4	4	3	1	999	102	2	2	2	999	999	2	2	1
633	41	2	2	3	37	2	1/14/2020	19:46	1	128	77	1	85	2	999	1	36.6	2	2		1	99	2	999	2	2	2	1	999	74	1	2	1	22:40	14/01/2020	2	1	
634	31	1	3	3	37	11	1/22/2020	21:16	1	110	72	1	84	2	999	1	36.6	2	2		2	999	2	999	1	2	5	2	999	999	2	2	1	999	999	1	3	
635	25	1	2	3	35	2	1/14/2020	07:22	1	122	64	1	75	2	999	2	999	2	2		1	99	2	999	1	2	12	2	999	8	1	2	1	999	999	2	1	
636	36	1	1	2	21	1	1/31/2020	15:52	1	90	53	1	77	2	999	1	35.9	1	2	999	1	99	2	999	2	2	6	1	999	128	2	1	1	20:00	31/01/2020	2	2	
637	29	2	2	1	5	12	1/31/2020	21:00	1	105	60	1	74	2	999	1	37	1	2	999	2	999	2	999	2	2	12	1	999	999	2	1	1	999	31/01/2020	1	3	
638	26	1	2	3	39	1	1/14/2020	22:55	1	111	65	1	75	2	999	1	36.1	1	2	999	1	99	2	999	2	2	6	1	999	999	1	1	1	999	999	1	3	
639	26	1	2	3	40	1	1/16/2020	01:16	1	103	62	1	75	2	999	2	999	1	2	999	2	999	2	999	3	3	2	1	999	74	1	2	1	999	999	2	2	
640	26	1	2	3	40	1	1/17/2020	13:49	1	102	60	1	78	2	999	1	35.6	999	2		1	99	2	999	3	4	6	2	999	999	1	2	2	999	999	1	3	2
641	43	2	4	1	12	1	1/27/2020	22:08	1	130	80	1	88	2	999	1	36.5	999	2		2	999	2	999	3	2	8	2	999	402	2	1	1	09:30	28/01/2020	2	2	
642	24	1	3	2	15	9	1/13/2020	14:37	1	106	60	1	76	2	999	1	37.1	999	2		1	99	2	999	1	2	12	2	999	263	2	999	4	19:50	13/01/2020	3	2	
643	24	1	3	2	16	9	1/14/2020	09:41	1	116	66	1	82	2	999	1	35.4	1	2	999	1	99	2	999	2	2	9	1	999	167	1	1	1	999	999	2	2	
644	24	1	3	2	16	9	1/17/2020	11:24	1	109	66	1	75	2	999	1	36.4	1	2	999	1	99	2	999	2	2	9	1	999	999	1	1	1	999	999	1	3	
645	20	1	1	3	39	1	1/3/2020	13:34	1	108	59	1	97	2	999	2	999	1	1	10	1	99	2	999	4	4	2	1	999	999	1	1	2	14:15	03/01/2020	1	3	2
646	34	1	1	1	12	1	1/8/2020	09:50	1	119	67	1	65	2	999	1	36.3	1	2	999	1	100	2	999	2	2	8	1	999	999	1	1	2	999	08/01/2020	1	3	3
647	19	1	2	3	32	1	1/8/2020	12:34	1	100	80	2	999	2	999	1	35.6	1	2	999	2	999	2	999	999	0	0	3	N√O S	131	1	3	1	999	08/01/2020	2	4	
648	24	1	2	3	38	1	1/2/2020	03:27	1	129	75	1	94	2	999	1	36.3	2	2		2	999	2	999	999	0	0	3	N√O S	133	2	3	1	09:30	02/01/2020	3	4	
649	24	1	2	3	38	1	1/2/2020	16:07	1	115	76	1	132	2	999	2	999	1	1	8	1	99	2	999	4	4	2	1	999	83	1	1	1	18:00	02/01/2020	2	2	
650	24	1	2	3	38	1	1/3/2020	03:39	1	140	90	1	128	2	999	1	37.1	2	2		2	999	2	999	999	0	0	3	N√O S	999	2	3	2	999	03/01/2020	1	4	2
651	21	1	1	1	11	1	1/12/2020	17:49	1	116	67	1	107	2	999	1	37	999	2		1	99	2	999	2	2	8	1	999	999	1	1	1	999	999	1	3	
652	20	1	1	2	14	4	1/3/2020	09:11	1	122	80	1	94	2	999	1	35.3	1	1	3	1	99	2	999	2	2	2	1	999	154	2	999	4	12:45	03/01/2020	3	2	
653	24	1	3	3	28	1	1/12/2020	15:45	1	106	55	1	95	2	999	1	35.4	1	2	999	1	99	2	999	2	2	2	1	999	999	2	999	1	20:50	12/01/2020	1	3	
654	23	1	1	1	9	1	1/10/2020	11:43	1	91	53	1	80	2	999	2	999	1	2	999	1	99	2	999	1	2	2	2	999	385	2	999	4	16:28	10/01/2020	3	2	
655	36	1	3	3	41	1	1/2/2020	12:05	1	101	56	2	999	2	999	2	999	1	2	999	2	999	2	999	3	3	2	1	999	999	2	2	1	999	02/01/2020	1	3	
656	22	1	1	3	37	2	1/13/2020	05:41	1	124	68	1	78	2	999	1	35.6	1	2	999	1	99	1	96	1	2	2	2	999	214	1	2	1	999	13/01/2020	2	2	
657	22	1	1	3	39	2	1/30/2020	14:19	1	122	77	1	84	2	999	1	36.1	1	2	999	1	99	2	999	1	2	2	2	999	221	2	1	1	999	999	2	2	
658	24	1	1	2	21	1	1/22/2020	13:16	1	107	50	1	85	2	999	1	35.6	2	2		1	99	2	999	1	2	10	2	999	999	1	2	1	999	999	1	3	
659	27	1	1	1	11	1	1/12/2020	15:40	1	124	63	1	115	2	999	1	38.1	1	2	999	1	99	2	999	3	3	5	1	999	70	1	1	1	999	999	2	2	
660	999	999	1	1	11	1	1/13/2020	999	1	104	58	1	100	2	999	1	36.4	2	2		1	99	2	999	3	3	6	1	999	999	2	999	4	11:30	13/01/2020	1	3	
661	23	1	1	3	40	1	1/31/2020	06:29	1	114	70	1	78	2	999	1	36.2	2	2		1	99	2	999	2	3	2	2	999	121	1	2	3	14:30	31/01/2020	2	2	
662	25	1	2	2	21	1	1/15/2020	18:28	1	110	55	1	73	2	999	2	999	1	2	999	2	999	2	999	3	3	2	1	999	58	2	1	1	21:20	15/01/2020	2	2	
663	37	2	2	3	40	1	1/26/2020	10:11	1	127	76	1	73	2	999	1	36.6	1	2	999	1	99	2	999	2	2	2	1	999	49	2	1	1	14:30	26/01/2020	2	1	
664	33	1	3	1	5	1	1/21/2020	13:37	1	125	80	1	122	2	999	1	36.7	1	2	999	1	99	2	999	2	2	9	1	999	999	2	1	1	999	999	1	3	

665	33	1	3	1	5	1	1/21/2020	01:31	1	136	92	1	123	2	999	1	36.8	1	2	999	1	99	2	999	2	2	9	1	999	254	2	3	3	05:45	21/01/2020	3	2	
666	22	1	1	3	40	1	1/28/2020	05:37	1	141	84	1	70	2	999	1	36.2	999	2		2	999	2	999	999	0	0	3	N√O S	58	1	3	1	999	999	2	4	
667	23	1	1	3	38	2	1/6/2020	14:19	1	113	78	1	113	2	999	1	36.7	2	2		1	99	1	231	1	4	12	2	999	999	2	2	2	999	999	1	3	1
668	23	1	1	3	38	2	1/4/2020	12:35	1	130	90	1	102	2	999	1	36.6	1	2	999	1	99	2	999	2	3	2	2	999	999	2	2	999	999	04/01/2020	1	3	
669	37	1	2	3	38	4	1/30/2020	02:30	1	135	97	1	86	2	999	1	35	999	2		2	999	2	999	999	0	0	3	N√O S	999	1	3	2	999	999	1	4	999
670	32	1	1	1	5	1	1/5/2020	11:18	1	122	73	1	79	2	999	1	36.3	1	1	4	1	100	2	999	3	3	2	1	999	999	2	1	1	999	05/01/2020	1	3	
671	25	1	2	3	32	1	1/10/2020	11:55	1	108	62	1	119	1	37	2	999	1	2	999	1	99	2	999	2	2	6	1	999	55	1	1	1	13:30	11/01/2020	2	1	
672	31	1	3	3	41	1	1/20/2020	08:59	1	139	82	1	94	2	999	1	35.7	1	2	999	1	99	2	999	3	3	2	1	999	63	1	2	2	999	999	2	2	2
673	25	2	1	1	9	1	1/24/2020	08:44	1	113	76	1	90	2	999	1	36.1	2	2		1	100	2	999	1	2	2	2	999	999	1	2	1	999	999	1	3	
674	37	1	2	999	999	1	1/13/2020	16:11	1	99	57	1	73	2	999	1	35.9	1	2	999	1	99	2	999	2	2	2	1	999	999	1	2	1	999	999	1	3	
675	37	1	2	1	10	1	1/9/2020	16:17	1	101	61	1	85	2	999	1	36.2	1	2	999	1	99	2	999	2	3	8	2	999	999	1	2	1	999	999	1	3	
676	36	1	3	3	39	1	1/9/2020	15:04	1	104	62	1	87	2	999	2	999	999	2		1	99	2	999	3	2	10	2	999	76	2	2	2	999	999	2	1	1
677	36	1	3	2	24	1	1/23/2020	11:37	1	98	66	1	109	2	999	1	36.1	1	2	999	1	99	2	999	2	2	2	1	999	999	1	2	1	999	999	1	3	
678	37	1	3	3	36	1	1/27/2020	07:57	1	112	69	1	90	2	999	1	35.9	1	2	999	1	99	2	999	3	3	10	1	999	999	1	1	2	999	999	1	3	2
679	23	2	3	3	33	1	1/18/2020	15:36	1	118	70	1	109	2	999	1	36.3	1	2	999	1	100	2	999	2	2	2	1	999	999	2	2	1	999	999	1	3	
680	20	1	1	2	16	1	1/3/2020	09:50	1	107	69	1	83	2	999	1	36.4	1	1	5	1	99	2	999	3	3	2	1	999	999	2	1	1	999	03/01/2020	1	3	
681	34	1	4	3	39	1	1/3/2020	05:25	1	104	61	1	64	2	999	1	35.4	1	2	999	1	99	2	999	3	3	10	1	999	95	2	2	1	999	03/01/2020	2	2	
682	41	1	3	1	10	4	1/27/2020	12:42	1	134	95	1	90	2	999	1	36.8	2	2		2	999	2	999	999	0	0	3	N√O S	999	1	3	1	999	999	1	4	
683	27	1	2	1	8	1	1/29/2020	07:30	1	111	60	1	117	2	999	1	36.7	1	2	999	1	100	2	999	2	2	8	1	999	999	1	1	1	999	999	1	3	
684	34	1	4	3	40	1	1/10/2020	11:36	1	99	61	1	74	2	999	1	35.4	999	2		1	99	2	999	3	3	10	1	999	999	2	2	2	999	10/01/2020	1	3	1
685	19	1	1	3	32	1	1/2/2020	17:55	1	94	52	1	95	2	999	2	999	2	2		1	100	2	999	1	1	5	1	999	999	2	999	1	999	02/01/2020	1	3	
686	20	1	2	1	6	1	1/4/2020	12:28	1	108	53	1	78	2	999	1	36.7	1	2	999	1	99	2	999	2	2	2	1	999	999	2	1	1	999	04/01/2020	1	3	
687	20	1	2	1	8	1	1/23/2020	19:05	1	106	62	1	88	2	999	1	36.7	1	2	999	1	100	2	999	2	2	6	1	999	100	1	1	1	999	999	2	1	
688	24	2	1	1	5	1	1/2/2020	11:38	1	107	69	1	77	2	999	2	999	1	2	999	2	999	2	999	3	3	2	1	999	999	1	2	1	999	02/01/2020	1	3	
689	24	2	1	1	6	1	1/6/2020	09:22	1	113	69	1	73	2	999	1	35.4	1	2	999	1	100	2	999	2	2	2	1	999	175	1	1	1	999	06/01/2020	2	2	
690	34	999	3	3	35	1	1/15/2020	03:15	1	130	80	1	88	2	999	1	36.5	999	2		2	999	2	999	999	0	0	3	N√O S	999	2	3	2	999	999	1	4	2
691	21	1	1	3	37	1	1/2/2020	14:06	1	130	78	1	95	2	999	2	999	2	2		1	99	2	999	1	1	12	1	999	96	2	999	1	999	02/01/2020	2	1	
692	38	1	3	2	15	1	1/27/2020	10:01	1	119	67	1	102	2	999	1	36.1	1	2	999	1	99	2	999	3	3	8	1	999	999	1	1	999	999	999	1	3	
693	38	999	1	1	4	1	1/13/2020	09:59	1	113	65	1	91	2	999	1	36.1	2	2		1	99	2	999	1	2	12	2	999	152	2	2	1	999	999	2	2	
694	32	999	2	3	37	1	1/24/2020	999	1	115	53	2	999	2	999	2	999	999	2		2	999	2	999	999	0	0	3	N√O S	999	2	3	2	999	999	1	4	2
695	24	1	3	1	7	1	1/9/2020	12:34	1	130	65	1	76	2	999	1	36.8	1	2	999	1	99	2	999	2	3	2	2	999	999	2	2	1	999	999	1	3	
696	24	1	3	1	7	1	1/16/2020	19:51	1	109	62	1	78	2	999	2	999	1	2	999	2	999	2	999	2	2	2	1	999	999	2	2	1	999	999	1	3	
697	23	1	1	3	38	1	1/9/2020	11:54	1	126	73	1	94	2	999	1	36.7	999	2		1	99	2	999	1	3	10	2	999	999	2	2	2	19:00	09/01/2020	1	3	2
698	42	1	4	2	22	2	1/6/2020	01:13	1	141	74	1	91	2	999	1	36.2	1	2	999	1	99	2	999	4	3	3	2	999	87	2	2	1	03:45	06/01/2020	2	2	
699	42	1	4	2	24	2	1/23/2020	08:58	1	129	72	1	80	2	999	1	36.3	1	2	999	1	99	2	999	2	2	2	1	999	999	2	2	2	999	999	1	3	4
700	42	1	4	2	25	3	1/27/2020	06:38	1	103	63	1	92	2	999	1	36.3	2	2		1	99	1	114	1	1	12	1	999	52	1	2	2	14:00	27/01/2020	2	1	4
701	29	2	1	3	38	3	1/3/2020	01:45	1	146	91	1	72	2	999	1	36.2	2	2		1	100	2	999	3	3	2	1	999	999	1	1	2	999	03/01/2020	1	3	1
702	25	1	1	3	39	1	1/25/2020	03:59	1	112	68	1	75	2	999	1	35.1	2	2		2	999	2	999	999	0	0	3	999	999	2	3	999	999	999	1	4	2
703	21	1	1	3	40	3	1/19/2020	06:02	1	130	80	1	98	2	999	2	999	1	2	999	2	999	2	999	2	2	2	1	999	999	1	1	2	999	19/01/2020	1	3	2
704	21	1	1	3	38	3	1/11/2020	17:37	1	119	74	1	108	2	999	1	36.1	1	2	999	1	99	2	999	3	3	10	1	999	999	1	2	1	999	999	1	3	
705	41	1	2	3	40	1	1/24/2020	04:05	1	90	60	1	95	2	999	1	36	999	2		2	999	2	999	999	0	0	3	N√O S	999	1	3	1	08:55	24/01/2020	1	4	
706	32	1	3	3	32	12	1/15/2020	12:12	1	84	47	1	59	2	999	1	35.9	1	2	999	1	99	2	999	2	2	8	1	999	999	1	1	1	999	15/01/2020	1	3	
707	23	1	1	3	38	6	1/28/2020	16:01	1	141	91	1	75	2	999	1	36.8	2	2		1	100	2	999	3	3	12	1	999	999	2	1	1	22:40	28/01/2020	1	3	
708	19	1	4	3	40	1	1/5/2020	15:25	1	116	67	1	97	2	999	1	36.5	1	2	999	1	99	2	999	3	3	2	1	999	999	1	1	2	999	05/01/2020	1	3	2
709	30	2	1	3	34	1	1/4/2020	17/20	1	107	68	1	95	2	999	1	37.4	2	2		1	99	2	999	2	2	7	1	999	95	2	1	1	999	04/01/2020	2	1	
710	26	1	2	3	39	1	1/11/2020	14:31	1	117	70	1	95	2	999	2	999	999	2		1	99	2	999	1	2	12	2	999	29	1	2	1	999	999	2	1	
711	26	1	2	3	40	1	1/12/2020	11:29	1	127	70	1	92	2	999	2	999	999	2		1	99	2	999	3	3	7	2	999	999	2	2	2	999	999	1	3	2
712	23	1	1	1	11	1	1/11/2020	14:46	1	94	58	1	81	2	999	1	36.3	1	2	999	1	100	2	999	3	3	2	1	999	94	1	2	999	999	999	2	2	
713	23	2	3	1	7	1	1/9/2020	08:28	1	119	66	1	90	2	999	1	36.4	2	2		1	99	2	999	2	2	8	1	999	999	2	2	1	999	999	1	3	
714	23	2	2	1	9	1	1/16/2020	10:20	1	117	69	1	87	2	999	1	36.3	1	2	999	1	99	2	999	2	2	2	1	999	999	1	1	2	999	999	1	3	999
715	23	2	2	1	8	1	1/18/2020	08:33	1	116	70	1	84	2	999	1	36.3	1	2	999	1	99	2	999	1	2	2	2	999	999	2	2	2	999	999	1	3	3
716	23	2	2	1	6	1	1/2/2020	11:31	1	120	84	2	999	2	999	1	35.3	1	2	999	2	999	2	999	4	4	2	1	999	999	2	2	1	999	02/01/2020	1	3	
717	34	2	2	3	35	1	1/18/2020	00:48	1	115	57	1	78	2	999	1	36.7	999	2		1	99	2	999	1	2	2	2	999	999	2	3	4	999	999	1	3	
718	33	1	2	1	7	1	1/29/2020	19:46	1	102	66	1	76	2	999	1	35.9	2	2		1	100	2	999	2	2	8	1	999	999	2	2	1	999	999	1	3	
719	30	1	2	3	33	1	1/15/2020	11:13	1	126	76	1	83	2	999	1	36.7	1	2	999	1	99	2	999	2	2	2	1	999	355	2	999	4	17:05	15/01/2020	3	2	
720	30	1	2	3	34	3	1/31/2020	16:20	1	130	71	1	76	2	999	1	36.8	2	2		1	99	2	999	1	2	12	2	999	90	1	2	1	22:00	31/01/2020	2	1	
721	26	3	3	2	14	1	1/2/2020	16:02	1	88	54	1	70	2	999	2	999	1	1	9	1	99	2	999	4	4	2	1	999	103	2	1	1	20:45	02/01/2020	2	2	
722	31	1	3	3	41	12	1/6/2020	08:17	1	114	52	1	97	2	999	1	36.3	2	2		1	99	2	999	2	2	12	1	999	999	2	1	2	999	06/01/2020	1	3	2
723	35	1	2	3	29	1	1/17/2020	20:36	1	112	73	1	86	2	999	1	37	999	2		1	99	2	999	2	2	2	1	999	999	2	2	999	999	999	1	3	
724	31	1	2	3	38	1	1/17/2020	17:28	1	110	74	1	85	2	999	1	36.4	1	2	999	1	99	2	999	3	3	2	1	999	999	1	2	1	999	999	1	3	
725	31	1	2	3	40	1	1/27/2020	10:59	1	116	81	1	97	2	999	1	34.5	1	2	999	1	99	2	999	1	2	2	2	999	181	1	2	1	999	999	2	2	
726	28	1	3	3	34	1	1/13/2020	16:19	1	94	66	1	83	2	999	1	35.5	1	1	6	1	99	2	999	3	3	2	1	999	999	2	2	1	999	999	1	3	
727	19	1	1	3	39	1	1/19/2020	18:32	1	103	66	1	94	2	999	1	35.9	999	2		1	100	2	999	3	3	2	1	999	78	1	1	2	999	999	2	2	2
728	23	1	1	3	38	1	1/6/2020	09:15	1	101	63	1	84	2	999	1	35.2	1	2	999	1	99	2	999	2	2	2	1	999	95	2	999	4	10:50	06/01/2020	3	1	
729	37	1	1	2	16	4	1/24/2020	12:00	1	108	66	1	71	2	999	1	35.9	1	2	999	1	99	2	999	2	2	9	1	999	140	1	1	1	999	999	2	2	
730	23	1	2	3	32	1	1/11/2020	14:27	1	99	65	1	104	2	999	1	36.6	1	2	999	1	99	2	999	1	2	2	2	999	999	1	2	1	999	999	1	3	
731	33	1	6	1	10	7	1/14/2020	21:36	1	101	58	1	79	2	999	1	36.8	1	2	999	1	99	2	999	4	2	8	2	999	999	2	2	1	999	999	1	3	
732	18	999	2	1	10	1	1/16/2020	10:38	1	109	64	1	75	2	999	1	36.1	1	2	999	1	99	2	999	999	0	0	3	N√O S	999	1	3	1	999	999	1	4	

733	32	1	2	3	40	1	1/12/2020	03:56	1	140	90	1	83	2	999	1	36.2	999	2		2	999	2	999	999	0	0	3	N√O S	999	2	3	2	999	999	1	4	2
734	28	2	2	3	34	1	1/15/2020	10:43	1	117	79	1	97	2	999	1	36.4	2	2		1	98	2	999	3	3	7	1	999	999	2	1	2	999	15/01/2020	1	3	1
735	25	2	1	3	33	12	1/10/2020	20:55	1	126	76	1	93	2	999	2	999	999	2		2	999	2	999	3	2	10	2	999	115	2	2	1	999	10/01/2020	2	1	
736	25	2	1	3	33	1	1/12/2020	14:53	1	127	74	1	91	2	999	1	36.4	999	2		1	99	2	999	3	3	10	1	999	67	1	1	1	999	999	2	2	
737	25	999	1	3	33	12	1/14/2020	999	1	129	77	1	116	2	999	1	38.3	999	2		1	99	2	999	3	3	5	1	999	999	2	1	2	999	999	1	3	1
738	23	1	1	1	8	1	1/17/2020	08:57	1	122	61	1	99	2	999	1	36.7	2	2		1	99	2	999	2	2	8	1	999	999	2	2	1	999	999	1	3	
739	33	1	2	3	37	1	1/27/2020	13:41	1	118	74	1	132	2	999	1	36.7	2	2		1	100	2	999	2	2	12	1	999	209	2	1	1	999	999	2	2	
740	46	1	3	1	11	999	1/15/2020	09:16	1	117	67	1	66	2	999	1	36.4	1	2	999	1	99	2	999	2	2	2	1	999	999	1	2	1	999	15/01/2020	1	3	
741	29	1	2	1	7	1	1/9/2020	15:19	1	111	71	1	68	2	999	1	36.3	1	2	999	1	100	2	999	3	3	2	1	999	56	1	2	999	999	999	2	2	
742	22	1	3	3	37	1	1/6/2020	23:44	1	118	66	2	999	2	999	1	37	1	2	999	2	999	2	999	4	4	2	1	999	999	2	2	2	999	999	1	3	2
743	22	999	2	3	36	1	1/19/2020	09:34	1	107	69	1	84	2	999	1	35.9	1	2	999	1	99	2	999	1	2	2	2	999	151	1	2	1	999	19/01/2020	2	2	
744	38	1	7	2	25	1	1/12/2020	22:38	1	107	65	1	96	2	999	1	36.1	1	2	999	1	100	2	999	4	3	2	2	999	47	2	2	1	07:15	13/01/2020	2	2	
745	30	1	3	2	20	1	1/24/2020	14:05	1	119	74	1	90	2	999	1	36.1	1	2	999	1	99	2	999	3	3	2	1	999	45	1	1	3	19:34	24/01/2020	2	2	
746	27	1	3	3	40	2	1/4/2020	08:31	1	118	70	1	78	2	999	1	36.6	2	2		1	99	2	999	1	2	12	2	999	999	1	2	2	999	04/01/2020	1	3	1
747	39	1	2	2	14	1	1/30/2020	15:52	1	110	67	1	79	2	999	1	36.8	2	2		1	99	2	999	1	2	12	2	999	96	2	2	1	999	999	2	1	
748	20	1	4	2	21	1	1/8/2020	16:44	1	87	53	1	95	2	999	2	999	1	2	999	1	98	2	999	4	4	2	1	999	116	1	2	1	999	20:00	2	2	
749	29	2	2	3	31	1	1/25/2020	21:26	1	100	70	1	72	2	999	1	36.7	999	2		2	999	2	999	4	4	2	1	999	64	2	1	1	01:40	26/01/2020	2	2	
750	25	2	1	3	34	1	1/3/2020	11:30	1	114	71	1	89	2	999	1	36	1	2	999	1	100	2	999	3	3	2	1	999	85	2	1	1	17:00	03/01/2020	2	2	
751	29	3	1	1	11	1	1/11/2020	17:59	1	129	75	2	999	2	999	1	36.8	1	2	999	1	99	2	999	2	2	2	1	999	256	2	999	4	21:45	11/01/2020	3	2	
752	24	1	3	1	7	1	1/14/2020	12:32	1	99	55	1	75	2	999	1	36.4	1	2	999	1	99	2	999	4	2	8	2	999	999	2	1	1	999	999	1	3	
753	31	1	4	3	34	8	1/27/2020	11:13	1	92	52	1	83	2	999	1	34.5	2	2		1	100	2	999	3	3	7	1	999	72	2	2	1	999	999	2	2	
754	33	1	2	3	38	1	1/3/2020	13:39	1	112	53	1	78	2	999	2	999	1	1	8	1	99	2	999	4	4	2	1	999	999	2	2	2	999	03/01/2020	1	3	2
755	40	1	2	3	38	1	1/8/2020	12:16	1	110	80	2	999	2	999	1	35.9	999	2		2	999	2	999	999	0	0	3	N√O S	94	1	3	1	999	08/01/2020	2	4	
756	40	1	3	3	38	1	1/13/2020	01:47	1	129	77	1	84	2	999	1	36.5	999	2		2	999	2	999	999	0	0	3	N√O S	999	1	3	1	999	999	1	4	
757	40	1	2	3	38	999	1/15/2020	04:45	1	131	85	1	86	2	999	1	36	999	2		2	999	2	999	999	0	0	3	N√O S	999	1	3	2	999	999	1	4	2
758	39	1	1	2	16	1	1/6/2020	13:26	1	116	77	1	81	2	999	1	35.4	1	2	999	1	99	2	999	2	2	2	1	999	299	2	999	4	19:25	06/01/2020	3	2	
759	40	1	2	3	38	1	1/9/2020	02:55	1	124	80	1	88	2	999	1	35.5	999	2		2	999	2	999	2	3	2	2	999	45	2	2	1	09:30	09/01/2020	2	2	
760	23	2	2	3	36	1	1/17/2020	22:13	1	103	61	1	100	2	999	1	35.6	999	2		1	99	2	999	2	2	2	1	999	999	1	2	1	999	999	1	3	
761	19	2	2	1	11	1	1/14/2020	14:04	1	116	68	1	94	2	999	1	36.6	1	2	999	1	99	2	999	2	2	3	1	999	999	1	2	1	999	999	1	3	
762	29	999	2	3	40	1		999	1	117	68	1	96	2	999	1	35.8	1	2	999	1	98	2	999	2	2	2	1	999	999	2	999	4	13:50	19/01/2020	1	3	
763	29	1	1	3	38	1	43832	04:59	1	104	68	1	95	2	999	1	36.4	2	2		2	999	2	999	999	0	0	3	N√O S	999	1	3	1	999	02/01/2020	1	4	
764	29	1	1	3	39	11	43844	11:57	1	120	69	1	112	2	999	1	36.1	999	2		1	99	2	999	4	4	7	1	999	63	1	2	1	999	999	2	2	
765	25	1	1	1	7	12	43852	12:31	1	118	71	1	87	2	999	1	36.3	1	2	999	1	99	2	999	2	2	9	1	999	999	1	1	1	18:30	22/01/2020	1	3	
766	30	2	1	3	38	1	43832	23:09	1	130	77	1	87	2	999	1	35.5	2	2		1	100	2	999	2	2	12	1	999	201	2	999	2	999	999	2	2	1
767	35	1	2	1	8	1	43848	01:26	1	100	60	1	74	2	999	2	999	2	2		2	999	2	999	2	2	8	1	999	999	2	1	1	999	999	1	3	
768	35	1	2	1	8	1	43850	09:48	1	98	62	1	80	2	999	1	35.8	999	2		1	99	2	999	4	3	8	2	999	39	2	999	4	11:25	20/01/2020	3	2	
769	36	2	2	2	25	3	43853	17:45	1	94	57	1	109	2	999	1	37	999	2		2	999	2	999	999	0	0	3	N√O S	245	1	3	2	999	999	2	4	2
770	33	3	3	1	8	12	43844	16:05	1	124	63	1	102	2	999	1	37.8	1	2		1	99	2	999	2	3	5	2	999	235	1	2	1	999	999	2	2	
771	31	1	1	2	19	1	43858	17:01	1	125	69	1	93	2	999	1	36.3	1	2	999	1	99	2	999	2	2	3	1	999	999	1	2	1	999	999	1	3	
772	28	1	2	3	38	12	43843	07:27	1	130	85	1	91	2	999	1	36.4	2	2		1	100	2	999	1	2	12	2	999	999	2	2	2	999	999	1	3	1
773	33	1	1	3	33	1	43856	11:11	1	114	63	1	114	2	999	1	36.3	1	2	999	1	99	2	999	2	2	2	1	999	999	2	1	999	999	999	1	3	
774	27	1	5	2	17	12	43834	11:32	1	106	71	1	98	2	999	1	36.3	1	2	999	1	99	2	999	2	2	2	1	999	999	2	2	999	999	999	1	3	
775	30	1	999	999	999	1	43840	19:37	1	120	60	1	83	2	999	2	999	1	2	999	2	999	2	999	1	1	2	1	999	243	2	999	4	23:40	10/01/2020	3	2	
776	29	1	6	3	35	1	43836	10:55	1	99	65	1	95	2	999	1	35.8	1	2	999	1	100	2	999	4	4	2	1	999	50	2	2	999	999	999	2	2	
777	32	1	2	3	38	1	43859	07:13	1	113	77	1	99	2	999	1	36.3	2	2		1	100	2	999	3	3	7	1	999	999	1	1	2	999	999	1	3	2
778	32	1	2	3	37	1	43858	19:51	1	111	70	1	119	2	999	1	36.3	1	2	999	1	99	2	999	3	3	10	1	999	101	1	2	1	999	999	2	2	
779	35	1	3	1	5	1	43853	21:03	1	131	77	1	78	2	999	1	37.3	2	2		1	99	2	999	2	2	8	1	999	999	2	1	1	999	999	1	3	
780	31	1	1	2	20	1	43859	10:46	1	100	58	1	88	2	999	1	36.3	1	2	999	1	99	2	999	2	2	8	1	999	999	1	1	1	999	999	1	3	
781	28	1	1	1	6	1	43849	20:04	1	104	65	1	97	2	999	1	36.7	1	2	999	1	99	2	999	2	2	8	1	999	999	2	1	1	999	999	1	3	
782	34	1	999	999	999	11	43841	09:40	1	96	65	1	89	2	999	2	999	1	2	999	1	99	2	999	4	4	2	1	999	65	1	1	2	999	11/01/2020	2	2	999
783	25	1	1	3	40	1	43843	03:41	1	132	84	1	99	2	999	1	35.6	999	2		2	999	2	999	999	0	0	3	N√O S	999	1	3	999	999	999	1	4	2
784	27	1	1	2	15	1	43838	14:34	1	115	59	2	999	2	999	2	999	1	2	999	2	999	2	999	2	3	2	2	999	999	1	2	1	999	08/01/2020	1	3	
785	29	1	1	3	40	1	43835	09:31	1	120	70	1	85	2	999	1	36.4	1	2	999	1	99	2	999	3	3	2	1	999	999	2	2	2	999	05/01/2020	1	3	2
786	29	1	1	3	40	1	43835	04:14	1	120	80	2	999	2	999	2	999	1	2	999	2	999	2	999	2	2	2	1	999	44	2	2	1	999	05/01/2020	2	1	
787	21	1	1	3	28	1	43854	19:27	1	114	52	1	131	2	999	1	37.6	1	2	999	1	99	2	999	2	2	9	1	999	999	1	1	2	22:30	24/01/2020	1	3	999
788	34	1	1	3	37	2	43858	17:11	1	99	66	1	77	2	999	1	36.3	1	2	999	1	99	2	999	2	2	2	1	999	189	1	2	1	999	999	2	2	
789	20	1	1	3	37	1	43839	09:15	1	104	57	1	82	2	999	1	36.3	2	2		1	99	2	999	2	2	12	1	999	999	2	1	1	999	09/01/2020	1	3	
790	20	1	1	3	40	1	43859	18:59	1	117	77	1	98	2	999	1	36.9	2	2		1	99	2	999	2	2	2	1	999	121	1	1	1	999	999	2	2	
791	20	1	1	3	40	1	43860	02:08	1	131	71	1	103	2	999	2	999	2	2		1	99	2	999	3	3	2	1	999	999	1	1	2	999	999	1	3	2
792	19	1	1	3	41	1	43859	00:43	1	130	69	1	74	2	999	1	35.7	1	2	999	1	99	2	999	2	3	2	2	999	999	1	1	2	08:40	28/01/2020	1	3	2
793	27	2	2	3	38	1	43845	20:06	1	123	57	1	84	2	999	1	36.7	1	2	999	1	99	2	999	1	2	2	2	999	999	2	2	1	999	999	1	3	
794	27	2	2	3	40	1	43860	18:30	1	123	69	1	89	2	999	1	35.2	2	2		1	100	2	999	1	1	12	1	999	140	2	2	1	999	999	2	1	
795	25	1	4	2	16	12	43858	13:51	1	103	56	1	78	2	999	1	37	1	2	999	1	100	2	999	2	2	6	1	999	164	2	1	1	19:33	28/01/2020	2	2	
796	37	1	3	1	10	1	43854	07:39	1	121	65	1	83	2	999	2	999	1	2	999	1	100	2	999	3	3	2	1	999	65	1	2	1	999	999	3	2	
797	34	2	4	1	9	1	43833	11:35	1	127	76	1	81	2	999	1	36.9	1	2	999	1	100	2	999	2	2	2	1	999	80	2	1	999	999	03/01/2020	2	1	
798	27	1	2	2	22	1	43849	22:35	1	96	60	1	112	2	999	1	36.7	1	2	999	1	99	2	999	1	2	12	2	999	999	2	1	1	999	999	1	3	
799	23	1	1	3	33	1	43848	16:04	1	100	66	1	101	2	999	1	36.7	1	2	999	1	99	2	999	1	2	2	2	999	999	1	2	1	999	999	1	3	
800	23	1	1	3	32	1	43839	09:57	1	105	64	1	89	2	999	1	36.8	999	2		1	99	2	999	1	2	7	2	999	153	2	2	1	999	999	2	2	

801	22	1	1	1	9	12	43861	18:43	1	115	60	1	98	2	999	1	36.8	1	2	999	1	100	2	999	2	3	3	2	999	92	2	2	1	999	999	2	2	
802	22	1	1	1	8	1	43850	19:51	1	105	65	1	84	2	999	1	37	1	2	999	1	99	2	999	2	2	2	1	999	159	1	2	1	999	999	2	2	
803	19	1	1	3	35	1	43841	20:23	1	111	67	1	119	2	999	1	36.6	999	2		1	99	2	999	3	3	7	1	999	999	2	999	999	999	999	1	3	
804	42	999	4	3	37	2	43843	08:37	1	160	100	1	80	2	999	1	36.1	999	2		1	99	2	999	4	4	12	1	999	38	1	1	1	999	13/01/2020	2	2	
805	42	1	4	3	38	2	43845	07:40	1	112	64	1	87	2	999	1	35.8	2	2		1	99	1	81	1	1	12	1	999	50	2	2	2	999	15/01/2020	2	1	1
806	23	1	3	3	28	1	43838	21:31	1	99	64	1	82	2	999	1	36.1	1	2	999	1	99	2	999	2	2	2	1	999	999	1	1	1	999	999	1	3	
807	26	1	999	3	28	999	43845	14:11	2	999	999	2	999	2	999	2	999	1	2	999	2	999	2	999	4	2	2	2	999	341	2	999	4	20:30	15/01/2020	3	2	
808	20	1	2	2	24	1	43858	09:37	1	118	73	1	114	2	999	1	35.9	1	2	999	1	100	2	999	1	2	2	2	999	999	2	2	1	999	999	1	3	
809	41	1	999	3	39	3	43853	14:04	1	140	100	1	88	2	999	2	999	999	2		2	999	2	999	999	0	0	3	N√O S	999	2	3	2	999	999	1	4	1
810	21	1	1	3	31	1	43841	02:54	1	101	73	1	93	2	999	1	35.7	1	2	999	2	999	2	999	999	0	0	3	N√O S	161	2	3	1	999	11/01/2020	2	4	
811	21	1	1	3	39	6	43839	20:59	1	109	56	2	999	2	999	2	999	1	2	999	2	999	2	999	3	3	2	1	999	999	1	2	1	999	999	1	3	
812	21	1	1	3	39	1	43845	10:31	1	107	57	1	76	2	999	1	35	999	2		1	99	2	999	2	2	7	1	999	999	2	2	2	999	999	1	3	1
813	33	1	1	3	38	2	43832	07:17	1	127	93	1	76	2	999	1	35.2	2	2		2	999	2	999	999	0	0	3	N√O S	999	1	3	1	18:00	02/01/2020	1	4	
814	20	2	2	1	7	1	43859	12:06	1	104	62	1	76	2	999	1	36.9	1	2	999	1	99	2	999	3	3	2	1	999	114	1	2	1	999	999	2	2	
815	26	1	1	1	7	1	43845	13:51	1	100	62	1	74	2	999	2	999	2	2		2	999	2	999	1	2	12	2	999	999	2	2	1	999	999	1	3	
816	26	1	1	1	7	2	43846	07:56	1	106	67	1	85	2	999	1	35.9	1	2	999	1	99	2	999	2	2	2	1	999	999	1	2	2	999	999	1	3	3
817	42	1	3	3	34	1	43850	15:38	1	123	82	1	85	2	999	1	36.7	2	2		1	99	2	999	1	1	12	1	999	42	2	2	1	999	999	2	1	
818	26	1	6	3	39	1	43861	18:08	1	102	63	1	87	2	999	1	36.1	2	2	999	1	99	2	999	1	1	12	1	999	147	2	2	1	999	999	2	1	
819	24	1	4	3	29	1	43843	19:54	1	100	70	1	84	2	999	2	999	1	2	999	2	999	2	999	2	2	2	1	999	999	2	2	1	999	999	1	3	
820	25	1	1	1	12	1	43832	13:49	1	114	63	1	114	2	999	1	38.3	1	2	999	1	99	2	999	3	3	5	1	999	91	1	999	1	999	02/01/2020	2	2	
821	25	1	1	2	14	1	43849	21:48	1	118	57	1	79	2	999	1	36.8	1	2	999	2	999	1	100	2	2	2	1	999	210	2	2	999	999	999	2	2	
822	27	1	1	3	30	1	43838	10:34	1	90	53	1	88	2	999	2	999	1	2	999	1	99	2	999	2	2	2	1	999	999	2	2	1	999	09/01/2020	1	3	
823	37	1	1	3	30	12	43857	21:13	2	999	999	2	999	2	999	2	999	1	2	999	2	999	2	999	1	2	2	2	999	999	1	2	1	999	999	1	3	
824	26	1	1	1	5	1	43845	15:32	1	112	67	1	82	2	999	2	999	2	2		2	999	2	999	2	2	8	1	999	999	2	1	1	999	15/01/2020	1	3	
825	25	1	1	3	39	2	43855	07:57	1	131	80	1	82	2	999	2	999	2	2		1	99	2	999	3	3	2	1	999	999	1	2	2	999	999	1	3	2
826	33	1	3	1	12	1	43853	20:59	1	101	58	1	67	2	999	1	36.7	2	2		1	99	2	999	3	3	2	1	999	999	1	2	1	999	999	1	3	
827	20	1	1	3	40	1	43850	19:01	1	131	79	1	89	2	999	1	37.1	999	2		1	99	2	999	1	2	12	2	999	134	1	2	1	999	999	2	2	
828	20	1	1	3	41	1	43853	20:06	1	123	77	1	100	2	999	1	36.3	2	2		1	99	2	999	2	2	12	1	999	999	2	1	2	999	999	1	3	2
829	20	1	2	1	7	1	43838	22:43	1	91	49	1	94	2	999	1	37	1	2	999	1	99	2	999	1	2	2	2	999	999	1	2	1	999	999	1	3	
830	28	2	2	1	11	1	43853	21:43	1	132	77	1	98	2	999	1	36.8	2	2		1	100	2	999	2	2	12	1	999	999	1	2	1	999	999	1	3	
831	31	1	1	2	25	1	43851	14:07	1	128	72	1	77	2	999	1	36.4	1	2	999	1	99	2	999	2	2	9	1	999	133	2	1	1	17:27	21/01/2020	2	2	
832	31	1	1	2	26	1	43854	16:01	1	116	74	1	71	2	999	1	35.9	2	2		1	99	2	999	1	2	12	2	999	999	1	2	1	999	999	1	3	
833	27	1	1	1	12	1	43834	16:43	1	118	60	1	116	2	999	1	38.4	1	2	999	1	99	2	999	3	3	5	1	999	77	2	2	999	999	05/01/2020	2	2	
834	36	1	2	2	27	9	43840	13:51	1	118	80	1	82	2	999	1	36.7	1	2	999	1	99	2	999	3	3	12	1	999	124	2	1	1	19:00h	10/01/2020	2	2	
835	40	1	7	999	999	9	43845	13:56	1	106	62	1	89	2	999	2	999	1	2	999	1	99	2	999	3	2	4	2	999	999	2	999	4	999	999	1	3	
836	37	2	3	3	39	1	43844	11:59	1	117	68	1	74	2	999	1	36.2	1	2	999	1	98	2	999	2	2	2	1	999	999	2	2	1	999	999	1	3	
837	37	2	3	3	39	1	43847	11:57	1	134	72	1	79	2	999	1	35.9	1	2	999	1	99	2	999	2	3	2	2	999	999	2	2	1	999	999	1	3	
838	26	1	3	2	20	7	43840	13:16	1	101	62	1	101	2	999	1	37	1	2	999	1	99	2	999	2	2	2	1	999	999	1	2	1	999	10/01/2020	1	3	
839	35	2	5	1	6	1	43843	16:57	1	139	93	1	85	2	999	1	36.4	999	2		1	100	2	999	3	3	8	1	999	123	2	999	4	20:13	13/01/2020	3	2	
840	20	1	3	3	32	12	43837	20:10	1	120	80	1	90	2	999	2	999	1	2	999	1	999	2	999	3	3	2	1	999	999	1	2	999	999	999	1	3	
841	20	1	3	3	41	1	43838	11:31	1	113	77	1	83	2	999	2	999	999	2		1	99	2	999	1	2	12	2	999	999	2	2	2	999	999	1	3	1
842	37	2	3	3	38	1	43837	08:55	1	123	68	1	87	2	999	1	36.3	1	1	4	1	99	2	999	3	3	2	1	999	999	2	2	1	999	07/01/2020	1	3	
843	37	999	3	3	39	1	43849	999	1	126	77	1	87	2	999	1	36.7	999	2		1	99	2	999	3	3	7	1	999	999	1	2	1	999	19/01/2020	1	3	
844	28	1	2	3	34	1	43856	18:01	1	110	59	2	999	2	999	1	36.7	1	2	999	1	99	2	999	4	2	6	2	999	79	2	1	1	20:20	26/01/2020	2	1	
845	30	1	3	3	38	1	43850	06:18	1	125	83	1	88	2	999	2	999	2	2		1	99	2	999	4	4	2	1	999	999	1	1	2	999	999	1	3	2
846	29	1	2	1	8	1	43850	00:06	1	126	81	1	79	2	999	1	36.2	1	2	999	1	99	2	999	2	2	8	1	999	999	2	1	1	999	999	1	3	
847	29	1	2	1	6	1	43850	11:55	1	120	75	1	84	2	999	1	36.4	2	2		1	99	2	999	1	2	12	2	999	999	1	2	1	999	999	1	3	
848	38	1	4	3	29	1	43835	14:15	1	102	55	1	105	2	999	1	36.7	1	2	999	1	99	2	999	2	2	2	1	999	999	1	2	1	999	05/01/2020	1	3	
849	30	2	2	3	34	1	43849	19:00	1	102	71	1	80	2	999	1	36.4	1	2	999	1	99	2	999	4	3	2	2	999	73	2	2	999	999	999	2	2	
850	28	1	6	2	13	1	43842	15:48	1	111	62	1	97	2	999	1	36.7	1	2	999	1	99	2	999	1	2	2	2	999	152	1	1	1	999	999	2	2	
851	29	1	2	1	8	1	43855	09:26	1	114	69	1	95	2	999	1	36.3	2	2		1	99	2	999	3	3	12	1	999	999	2	1	2	999	999	1	3	3
852	39	1	3	3	37	10	43833	06:21	1	148	84	1	72	2	999	1	36	2	2		1	99	2	999	1	2	12	2	999	999	2	2	2	999	03/01/2020	1	3	1
853	25	1	2	2	14	1	43833	08:57	1	107	58	1	85	2	999	1	35.2	1	1	6	1	99	2	999	3	3	2	1	999	999	2	1	999	999	999	1	3	
854	25	1	2	2	17	1	43856	10:56	1	109	58	1	100	2	999	1	35.9	1	2	999	1	99	2	999	2	2	2	1	999	999	1	1	1	999	999	1	3	
855	43	1	6	1	12	1	43847	08:52	1	106	53	1	88	2	999	1	36	999	2		1	99	2	999	2	3	8	2	999	999	2	2	1	999	999	1	3	
856	34	1	4	1	12	1	43841	22:13	1	105	66	1	66	2	999	1	36.7	999	2		1	99	2	999	2	2	8	1	999	999	2	1	1	999	999	1	3	
857	21	1	1	3	35	2	43839	08:26	1	114	65	1	86	2	999	1	36.6	2	2		1	99	2	999	1	2	12	2	999	999	1	2	1	999	09/01/2020	1	3	
858	20	2	1	3	29	1	43843	22:11	1	100	59	1	72	2	999	2	999	2	2		2	999	2	999	2	2	2	1	999	999	1	2	1	999	999	1	3	
859	38	1	3	3	34	1	43861	22:17	1	112	62	1	79	2	999	2	999	1	2	999	2	999	2	999	2	2	9	1	999	73	2	1	1	01:00	01/02/2020	2	1	
860	42	1	999	2	14	999	43856	07:31	1	147	87	1	79	2	999	2	999	1	2	999	2	999	2	999	2	2	2	1	999	999	2	2	1	999	999	1	3	
861	43	1	3	1	11	1	43848	09:03	1	108	68	1	74	2	999	1	36.3	1	2	999	1	99	2	999	2	2	2	1	999	999	2	2	999	999	999	1	3	
862	27	1	2	3	38	1	43842	12:37	1	116	76	1	82	2	999	1	35	1	2	999	1	99	2	999	2	2	2	1	999	199	2	1	1	999	999	2	2	
863	27	1	2	3	39	1	43847	18:52	1	105	59	1	98	2	999	1	36.6	2	2		1	99	2	999	2	2	7	1	999	188	1	2	1	999	999	2	2	
864	27	1	2	3	40	1	43850	09:03	1	109	68	1	91	2	999	1	35.6	1	2	999	1	99	2	999	2	3	2	2	999	159	1	2	1	999	999	2	2	
865	27	1	2	3	40	8	43852	23:07	1	117	80	1	92	2	999	2	999	2	2		2	999	2	999	2	3	7	2	999	999	2	1	2	999	999	1	3	2
866	27	1	2	3	30	8	43860	15:00	1	101	56	1	105	2	999	1	35.5	1	2	999	1	99	2	999	2	2	2	1	999	999	1	1	5	999	999	1	3	
867	27	2	3	3	33	12	43853	03:51	1	109	58	1	107	2	999	1	36.4	999	2		2	999	2	999	999	0	0	3	999	99	1	3	3	10:00	23/01/2020	2	4	
868	25	1	2	1	8	1	43838	12:52	1	111	71	1	76	2	999	1	36.5	999	2		2	999	2	999	999	0	0	3	N√O S	999	1	3	1	999	08/01/2020	1	4	

869	31	2	2	3	29	1	43854	14:27	1	111	78	1	90	2	999	1	35.4	1	2	999	1	99	2	999	2	2	2	1	999	999	2	2	1	999	999	1	3	
870	20	1	1	3	38	1	43842	08:23	1	117	86	1	100	2	999	2	999	1	2	999	1	99	2	999	4	4	2	1	999	53	1	2	1	999	999	2	2	
871	18	1	1	3	34	7	43832	16:03	1	112	55	1	91	2	999	2	999	2	2		1	100	2	999	2	2	12	1	999	183	1	999	1	999	02/01/2020	2	2	
872	20	1	1	3	38	1	43842	18:22	1	126	84	1	120	2	999	1	35.6	1	2	999	1	99	2	999	4	4	2	1	999	999	1	2	2	999	999	1	3	2
873	25	1	2	3	40	1	43840	14:14	1	114	70	1	94	2	999	1	36.6	999	2		1	99	2	999	2	2	2	1	999	196	2	1	1	999	999	2	2	
874	25	1	2	3	41	1	43846	08:21	1	111	68	1	111	2	999	1	35.8	2	2		1	99	2	999	2	2	12	1	999	39	2	1	2	999	999	2	1	2
875	25	1	2	3	40	1	43844	10:28	1	115	61	1	97	2	999	1	35.5	999	2		1	99	2	999	3	3	2	1	999	92	1	2	1	999	999	2	2	
876	20	2	1	2	27	1	43858	23:32	1	115	66	1	100	2	999	1	37.3	1	2	999	1	99	2	999	2	2	6	1	999	999	1	1	1	999	999	1	3	
877	37	1	4	1	10	1	43841	12:01	1	125	76	1	89	2	999	1	36.3	1	2	999	1	99	2	999	2	2	2	1	999	999	1	1	1	999	999	1	3	
878	31	1	4	3	36	11	43847	06:39	2	999	999	2	999	2	999	1	36.7	2	2		2	999	2	999	3	2	10	2	999	129	2	2	4	09:50	17/01/2020	3	2	
879	25	1	1	2	25	1	43848	15:12	1	121	76	1	95	2	999	1	35.4	1	1	5	1	100	2	999	3	3	2	1	999	48	2	2	1	19:15	18/01/2020	2	2	
880	21	1	2	3	34	1	43837	15:24	1	129	72	1	133	2	999	1	36.2	1	1	6	1	100	2	999	3	3	2	1	999	144	2	2	1	21:24	07/01/2020	2	2	
881	21	1	1	2	18	12	43852	17:47	1	101	60	1	90	2	999	1	36.6	1	2	999	1	99	2	999	2	2	7	1	999	999	2	1	1	23:25	22/01/2020	1	3	
882	23	1	2	3	38	1	43847	22:16	1	116	81	1	86	2	999	1	36.3	999	2		1	99	2	999	3	3	2	1	999	134	2	1	2	03:30	18/01/2020	2	2	2
883	31	1	1	999	999	1	43832	13:15	1	999	999	2	999	2	999	2	999	999	2		2	999	2	999	1	1	12	1	999	135	2	999	4	15:30	02/01/2020	3	1	
884	27	1	2	3	33	1	43852	15:12	1	99	55	1	100	2	999	1	36.4	2	2		1	99	2	999	1	2	12	2	999	288	2	999	4	19:40	22/01/2020	3	2	
885	27	1	2	3	33	12	43853	20:54	1	95	62	1	110	2	999	1	36.4	2	2		1	99	2	999	1	2	12	2	999	126	2	2	1	999	999	2	2	
886	27	1	2	3	34	12	43860	21:38	1	102	54	1	99	2	999	1	36.4	2	2		1	100	2	999	1	1	12	1	999	112	2	2	1	999	999	2	1	
887	20	2	1	3	38	10	43856	13:18	1	124	71	1	100	2	999	1	37	1	2	999	1	99	2	999	2	2	2	1	999	999	2	2	2	999	999	1	3	1
888	29	2	4	1	9	1	43861	16:42	1	116	70	1	88	2	999	1	36.7	2	2		1	100	2	999	3	3	7	1	999	999	1	2	1	999	999	1	3	
889	30	1	2	1	6	1	43856	09:22	1	132	79	1	82	2	999	1	36.7	2	2		1	99	2	999	2	2	8	1	999	999	2	1	1	999	999	1	3	
890	30	1	2	1	6	1	43858	14:16	1	110	71	1	83	2	999	1	36.7	2	2		1	100	2	999	2	2	8	1	999	240	2	2	1	999	999	2	2	
891	25	2	1	3	34	12	43851	21:25	1	136	86	1	97	2	999	1	36	2	2		1	99	2	999	1	2	12	2	999	999	1	2	1	999	999	1	3	
892	28	1	3	3	37	2	43834	06:23	1	133	93	1	85	2	999	2	999	1	2	999	2	999	2	999	2	3	2	2	999	999	2	1	2	08:15	04/01/2020	1	3	1
893	36	999	1	1	8	1	43843	09:29	1	131	81	1	75	2	999	1	36.3	1	2		1	100	2	999	2	2	2	1	999	174	2	2	1	999	13/01/2020	2	2	
894	33	1	2	1	7	1	43833	13:05	1	130	74	1	77	2	999	1	36.6	1	1	6	2	999	2	999	3	3	2	1	999	999	2	1	1	999	03/01/2020	1	3	
895	26	1	3	3	28	1	43833	17:12	1	112	65	1	91	2	999	1	35.9	2	2		1	100	2	999	3	3	2	1	999	999	1	2	1	999	03/01/2020	1	3	
896	26	1	3	3	30	1	43843	13:00	1	107	56	1	90	2	999	1	36.7	999	2		1	99	2	999	4	4	2	1	999	120	2	1	1	22:00	13/01/2020	2	2	
897	23	1	3	3	41	1	43851	02:23	1	135	95	1	88	2	999	1	35.9	2	2		2	999	2	999	999	0	0	3	N√O S	127	2	3	2	06:15	21/01/2020	3	4	2
898	21	2	1	3	34	1	43859	14:49	1	99	65	1	106	2	999	1	36.3	2	2		1	100	2	999	3	3	2	1	999	999	1	2	2	999	999	1	3	1
899	31	1	2	3	36	1	43856	11:13	1	121	77	1	96	2	999	1	35	1	2	999	1	99	2	999	2	2	7	1	999	187	2	2	1	999	999	2	2	
900	38	1	4	2	20	1	43852	06:41	1	139	79	1	110	2	999	1	36.4	1	2	999	1	99	2	999	1	2	2	2	999	59	1	2	1	999	999	2	1	
901	38	1	4	2	21	1	43854	10:50	1	121	79	1	99	2	999	1	36.3	1	2	999	1	99	2	999	2	3	2	2	999	170	1	2	1	999	999	2	2	
902	30	1	2	2	19	1	43860	14:59	1	134	83	1	89	2	999	1	35.6	1	2	999	1	99	2	999	1	2	2	2	999	999	2	2	1	999	999	1	3	
903	30	1	2	2	19	999	43858	14:58	1	137	82	1	90	2	999	1	36.7	1	2	999	1	100	2	999	1	2	2	2	999	999	2	1	1	999	999	1	3	
904	30	1	2	2	16	1	43835	14:42	1	124	77	1	86	2	999	1	36	1	2	999	1	99	2	999	3	3	2	1	999	43	2	2	1	999	05/01/2020	2	2	
905	26	1	1	3	31	1	43837	05:59	1	115	79	2	999	2	999	2	999	1	2	999	2	999	2	999	2	2	2	1	999	256	2	2	1	14:15	07/01/2020	2	2	
906	18	999	1	999	999	1	43836	999	1	108	58	1	68	2	999	1	36.7	2	2		1	99	2	999	2	2	8	1	999	999	2	2	1	999	06/01/2020	1	3	
907	24	1	3	3	38	1	43848	15:16	1	95	58	1	93	2	999	1	36.1	999	2		1	100	2	999	3	3	2	1	999	999	2	1	2	999	999	1	3	2
908	24	2	2	3	38	1	43832	05:27	1	146	91	1	87	2	999	1	36.8	999	2		2	999	2	999	999	0	0	3	N√O S	177	2	3	999	999	02/01/2020	2	4	1
909	24	1	3	3	37	12	43847	11:47	1	92	57	1	115	2	999	1	35.6	999	2		1	99	2	999	2	2	7	1	999	176	1	2	1	999	999	2	2	
910	20	1	1	2	26	10	43834	10:14	1	96	56	1	86	2	999	2	999	2	2		1	99	2	999	1	1	12	1	999	999	1	1	999	999	04/01/2020	1	3	
911	24	1	4	3	38	1	43836	15:58	1	151	88	1	82	2	999	1	36.1	1	2	999	1	99	2	999	4	4	3	1	999	62	2	2	2	999	999	2	2	1
912	37	1	4	3	36	2	43835	13:05	1	121	62	1	98	2	999	1	36.4	2	2		1	99	2	999	2	2	6	1	999	35	2	1	2	999	999	2	1	1
913	37	1	4	3	35	2	43832	10:54	2	999	999	2	999	2	999	2	999	1	2	999	2	999	1	110	2	2	2	1	999	999	1	999	2	999	999	1	3	
914	35	1	1	3	38	3	43839	12:07	1	149	93	1	83	2	999	1	36.9	1	2	999	1	99	2	999	4	4	7	1	999	103	2	2	999	999	999	2	2	
915	35	1	1	3	38	3	43840	10:44	1	143	83	1	94	2	999	1	37.1	999	2		1	98	2	999	3	3	8	1	999	999	2	1	2	999	999	1	3	1
916	24	1	1	1	9	1	43836	08:56	1	141	65	1	118	2	999	2	999	1	2	999	1	100	2	999	3	3	2	1	999	999	2	2	999	999	999	1	3	
917	25	2	3	3	39	1	43838	14:07	1	89	55	2	999	2	999	2	999	1	2		2	999	1	96	3	3	10	1	999	53	1	1	1	999	08/01/2020	2	2	
918	25	2	3	3	40	1	43843	20:33	2	999	999	2	999	2	999	2	999	1	2	999	2	999	2	999	3	2	2	2	999	282	2	2	1	999	999	2	2	
919	33	1	6	1	11	1	43843	11:33	1	125	73	1	76	2	999	1	36.3	1	1	6	1	100	2	999	3	3	2	1	999	147	2	1	999	999	999	2	2	
920	33	1	6	1	12	1	43846	12:28	1	123	72	1	85	2	999	1	36.5	1	2	999	1	98	2	999	1	2	2	2	999	999	2	2	1	999	999	1	3	
921	24	1	2	1	12	1	43840	22:31	1	100	60	1	77	2	999	2	999	1	2	999	2	999	2	999	3	3	2	1	999	149	1	1	1	999	11/04/2020	2	2	
922	32	1	1	3	28	1	43836	19:06	1	129	77	2	999	2	999	1	36.1	1	2	999	2	999	2	999	2	2	2	1	999	999	1	1	1	999	06/01/2020	1	3	
923	32	1	1	3	31	1	43861	14:34	1	131	87	1	94	2	999	1	35.6	1	2	999	1	99	2	999	1	2	2	2	999	999	1	1	1	999	999	1	3	
924	22	1	4	3	31	1	43858	20:11	1	118	68	1	103	2	999	1	36.3	1	2	999	1	99	2	999	2	2	2	1	999	999	1	2	1	999	999	1	3	
925	31	1	2	1	7	1	43859	08:59	1	95	56	1	71	2	999	1	35.6	1	2	999	1	99	2	999	1	2	2	2	999	999	2	2	999	999	999	1	3	
926	27	1	1	3	40	3	43853	12:49	2	999	999	2	999	2	999	2	999	2	2		2	999	2	999	1	2	12	2	999	999	2	2	2	999	999	1	3	2
927	21	999	2	3	39	1	43844	10:06	1	115	61	1	73	2	999	2	999	999	2		1	99	2	999	1	4	12	2	999	104	2	2	1	999	999	2	2	
928	24	1	2	3	40	1	43848	11:15	1	116	70	1	96	2	999	1	36.4	2	2		2	999	2	999	1	2	12	2	999	106	2	2	1	999	999	2	2	
929	23	999	2	3	40	1	43853	999	1	114	71	1	89	2	999	1	35.9	999	2		1	99	2	999	2	2	2	1	999	999	2	2	1	999	999	1	3	
930	24	1	2	3	41	1	43855	11:14	1	109	68	1	74	2	999	1	36.6	2	2		1	99	2	999	2	2	12	1	999	156	1	1	1	999	999	2	2	
931	24	1	2	3	40	1	43856	01:32	1	130	80	2	999	2	999	2	999	999	2		2	999	2	999	3	3	7	1	999	999	2	1	2	999	999	1	3	2
932	29	1	3	1	11	11	43838	14:52	1	94	59	2	999	2	999	2	999	1	2	999	2	999	2	999	2	2	9	1	999	999	1	2	1	14:00	09/01/2020	1	3	
933	29	1	3	1	12	999	43845	09:48	1	91	60	1	91	2	999	1	35.2	2	2		1	100	2	999	1	1	12	1	999	407	2	999	4	16:25	15/01/2020	3	2	
934	32	2	1	3	39	2	43835	04:41	1	110	70	2	999	2	999	2	999	1	2	999	2	999	2	999	2	3	2	2	999	79	2	2	1	18:00	05/01/2020	2	2	
935	32	2	1	3	40	2	43840	06:21	1	111	62	2	999	2	999	2	999	999	2		2	999	2	999	1	2	12	2	999	999	1	2	2	999	999	1	3	999
936	21	2	2	2	27	8	43848	11:42	1	113	71	1	100	2	999	1	36.5	999	2		2	999	2	999	2	2	6	1	999	133	2	1	999	999	999	2	2	

937	31	1	4	1	6	1	43852	20:53	1	110	70	1	83	2	999	2	999	2	2		2	999	2	999	3	3	8	1	999	999	1	2	1	999	999	1	3	
938	32	1	2	3	30	2	43839	20:48	1	125	79	1	120	2	999	1	36.7	999	2		1	99	2	999	1	2	4	2	999	999	1	2	999	999	999	1	3	
939	18	999	1	2	15	1	43861	07:46	1	113	75	1	104	2	999	1	36.6	1	2	999	1	99	2	999	2	3	2	1	999	999	1	2	1	14:47	31/01/2020	1	3	
940	30	1	3	1	12	1	43839	07:42	1	105	71	1	82	2	999	1	35.4	2	2		1	99	2	999	2	2	12	1	999	153	2	999	4	10:25	09/01/2020	3	2	
941	22	1	2	3	38	1	43836	20:21	1	111	61	2	999	2	999	2	999	1	2	999	2	999	2	999	2	2	2	1	999	999	1	2	1	999	999	1	3	
942	22	1	2	3	39	1	43850	07:58	1	99	64	1	87	2	999	1	36.2	1	2	999	1	99	2	999	3	3	2	1	999	999	2	2	2	09:12	20/01/2020	1	3	2
943	22	1	2	3	39	1	43850	00:39	1	126	81	1	89	2	999	1	35.6	999	2		1	99	2	999	3	3	7	1	999	141	2	2	999	999	999	2	2	
944	23	1	3	2	17	1	43833	12:56	1	94	55	1	74	2	999	1	36.3	1	2	999	1	100	2	999	2	2	2	1	999	999	2	1	1	999	03/01/2020	1	3	
945	28	2	1	3	40	1	43853	18:25	1	142	77	1	79	2	999	1	35.3	2	2		1	99	2	999	3	3	7	1	999	999	2	1	2	999	999	1	3	1
946	23	3	3	3	36	1	43861	11:52	1	86	54	1	96	2	999	1	35.6	1	2	999	1	99	2	999	2	3	2	2	999	298	2	2	1	17:50	31/01/2020	2	2	
947	20	1	1	2	25	1	43833	14:39	1	107	56	1	90	2	999	2	999	1	1	4	1	99	2	999	3	3	2	1	999	999	1	1	1	999	03/01/2020	1	3	
948	20	1	1	999	999	1	43838	13:13	1	98	64	1	89	2	999	1	35.6	1	2	999	2	999	2	999	999	0	0	3	N√O S	26	1	3	1	999	08/01/2020	2	4	
949	39	1	3	3	32	2	43852	07:23	1	106	66	1	92	2	999	1	36.4	2	2		1	99	1	98	1	2	12	2	999	22	2	2	999	999	999	2	1	
950	28	2	1	3	32	3	43832	11:17	1	160	102	2	999	2	999	2	999	1	2	999	2	999	2	999	4	3	3	2	999	43	2	999	2	999	02/01/2020	2	2	2
951	30	2	3	3	39	2	43852	21:20	1	112	66	1	82	2	999	2	999	1	2	999	2	999	2	999	2	2	2	1	999	340	2	2	1	999	999	2	2	
952	27	1	1	2	26	1	43839	23:33	1	111	59	1	75	2	999	2	999	1	2	999	1	99	2	999	4	4	2	1	999	82	2	2	999	999	999	2	2	
953	31	1	2	1	11	1	43859	14:42	1	124	78	1	84	2	999	1	36.7	1	2	999	1	99	2	999	2	2	8	1	999	999	1	2	1	999	999	1	3	
954	21	1	2	3	37	1	43833	14:16	1	113	66	1	67	2	999	2	999	1	1	7	1	99	2	999	4	4	2	1	999	156	2	2	1	18:30	03/01/2020	2	2	
955	21	1	2	3	38	1	43834	08:43	1	124	76	1	74	2	999	1	35.6	1	2	999	1	99	2	999	4	4	2	1	999	999	2	2	2	999	04/01/2020	1	3	1
956	22	1	1	1	8	1	43850	08:49	1	119	70	1	74	2	999	1	36.8	2	2		1	99	2	999	2	3	8	2	999	999	2	2	1	15:07	20/01/2020	1	3	
957	22	1	1	1	7	1	43854	13:08	1	120	70	1	78	2	999	1	36.8	1	2	999	1	99	2	999	2	2	6	1	999	999	2	2	1	999	999	1	3	
958	24	3	2	3	40	1	43838	12:21	1	110	80	2	999	2	999	1	36.7	999	2		2	999	2	999	999	0	0	3	N√O S	999	2	3	2	999	08/01/2020	1	4	1
959	27	1	2	3	30	1	43856	22:08	1	119	79	1	110	2	999	1	37.2	1	2	999	1	99	2	999	1	2	2	2	999	999	1	1	1	999	999	1	3	
