# Supplementary material for: Interobserver agreement in Reception and Risk Stratification in Obstetrics implementation
Source: Rev Bras Enferm. 2024 Dec 13;77(5):e20230361. doi: 10.1590/0034-7167-2023-0361 (PMC11654229; doi:10.1590/0034-7167-2023-0361)
Supplement: Translation

# Translation: Concordância interobservadores na implementação do Acolhimento e Classificação de Risco em Obstetrícia

**DOI:** 10.1590/0034-7167-2023-0361pt

**Authors:** Manuela Beatriz Velho, Luciana Santos Pimentel, Fernanda Amâncio Soares da Silva, Alberto Trapani, Thayná Ventura, Adaiana Fátima Almeida, Roberta Costa, Roxana Knobel

**Affiliations:** I Universidade Federal de Santa Catarina. Florianópolis, Santa Catarina, Brasil

**Keywords:** Obstetrícia, Acolhimento, Triagem, Classificação de Risco, Emergências

## Abstract

**Objetivos:**

analisar a concordância interobservadores na implementação do protocolo de Acolhimento e Classificação de Risco em Obstetrícia.

**Métodos:**

estudo transversal, realizado durante a implementação do Acolhimento e Classificação de Risco em Obstetrícia, conduzido em hospital terciário no sul do Brasil com 891 participantes em janeiro de 2020. Realizou-se análise descritiva e de concordância interobservadores pelo coeficiente de Kappa na classificação de risco atribuída pelo enfermeiro classificador e revisada pelo pesquisador.

**Resultados:**

cerca de metade dos atendimentos (55,6%) foi classificada como pouco urgente (verde), seguida de urgente (amarelo) (31,8%), muito urgente (laranja) (9,3%), não urgente (azul) (3,4%) e nenhuma classificação emergente (vermelha). Análise de concordância da classificação revisada encontrou valores de Kappa 0,20 (azul), 0,54 (verde) 0,77 amarelo e 0,80 (laranja).

**Conclusões:**

a maioria dos atendimentos foi pouco urgente. A análise de concordância entre a classificação de risco revisada e atribuída revelou maior concordância interobservador conforme aumentou o grau de prioridade.

## INTRODUÇÃO

A gestação e o puerpério são fenômenos naturais do ciclo reprodutivo feminino em que ocorrem mudanças fisiológicas significativas e que, na maioria das vezes, evolui sem intercorrências. Entretanto, alguns casos podem apresentar complicações obstétricas, resultando em quadros mórbidos maternos e, eventualmente, repercussões fetais, mesmo em pessoas previamente sadias. Nesses casos, o atendimento em unidades de emergência se faz necessário, sendo essencial o acolhimento por parte dos profissionais de saúde e a classificação de risco obstétrico^(1-3)^.

O atendimento à mulher no ciclo gravídico puerperal deve ser qualificado e ágil, de forma a promover uma assistência eficaz às demandas fisiológicas, como o parto, mas principalmente promover ações que evitem ou reduzam danos ao binômio em casos patológicos. É evidente que falhas na identificação da gravidade do quadro aumentam o risco de morbimortalidade materna, e por isso, como alternativa, surge a triagem obstétrica para minimizar tal desfecho^(1,3-5)^.

A triagem é uma tecnologia desenvolvida em emergências clínicas que objetiva priorizar o atendimento baseado nas condições de saúde do paciente, em sua necessidade por atenção médica e recursos dos serviços de saúde. O sistema de triagem deve ser rápido, de fácil aplicação e com forte poder para predizer gravidade, evolução e utilização de recursos. A implementação de ferramentas com esta finalidade corrobora para maior qualidade no cuidado, organização no fluxo assistencial e na utilização de recursos, além de propiciar atendimento em tempo oportuno e diminuir o tempo de espera^(1,4-6)^.

As emergências obstétricas são a porta de entrada de gestantes e puérperas que procuram atendimento por alterações fisiológicas próprias da gestação ou por processos mórbidos. A *American College of Obstetricians and Gynecologists* recomenda a aplicação da classificação de risco a essa população. Priorizar a assistência em contrapartida ao atendimento por ordem de chegada é necessária, não só devido à superlotação dos serviços de saúde e à demanda que excede a capacidade profissional, mas também por estabelecer parâmetros e fluxos de atendimentos que qualificam o cuidado prestado, além de trazer satisfação às usuárias e às equipe de atendimento^(4-10)^.

Embora a triagem geral esteja fortemente estabelecida nos serviços de emergência, não há na literatura consenso sobre os critérios que devem ser aplicados na triagem obstétrica. É evidente que a triagem obstétrica, quando comparada à geral, é considerada mais complexa, pois requer avaliação das alterações fisiológicas associadas à gestação, bem como análise e consideração de aspectos tanto maternos quanto fetais^(4,8)^.

Devido às especificidades obstétricas, internacionalmente, desenvolveram-se diversas escalas e fluxogramas. Os mais conhecidos são o *Obstetric Triage Acuity Scale* (OTAS), que classifica as pacientes em cinco níveis de gravidade e que parece reduzir o tempo de espera para atendimento, o *Swiss Emergency Triage Scale* (SETS), que classifica as pacientes em quatro níveis, e o *Maternal Fetal Triage Index* (MFTI), que classifica as gestantes em cinco níveis. Destaca-se que todos possuem validade para a população estudada^(4,8)^.

No Brasil, o Ministério da Saúde (MS) lançou, em 2011, a Rede Cegonha, com a finalidade de contemplar os objetivos do Desenvolvimento do Milênio Propostos pela Organização Mundial da Saúde (OMS) para a redução da morbimortalidade materno-infantil. A Rede Cegonha estabeleceu, entre outras ações, a implementação e o funcionamento do Acolhimento e Classificação de Risco (A&CR) em Obstetrícia^(1)^. O A&CR foi desenvolvido a partir do trabalho teórico de uma equipe multidisciplinar do MS e estabeleceu fluxogramas que orientam o profissional de saúde na priorização do atendimento por gravidade, permitindo uma assistência à população de forma mais equânime. No protocolo do MS brasileiro, são determinados cinco níveis de gravidade, com tempos ideais para o atendimento à gestante conforme protocolo estruturado, a partir das diferentes queixas, que se baseiam em dados vitais e na sintomatologia apresentada pela paciente^(1)^.

Em 2020, um hospital universitário localizado na região Sul do Brasil implementou o protocolo do A&CR em Obstetrícia proposto pelo MS brasileiro para organizar o setor de emergência obstétrica. Entende-se que a implementação de um protocolo é um momento oportuno para o acompanhamento avaliativo e ajustes operacionais.

## OBJETIVOS

Analisar a concordância interobservadores na implementação do protocolo de Acolhimento e Classificação de Risco em Obstetrícia.

## MÉTODOS

### Aspectos éticos

O estudo respeitou a Resolução n^o^. 466/2012 do Conselho Nacional de Saúde, e foi aprovado sob o Certificado de Apresentação de Apreciação Ética, emitido por um Comitê de Ética em Pesquisa com Seres Humanos com registro na Plataforma Brasil. O consentimento à participação na pesquisa foi obtido previamente à coleta de dados nas fichas de atendimento, por contato direto da pesquisadora com as mulheres no momento em que aguardavam atendimento médico na sala de espera da emergência obstétrica ou durante a internação na enfermaria do hospital, com assinatura do Termo de Consentimento Livre e Esclarecido. Para garantir o sigilo e anonimato das participantes, foram utilizados códigos numéricos na extração e elaboração do banco de dados, a partir das fichas de atendimento.

### Desenho do estudo

Trata-se de estudo observacional, transversal, realizado durante a implementação do A&CR em Obstetrícia, com coleta de dados retrospectiva por meio dos registros contidos nas fichas de atendimento da emergência obstétrica na maternidade de hospital universitário localizado na região Sul do Brasil. O estudo seguiu as diretrizes do *STrengthening the Reporting of OBservational studies in Epidemiology* (STROBE).

### Período

A coleta de dados foi retrospectiva e ocorreu entre os meses de maio e agosto de 2020, sendo realizada a partir das fichas dos atendimentos na emergência obstétrica referentes ao mês de janeiro de 2020. Os pesquisadores tiveram acesso às fichas, após a conclusão dos atendimentos, no setor de arquivamento dos prontuários do hospital. Não houve interferência durante a coleta de dados sobre o tratamento ou a assistência prestada às usuárias no serviço.

### Local

O local do estudo caracteriza-se como um hospital universitário, terciário, que atende exclusivamente usuários do Sistema Único de Saúde, sistema público e universal brasileiro, localizado na região Sul do Brasil. A instituição é referência em atendimentos obstétricos de alta complexidade no Estado.

### População e amostra

A população do estudo consistiu nas fichas dos atendimentos na emergência obstétrica no mês de janeiro de 2020, período que correspondeu ao mês de implementação do A&CR em Obstetrícia na instituição pesquisada. Para a seleção da amostra, delimitada pelo tempo, foram critérios de inclusão as fichas de atendimentos de gestantes com idade igual ou superior a 18 anos. O critério de exclusão foi a ausência do registro da classificação de risco na ficha de atendimento. No mês de janeiro de 2020, foram identificadas 1.038 fichas de atendimentos; dessas, 79 foram excluídas pelo atendimento de mulheres não gestantes e outras 68 foram excluídas pela ausência do registro de avaliação ou classificação de risco pelo enfermeiro do serviço de A&CR em Obstetrícia. A amostra final foi composta por 891 participantes.

### Protocolo do estudo

A implementação do serviço de A&CR em Obstetrícia, baseado no protocolo do MS, foi precedida pelo treinamento dos enfermeiros classificadores e contou com a participação de profissionais enfermeiros e médicos que atuavam na maternidade. Ao final do treinamento, cinco enfermeiros foram designados para realizar a A&CR em Obstetrícia no hospital investigado, dos quais dois possuíam experiência na área obstétrica, quatro eram do sexo feminino e um era do sexo masculino.

O protocolo do MS para o serviço de A&CR em Obstetrícia determina que as pacientes sejam classificadas de acordo com a gravidade clínica, a partir da queixa principal e exame físico sucinto, composto por 12 fluxogramas que estabelecem a melhor classificação a partir do *status* clínico da usuária^(1)^. Os fluxogramas para a classificação de risco são: desmaio/mal estar geral; dor abdominal/lombar/contrações uterinas; dor de cabeça/tontura/vertigem; falta de ar/sintomas respiratórios; febre/sinais de infecção; náuseas e vômitos; perda de líquido via vaginal/secreções; perda de sangue via vaginal; queixas urinárias; parada/redução de movimentos fetais; relato de convulsão; e outras queixas/situações^(1)^.

De acordo com a classificação de risco, as pacientes são categorizadas em cinco níveis/fluxo de atendimento, identificados por meio de cores, que determinam o tempo para avaliação pela equipe assistencial, quais sejam: vermelha (emergente), risco de morte, atendimento imediato; laranja (muito urgente), estado crítico ou semicrítico não estabilizada, atendimento em 15 minutos; amarela (urgente), estado crítico ou semicrítico estabilizada, atendimento em 30 minutos; verde (pouco urgente), sem riscos de agravos, atendimento em 120 minutos; azul (não urgente), não grave, atendimento em 240 minutos^(1)^.

Na instituição pesquisada, o registro do atendimento acontece em material impresso, denominado ficha de atendimento, sendo preenchido manualmente. Para implementação do A&CR, foram realizados ajustes na ficha de atendimento na emergência obstétrica da maternidade, com a inserção de um espaço para uma breve descrição da anamnese realizada pelo enfermeiro classificador, bem como a delimitação de um espaço para o registro dos sinais vitais, da queixa principal e da investigação sucinta do exame físico.

Para a coleta das informações primárias a partir das fichas de atendimento, os dados foram coletados em formulário *online* desenvolvido em ferramenta virtual disponível no Google Formulários^®^ com as variáveis de interesse. O formulário de coleta de dados foi testado antes da aplicação, sendo realizados ajustes na forma de preenchimento, a fim de evitar a perda de dados.

As variáveis analisadas foram: sociodemográficas e obstétricas (idade, procedência, número de gestações, trimestralidade e comorbidades [nenhuma, diabetes mellitus, síndrome hipertensiva, doença da tireoide, HIV, sífilis, tabagismo, asma/pneumopatia, doença psiquiátrica, infecção urinária recorrente, outras]); aplicação do protocolo A&CR (aferição dos sinais vitais, aferição de outros sinais e sintomas, classificação de risco atribuída (CRA) pelo enfermeiro classificador [vermelha, laranja, amarela, verde, azul], classificação de risco revisada (CRR) [vermelha, laranja, amarela, verde, azul], tempo decorrido entre a chegada ao hospital e a aplicação do protocolo A&CR e/ou o atendimento médico); e desfecho final (alta hospitalar, internação, evasão, não aguardou atendimento, transferência para outra unidade, não informado). A CRR foi realizada após coleta de dados da ficha de atendimento por um dos pesquisadores - médico residente do último ano em obstetrícia que participou do curso de formação para implementação do protocolo - com base nos registros da queixa principal, anamnese e exame físico registrado pelo enfermeiro classificador, conforme o protocolo de classificação de risco.

### Análise dos resultados e estatística

Os dados coletados no Google Formulários^®^ foram transferidos para o programa estatístico *Statistical Package for the Social Sciences* (SPSS^®^). Seguiu-se uma análise descritiva das variáveis acima detalhadas (número absoluto, relativo, intervalo de confiança e medidas de tendência central), bem como uma análise de concordância entre a classificação definida pelos enfermeiros classificadores e a classificação revisada por um dos pesquisadores. Analisaram-se as frequências de sub e superestimação da classificação atribuída pelo enfermeiro classificador de acordo com a classificação revisada pelo pesquisador. Aplicou-se também o índice de Kappa ponderado, sendo adotados os seguintes valores de Kappa: <0, sem concordância; 0 - 0,20, fraco; 0,21 - 0,40, razoável; 0,41 - 0,60, moderado; 0,61 - 0,80, forte; 0,81 - 1,00, perfeito^(11)^.

*A priori*, a delimitação da amostra foi conduzida pelo tempo, ou seja, todas as gestantes que atenderam aos critérios de inclusão e exclusão, atendidas na emergência obstétrica da maternidade, no primeiro mês de implementação do A&CR em Obstetrícia. Para avaliar o poder da amostra na identificação de diferenças interobservadores, realizou-se um cálculo amostral posteriormente à análise de dados. Foi considerado o percentual de discordância entre as avaliações interobservadores por meio do índice Kappa encontrado, do erro alfa e erro beta, e foi verificada a necessidade de um tamanho mínimo de 314 avaliações de A&CR para a condução deste estudo^(12)^.

## RESULTADOS

A caracterização sociodemográfica e obstétrica mostrou uma idade média de 27,4 anos, com maior prevalência de gestantes procedentes do município de localização do hospital (78,7%), sem comorbidades (74,1%) e que estavam no terceiro trimestre de gestação (61,2%) (Tabela 1).

**Tabela 1:** Características sociodemográficas e obstétricas das gestantes atendidas no Acolhimento e Classificação de Risco em Obstetrícia em um hospital universitário (N=891), Região Sul do Brasil, 2020

Idade em anos	Média (DP)	Moda	Mínimo	Máximo
	27,4 (+ 6,0)	25	18	49
	n		% (IC 95%)	
Procedência				
Município do hospital	701		78,7 (75,9 - 81,2)	
Região Metropolitana	138		15,5 (13,3 - 18,0)	
Outras regiões	23		2,6 (1,7 - 3,9)	
Não informado	29		3,3 (2,3 - 4,6)	
Número total de gestações				
Primigesta	337		37,8 (34,7 - 41,1)	
Secundigesta	243		27,3 (24,4 - 30,3)	
Mais de três gestações	300		33,7 (30,6 - 36,9)	
Não informado	11		1,2 (0,7 - 2,2)	
Comorbidades				
Nenhuma	660		74,1 (71,1 - 76,9)	
Diabetes mellitus	60		6,7 (5,3 - 8,6)	
Síndromes hipertensivas	44		4,9 (3,7 - 6,6)	
Doença da tireoide	11		1,2 (0,7 - 2,2)	
HIV	3		0,3 (0,1 - 1,0)	
Sífilis	5		0,6 (0,2 - 1,3)	
Tabagismo	10		1,1 (0,6 - 2,1)	
Asma/pneumopatia	12		1,3 (0,8 - 2,4)	
Doença psiquiátrica	8		0,9 (0,5 - 1,8)	
Infecção urinária/pielonefrite	15		1,7 (1,0 - 2,8)	
Outras	55		6,2 (4,8 - 8,0)	
Não informado	8		0,9 (0,5 - 1,8)	
Trimestre gestacional				
Primeiro	172		19,3 (16,8 - 22,0)	
Segundo	154		17,3 (14,9 - 19,9)	
Terceiro	545		61,2 (57,9 - 64,3)	
Não informado	20		2,2 (1,5 - 3,5)	

A principal queixa no fluxograma do protocolo de A&CR em Obstetrícia do MS brasileiro foi dor abdominal/lombar/contrações uterinas, presente em 444 atendimentos (49,8%) (Tabela 2).

**Tabela 2:** Queixa principal das gestantes atendidas conforme fluxograma de Acolhimento e Classificação de Risco em Obstetrícia do Ministério da Saúde do Brasil em um hospital universitário (N=891), Região Sul do Brasil, 2020

Queixa principal	n	% (IC 95%)
Desmaio/mal estar geral	3	0,3 (0,1-1,0)
Dor abdominal/lombar/contrações uterinas	444	49,8 (46,5-53,1)
Dor de cabeça/tontura/vertigem	27	3,0 (2,1-4,4)
Falta de ar/sintomas respiratórios	3	0,3 (0,1-1,0)
Febre/sinais de infecção	15	1,7 (1,0-2,8)
Náuseas e vômitos	33	3,7 (2,6-5,2)
Perda de líquido via vaginal/secreções	77	8,6 (7,0-10,7)
Perda de sangue via vaginal	93	10,4 (8,6-12,6)
Queixas urinárias	23	2,6 (1,7-3,9)
Parada/redução de movimentos fetais	37	4,1 (3,0-5,7)
Relato de convulsão	0	-
Outras queixas/situações	136	15,3 (13,0-17,8)

Quanto ao atendimento pela equipe médica assistencial da emergência, havia o registro em 5,7% (51 atendimentos) do horário de início, dos quais cinco foram classificados como azul, 34, como verde, nove, como amarelo, e três, como laranja. A média de tempo entre a chegada das gestantes na recepção do hospital e o atendimento pela equipe médica assistencial nesses atendimentos foi de 181 minutos, com um mínimo de 24 e um máximo de 420 minutos de espera.

Na análise da aferição dos sinais vitais e outros sinais e sintomas, conforme chaves de decisão dos fluxogramas, obtiveram-se as seguintes observações: em 867 gestantes, foi aferida pressão arterial sistêmica (97,3%); em 807, foi aferida a frequência cardíaca (90,6%); em 696, foi aferida a temperatura axilar (78,1%); e em 718, foi verificada a saturação periférica de oxigênio (80,6%). Outros sinais e sintomas avaliados foram, em cinco casos, a frequência respiratória (0,6%); em 48 casos, foi aplicada a Escala Analógica Visual da dor (5,4%); e em 21 atendimentos, foi realizado o hemoglicoteste (2,4%).

A Tabela 3 apresenta a CRA pelo enfermeiro classificador e a CCR pelo pesquisador. O A&CR, aplicado por enfermeiro treinado em 891 casos, resultou na classificação de 30 (3,4%) casos na cor azul, 495 (55,6%) na cor verde, 283 (31,8%) na cor amarela, 83 (9,3%) na cor laranja e não houve classificação na cor vermelha. Na CRR pelo pesquisador, 164 gestantes foram classificadas na cor azul (18,4%), 385, na cor verde (43,2%), 249, na cor amarela (27,9%), 93, na cor laranja (10,4%), e novamente, não houve classificação na cor vermelha.

**Tabela 3:** Classificação de risco atribuída e revisada de gestantes atendidas no Acolhimento e Classificação de Risco em Obstetrícia em um hospital universitário (N=891), Região Sul do Brasil, 2020

		Classificação de risco atribuída					
		**Azul**	**Verde**	**Amarela**	**Laranja**	**Vermelha**	**Total** **n (%)**
**Classificação de risco revisada**	Azul	28	129	5	2	0	164 (18,4%)
	Verde	2	338	41	4	0	385 (43,2%)
	Amarela	0	21	223	5	0	249 (27,9%)
	Laranja	0	7	14	72	0	93 (10,4%)
	Vermelha	0	0	0	0	0	0 (-)
	Total n (%)	30 (3,4%)	495 (55,6%)	283 (31,8%)	83 (9,3%)	0 (-)	891 (100,0%)

Portanto, houve discordância entre a CRA e a CRR. Observa-se, entre os casos discordantes, que a revisão do pesquisador identificou 164 casos alocados na cor azul (18,4%); desses, 28 casos foram concordantes com CRA pelo enfermeiro classificador e em 136 casos foram discordantes. Entre os casos discordantes, 129 atendimentos tinham sido classificados na cor verde, cinco atendimentos, na cor amarela, e dois atendimentos, na cor laranja, pelo enfermeiro classificador (Tabela 3).

A partir da CRA pelo enfermeiro classificador, a Tabela 4 apresenta as taxas de subestimação e superestimação da classificação, comparada com a CRR pelo pesquisador. Nota-se, por exemplo, que houve uma subestimação de 21 fichas de atendimento nas classificações de cor amarela (2,4%), que foram classificadas na cor verde. Da mesma forma, foi encontrada uma superestimação de 136 fichas de atendimento na classificação de cor azul (15,3%) pelo enfermeiro classificador, quando lhes foi atribuída uma classificação verde na revisão.

**Tabela 4:** Cálculo de subestimação e superestimação da classificação de risco atribuída e revisada de gestantes atendidas no Acolhimento e Classificação de Risco em um hospital universitário (N=891), Região Sul do Brasil, 2020

	Subestimação da classificação pelo enfermeiro classificador		Superestimação da classificação pelo enfermeiro classificador	
	**n**	**%**	**n**	**%**
Azul	-	-	136	15,3
Verde	2	0,2	45	5,0
Amarela	21	2,4	5	0,6
Laranja	21	0,0	-	-
Vermelha	-	-	-	-
Total	44	2,5	184	20,8

A Tabela 5 mostra a concordância entre a CRA e CRR, com os respectivos valores de Kappa ponderado por grau de prioridade. Os resultados mostram uma elevação do grau de concordância conforme aumenta o grau de prioridade: fraca, na cor azul; moderada, na cor verde; e forte, nas cores amarela e laranja.

**Tabela 5:** Concordância entre classificação de risco atribuída e revisada de gestantes atendidas no Acolhimento e Classificação de Risco em um hospital universitário (N=891), Região Sul do Brasil, 2020

Grau de prioridade	Cor	Fichas classificadas		Classificação discordante		Kappa	Valor de *p*
		n	%	n	%		
1	Azul	30	3,4	2	0,2	0,20	<0,001
2	Verde	495	55,6	157	17,6	0,54	<0,001
3	Amarela	283	31,8	60	6,7	0,77	<0,001
4	Laranja	83	9,3	11	1,2	0,80	<0,001
5	Vermelha	-	-	-	-	-	-
Total		891	100	230	25,7	0,68	< 0,001

Da totalidade dos atendimentos avaliados pelo A&CR, observou-se que 574 (64,4%) resultaram em alta médica; 187 (21,0%) levaram a internações hospitalares; 18 (2,0%) gestantes evadiram; 45 (5,1%) não aguardaram atendimento médico; três (0,3%) dos casos foram transferidos para outras unidades de saúde; e 64 casos (7,2%) não possuíam informação do desfecho. Entre as 891 pacientes atendidas na emergência obstétrica, 156 evoluíram para nascimento (parto vaginal ou cesariana), o que representa 17,5% de todos os atendimentos analisados neste estudo; e ainda, 45 (5,1%) foram internadas em enfermaria de alto risco; e 18 (2,0%) foram submetidas ao esvaziamento uterino por abortamento.

## DISCUSSÃO

As gestantes atendidas na maternidade do hospital universitário tinham idade média de 27,4 anos, sendo maior a prevalência de gestantes procedentes do município de localização do hospital, sem comorbidades e que estavam no terceiro trimestre de gestação. Elas buscaram atendimento, principalmente, devido à queixa de dor abdominal/lombar/contrações, sendo classificadas em sua maioria na cor verde, atendimento pouco urgente, e receberam alta hospitalar após o atendimento. Perfil semelhante foi observado em outros locais da região Sul do Brasil. No estado do Rio Grande do Sul, estudo conduzido em maternidade de risco habitual identificou que 63,2% das pacientes procuraram o serviço com a queixa principal de dor abdominal/lombar/contrações uterinas, e 64,4% tiveram como desfecho a alta hospitalar^(7)^. Já no estado do Paraná, em maternidade de risco habitual, 31,9% das pacientes tinham a mesma queixa e foi maior a prevalência de mulheres entre 20 e 29 anos^(13)^.

Nesta amostra, dos 891 atendimentos na emergência obstétrica, 21,0% resultaram em internação para tratamento de intercorrências ou para resolução da gestação. Do total de atendimentos realizados no serviço, 17,5% evoluíram para o nascimento (partos vaginais ou cesarianas), principal motivo de procura das gestantes pelos serviços de maternidade. Em concordância com outros estudos, vê-se que a demanda nas emergências obstétricas em hospitais terciários é de indivíduos com pouca urgência, que poderiam ter suas solicitações atendidas em serviços de menor complexidade^(7,14,15)^.

Na avaliação dos atendimentos de emergência obstétrica no estudo conduzido no Paraná, 23,2% dos atendimentos retornaram para o serviço de saúde no qual estavam vinculados, seja na Atenção Primária à Saúde ou no serviço de referência, e o menor motivo de encaminhamento desses para o serviço da maternidade resultou na internação por motivo de trabalho de parto (0,02%)^(13)^. O *American College of Obstetricians and Gynecologists* aponta que o atendimento nas emergências obstétricas pode exceder entre duas e cinco vezes o volume de nascimentos em maternidade, situação que pode levar à superlotação dos serviços^(16)^.

Na realidade brasileira, diversas foram as situações apontadas que descrevem esse fenômeno, podendo citar: o baixo vínculo das gestantes com as Unidades Básicas de Saúde, sugerindo uma fragilidade no pré-natal; o entendimento popular de que exames e avaliações podem ser mais facilmente realizados em serviços terciários; uma baixa educação em saúde quanto às alterações fisiológicas da gestação e medidas para sanar ou aliviar estes sintomas; além do sentimento de insegurança e medo^(1,7,14)^. Essa demanda, que poderia ser atendida por Unidades Básicas de Saúde, acaba por gerar uma sobrecarga às emergências. Isso pode implicar prejuízo tanto aos pacientes, uma vez que indivíduos com maior gravidade podem ter seu atendimento deficiente, quanto aos profissionais, que se sentem sobrecarregados e incapazes de prestar a assistência adequada^(7,8,14,15,17-19)^.

A incapacidade das instituições em absorver a demanda resulta em atraso no atendimento. A falta de profissionais, a escassez de equipamentos, insumos e protocolos para o manejo comprometem a assistência^(15,20,21)^. Da mesma forma, a mera implementação de um protocolo de classificação de risco por si só não qualifica o trabalho. Essa tecnologia de saúde leve-dura demanda acolhimento à queixa trazida pela mulher, uma *expertise* do enfermeiro classificador na avaliação da usuária manifestada por uma anamnese e exame físico preciso e sucinto, auxiliando na determinação da gravidade e classificação de risco embasado nos fluxogramas estabelecidos no protocolo^(1,21,22)^.

A partir disso, a educação permanente e a qualificação dos profissionais são essenciais para que o protocolo A&CR seja efetivo^(20,21,23)^. Para isso, é necessário que haja sensibilização por parte dos gestores, para que ofereçam capacitações, atualizações e treinamentos aos profissionais, além de infraestrutura e recursos adequados para a realização do A&CR nas emergências obstétricas^(2,15,21)^.

Os resultados deste estudo demonstraram uma variação na aferição dos sinais e sintomas, com maior frequência de aferição para a pressão arterial e baixa frequência para a Escala Analógica Visual da dor. Esses dados sugerem a utilização de diferentes critérios pelos enfermeiros classificadores, o que demonstra uma potencial fragilidade na aplicação do protocolo e a necessidade de maior treinamento dos enfermeiros classificadores para a aplicação do A&CR^(1)^.

A aferição dos sinais vitais é componente obrigatório dos fluxogramas que conduz as chaves decisórias para definir a classificação mais adequada ao *status* clínico do paciente. É importante ressaltar que os enfermeiros classificadores podem ter aferido os sinais vitais e aplicado a Escala Analógica Visual da dor, mas não ter realizado o registro dessas informações, utilizadas na revisão da classificação pelo pesquisador. Estudo realizado em Belo Horizonte-Brasil, em serviço de emergência geral para adultos, encontrou situação semelhante quanto à aferição dos sinais vitais, com ausência de registro de dados vitais em mais da metade dos casos e com achados semelhantes para aferição da intensidade da dor^(24)^. A aferição e o registro dos sinais vitais e sintomas estabelecidos nos fluxogramas, assim como sua intensidade e duração, são dados objetivos importantes que contribuem para a classificação mais apurada do paciente e, assim, para a determinação da sua gravidade e prioridade de atendimento^(24)^.

Neste estudo, observou-se nas classificações não concordantes com o A&CR um percentual maior na superestimação de gravidade (20,8%) e menor na subestimação (2,5%). Em estudo conduzido na Suécia, os autores encontraram o oposto, um percentual menor na superestimação nos casos (9,3%) e um percentual maior na subestimação nos casos de maior gravidade (21,1%), ambos na triagem obstétrica^(4)^. Se, por um lado, a subestimação da gravidade pode trazer prejuízos ao indivíduo, na medida em que se subestima seu risco e aumenta seu tempo de espera por atendimento assistencial, potencializando o agravamento de sua condição de saúde, por outro, a superestimação sobrecarrega o serviço de emergência e a equipe assistencial, pois desvia esforços materiais, estruturais e de recursos humanos para paciente com menor gravidade^(24-26)^.

O treinamento continuado do enfermeiro classificador quanto ao protocolo institucional de classificação e quanto às características particulares da população obstétrica, assim como a análise de concordância intra e interobservador rotineira, permite que a aplicação da classificação de gravidade seja mais precisa, com redução de subestimação e superestimação. Desta forma, o atendimento será realizado em tempo adequado e com os recursos necessários às demandas apresentadas pelo paciente^(2,5,11,25,26)^.

O desenvolvimento e a implementação de um sistema de triagem obstétrica na Suécia, em sua avaliação inicial, demonstraram uma boa correlação entre a necessidade de internação e o nível de precisão da ferramenta desenvolvida^(4)^. Na comparação, a revisão da classificação de risco neste estudo apontou uma maior concordância interobservador para os atendimentos de maior gravidade, com um índice Kappa forte, enquanto que, para os casos não urgentes, o índice de Kappa foi fraco. Resultados semelhantes foram encontrados na avaliação do A&CR tanto em emergência adulto, em São Paulo-SP, quanto em emergência pediátrica, em estudo conduzido em Fortaleza-Ceará^(25,26)^.

Estudo conduzido no país investigou a concordância na aplicação do protocolo do MS em emergências obstétricas em maternidade escola em Belo Horizonte-MG e avaliou a concordância interavaliadores com a participação de 20 enfermeiros, sendo dez treinados em A&CR e dez não treinados. Como resultado, o coeficiente Kappa variou entre 0,47 e 0,77, com uma tendência à subestimação no grupo vermelho e amarelo, e superestimação nas prioridades clínicas amarelo e verde. Entretanto, o nível de concordância final entre enfermeiros treinados e não treinados foi elevado (0,87), resultado que sugere a confiabilidade no uso e aplicação desse protocolo^(11)^.

Já em estudo internacional, realizado nos Estados Unidos, a investigação da confiabilidade interavaliadores da escala MFTI, com cinco níveis de prioridade, treinou dez enfermeiros para a aplicação da escala e avaliou 211 gestantes triadas. Quando comparadas as classificações dadas pelos enfermeiros participantes e a pesquisadora, foi identificada uma maior discordância entre os níveis três e quatro, de menor gravidade, com 21 casos (10,0%) sendo superestimados pelos enfermeiros e quatro (1,9%) subestimados. A partir disso, com o objetivo de mensurar a concordância interavaliadores, foi utilizado o coeficiente Kappa. Neste estudo, foi alcançada a pontuação Kappa ponderada (0,65), e os autores concluíram que existe confiabilidade na aplicação da escala MFTI^(27)^.

A implementação do A&CR em Obstetrícia na instituição estudada contou com profissionais com variados graus de experiência (desde inexperientes até com *expertise* na área obstétrica), com implementação e atuação simultânea no A&CR em Pediatria, características que podem ter comprometido o conhecimento quanto às nuances do protocolo. Reitera-se que a habilidade dos profissionais a partir da experiência clínica auxilia na avaliação dos fluxogramas e na tomada de decisão, enquanto que a inexperiência pode levar a interpretações subjetivas. Outrossim, por envolver uma população com especificidades singulares, os estudos apontam ser fundamental, para o funcionamento adequado da tecnologia “triagem obstétrica”, que o profissional seja experiente e continuamente treinado para, com isso, potencializar a precisão da classificação de risco em gestantes que chegam à emergência obstétrica, a fim de proteger o binômio e agilizar o atendimento dos casos de maior gravidade^(3,5,7,8,23,28)^.

O treinamento, acompanhamento e avaliação quanto à sua implementação são essenciais para a qualidade do A&CR nos serviços. Tecnologias de informação e comunicação estão cada vez mais sendo testadas e implementadas na assistência à saúde. Estudo que avaliou a construção de um sistema de apoio à decisão de enfermeiros na realização do A&CR em Obstetrícia, baseado no protocolo do MS brasileiro, com perguntas e respostas estruturadas a partir dos sinais e sintomas apresentados pela gestante, obteve índice de adequação satisfatório na avaliação por enfermeiros e profissionais da informática^(29)^.

Na instituição pesquisada, a ausência no registro de dados referentes ao tempo para realização da triagem e o registro deficiente do tempo entre a classificação de risco e a avaliação da equipe assistencial apontam a fragilidade na implementação do protocolo. A avaliação desses indicadores é sugerida pelo MS como critério de qualidade para implementação e monitorização dessa ferramenta^(1)^.

A proposta de um sistema de triagem específico para gestantes tem o potencial de reduzir o tempo de internação e a morbimortalidade materna e neonatal^(1)^, mas, para isso, é preciso realizar um acompanhamento rigoroso dessa ferramenta quanto à sua implementação e implantação, e, com isso, atingir sua finalidade, que é priorizar a assistência breve aos pacientes de maior gravidade^(1,16,19,22)^. Assim, devem-se estabelecer registros claros, avaliação periódica dos indicadores e contínuo aprimoramento das habilidades do enfermeiro classificador^(1,8,18,30)^.

Enquanto política pública de saúde, a Rede Cegonha reconhece a crescente importância da enfermagem obstétrica e seu impacto na saúde das mulheres e recém-nascidos, principalmente na atenção ao parto e nascimento. A partir dos resultados deste estudo e dos dados nacionais apresentados, percebe-se a importância da aplicação do A&CR em Obstetrícia como meio de melhorar a qualidade da assistência, ao organizar o fluxo de atendimento e o uso de recursos. No entanto, é notável a necessidade de uma avaliação sólida sobre a acurácia e precisão da ferramenta de A&CR em Obstetrícia proposta pelo MS.

Os resultados também indicam a necessidade de maiores progressos a serem feitos, já que não foram alcançados os Objetivos do Desenvolvimento do Milênio acordados internacionalmente quanto à redução da mortalidade materna e novamente pactuados nos Objetivos do Desenvolvimento Sustentável. Neste momento, os desafios persistentes na realidade brasileira se apresentam especialmente no que diz respeito à ampliação do acesso aos serviços de saúde essenciais e à redução da mortalidade materna^(31)^. Nesse aspecto, os resultados deste estudo desempenham um papel fundamental no estímulo ao diálogo e em direção ao avanço na assistência em rede, na identificação dos casos de maior gravidade e no impacto para a redução da morbimortalidade materna e neonatal.

### Limitações do estudo

O estudo enfrentou limitações devido à operacionalização da coleta de dados de forma retrospectiva, com informações dos prontuários; portanto, assume-se o viés de informação na eventual falta de registros completos na ficha de atendimento. Outra limitação se deve à ausência no registro do tempo entre a chegada das gestantes na recepção do hospital e o atendimento para realização do A&CR, assim como do horário de atendimento pela equipe médica, impossibilitando a análise desses dados.

No entanto, é estudo inicial realizado em maternidade com atendimento exclusivo pelo Sistema Único de Saúde brasileiro, que trouxe contribuições para a área, ao descrever as características das pacientes atendidas, apontar as fragilidades na implementação e implantação do protocolo de A&CR em Obstetrícia proposto pelo MS brasileiro, bem como as discordâncias interobservadores quanto à aplicação da classificação.

### Contribuições para a área da enfer magem, saúde e política pública

Acredita-se que o uso adequado do A&CR em Obstetrícia poderá reduzir desfechos obstétricos e neonatais adversos, tendo em vista a priorização dos casos graves, mas não se pode concluir esta assertiva, na condução de estudo não intervencionista e diante das dificuldades descritas na implementação dessa ferramenta na instituição. Contudo, o estudo apontou a necessidade de uma avaliação contínua tanto da implementação do protocolo de A&CR em Obstetrícia quanto da necessidade de capacitação do enfermeiro classificador na avaliação e classificação de risco dessas pacientes.

Esta análise evidenciou ainda que a busca de atendimento por pacientes pouco urgentes ou não urgentes a um hospital terciário e de alta complexidade requer maior pactuação entre os diferentes níveis de atenção, a fim de evitar a superlotação da emergência obstétrica e, com isso, uma assistência deficiente às pacientes graves e urgentes. Essas observações foram compartilhadas com a gestão do serviço da maternidade, para a implementação de melhorias no serviço.

## CONCLUSÕES

Este estudo identificou uma maior prevalência na clientela atendida em hospital universitário de nível terciário na atenção à saúde de gestantes sem comorbidades no terceiro trimestre de gestação e que procuraram a emergência com queixa de dor abdominal/lombar/contrações. Na CRA, praticamente metade dos atendimentos foi classificada na cor verde (pouco urgente), e a maior parte resultou em alta hospitalar, o que demonstra que as gestantes poderiam ser atendidas em serviços de menor complexidade. Apenas um quinto dos atendimentos evoluiu para o nascimento (partos vaginais ou cesarianas), com esse sendo o principal motivo de procura das gestantes pelos serviços de uma maternidade. A análise de concordância entre a CRR e CRA revelou maior concordância interobservador conforme aumento na gravidade de casos não urgentes para casos urgentes.

## Data Availability

https://doi.org/10.48331/scielodata.6L3OWP
